# Peptide Recognition Sequence Guides Catalytic Side Chain Cross-Linking of Plant Peptides by Copper-Dependent Cyclases

**DOI:** 10.1021/jacs.4c15470

**Published:** 2025-06-05

**Authors:** Stella T. Lima, Michael A. Pasquale, M. Rafiul O. K. Noyon, Elizabeth A. Clark, Corinne R. Laws, Shabnam Hematian, Jonathan R. Chekan

**Affiliations:** Department of Chemistry and Biochemistry, 14616 University of North Carolina at Greensboro, Greensboro, North Carolina 27402, United States

## Abstract

Side chain cross-linking of amino acids is a challenging oxidative enzymatic reaction that has largely been confined to heme and iron sulfur cluster containing enzymes. However, the recent discovery of plant BURP-domain peptide cyclases (BpCs) responsible for the biosynthesis of burpitide natural products has demonstrated that copper-dependent enzymes are able to generate similar cross-links. For example, ArbB2 is responsible for the formation of a Tyr-phenol-O to Leu-C_β_ bond observed in arabipeptin A, a cyclopeptide alkaloid isolated from the well-known plant Coffea arabica. To investigate this intriguing enzyme family in more detail, we developed minimal peptide substrates for ArbB2, which enabled quantitative studies. By examining reductant dependence, we achieved catalytic turnover for the first time in this enzyme family, which allowed for kinetic, mutational, substrate scope, and multicore processing analyses. Additionally, we established the dioxygen dependence and confirmed the absence of hydrogen peroxide as a side product in the catalytic system. Finally, we extended our study to other BpCs involved in cyclopeptide alkaloid biosynthesis, demonstrating that our findings apply across additional members of this enzyme family. Ultimately, this work provides fundamental insights into a new, widespread family of copper-dependent peptide cyclases and lays the groundwork for future mechanistic investigations.

## Graphical abstract

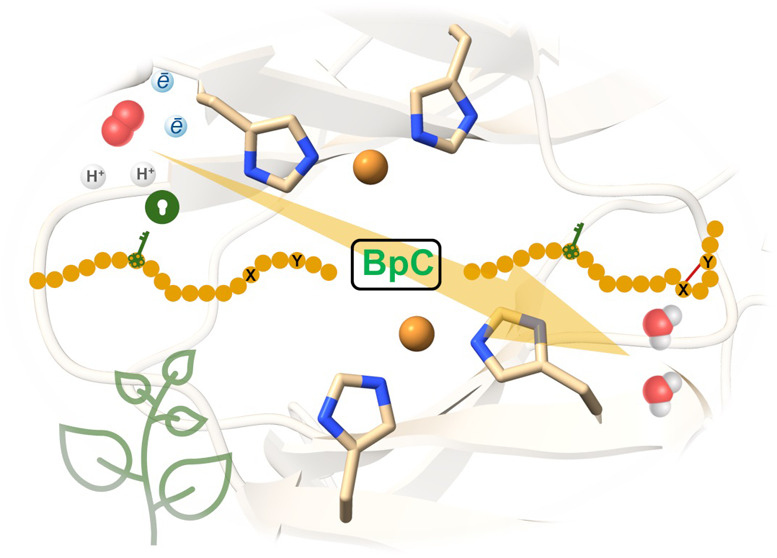

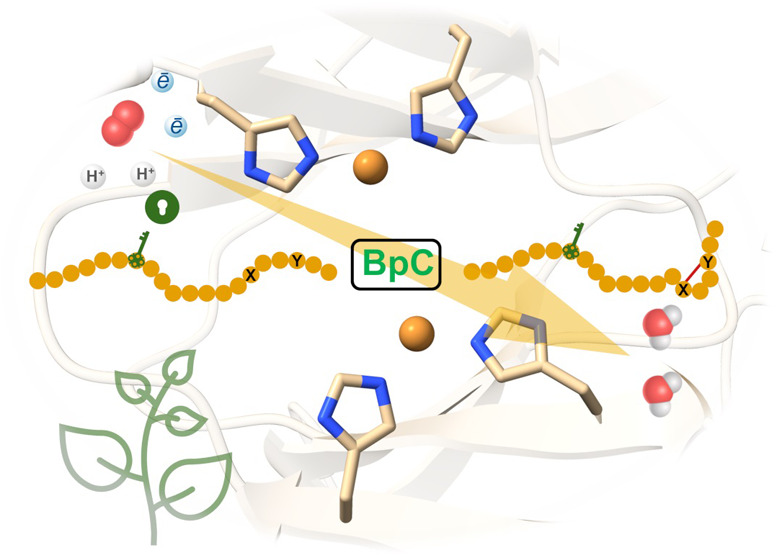

## Introduction

Cyclic peptides constitute an expansive natural product motif, rich in bioactive molecules and distributed across the kingdoms of life. While cyclization of peptides generally offers many valuable biochemical traits such as rigidity and resistance against proteolysis, the chemical details of the cyclization vary widely. For example, the ribosomally synthesized and post-translationally modified peptide (RiPP) superclass of natural products is known to comprise molecules with N- to C-terminal cyclizations, isopeptide bonds, and backbone heterocycle formation.^1^ Macrocyclization through side chain cross-links is also common and can be differentiated into nonaromatic side chain cross-linking such as seen in lanthipeptides^2^ and graspitides,^3^ and side chain cross-linking involving aromatic residues,^4^ observed in triceptides,^5,6^ and biarylitides.^7,200^ In the latter, at least one aromatic ring will be covalently linked to the side chain of another amino acid through C–O, C–C, or C–N bonds.^6,8^ In contrast to dehydration-based mechanisms observed in the some RiPP cyclizations, side chain cross-linking pathways typically involve proton coupled electron transfer (PCET) events, the formation of radical intermediates, and subsequent radical recombination.

Currently, multiple distinct enzyme families are known to catalyze side chain cross-linking reactions involving aromatic residues in peptides:^4^ cytochromes P450 (P450s),^8–14^ radical *S*-adenosyl l-methionine (rSAM) superfamily,^6,15–17^ UstYs (DUF3328s),^18,19^ and burpitide cyclases/BURP-domain peptide cyclases (BpCs).^20–26^ Each of these families utilizes a different catalytic strategy. For example, P450s utilize a heme cofactor that proceeds through a highly oxidizing oxoiron­(IV) ligand-based radical intermediate termed compound I which can abstract hydrogen atoms^27^ to facilitate the biaryl coupling observed in multiple classes of RiPPs including biarylitides,^8,10,11^ atropopeptides,^9^ and cittilins^28^ (Figure 1). A second well studied class of side chain cross-linking enzymes is the rSAM enzyme superfamily. Usually dioxygen (O_2_) sensitive, these enzymes bind one or more [4Fe-4S] clusters and are unified by the formation of the highly reactive 5′-deoxyadenosyl radical from *S*-adenosyl l-methionine. This radical species is able to abstract hydrogen atoms, yielding the cross-links observed in triceptide,^15^ *Streptococcus*-derived,^17,29^ and darobactin^30,31^ families of RiPPs (Figure 1). Finally, in vitro activity for the domain of unknown function (DUF) 3328/UstY family of enzymes has recently been achieved.^19,32^ These fungal enzymes use a dicopper active site to catalyze oxidative transformations such as the Tyr-phenol-O to Phe-C_β_ ether cross-link found in asperipin-2a, in an oxygen dependent manner (Figure 1).^19,33^

**Figure 1:**
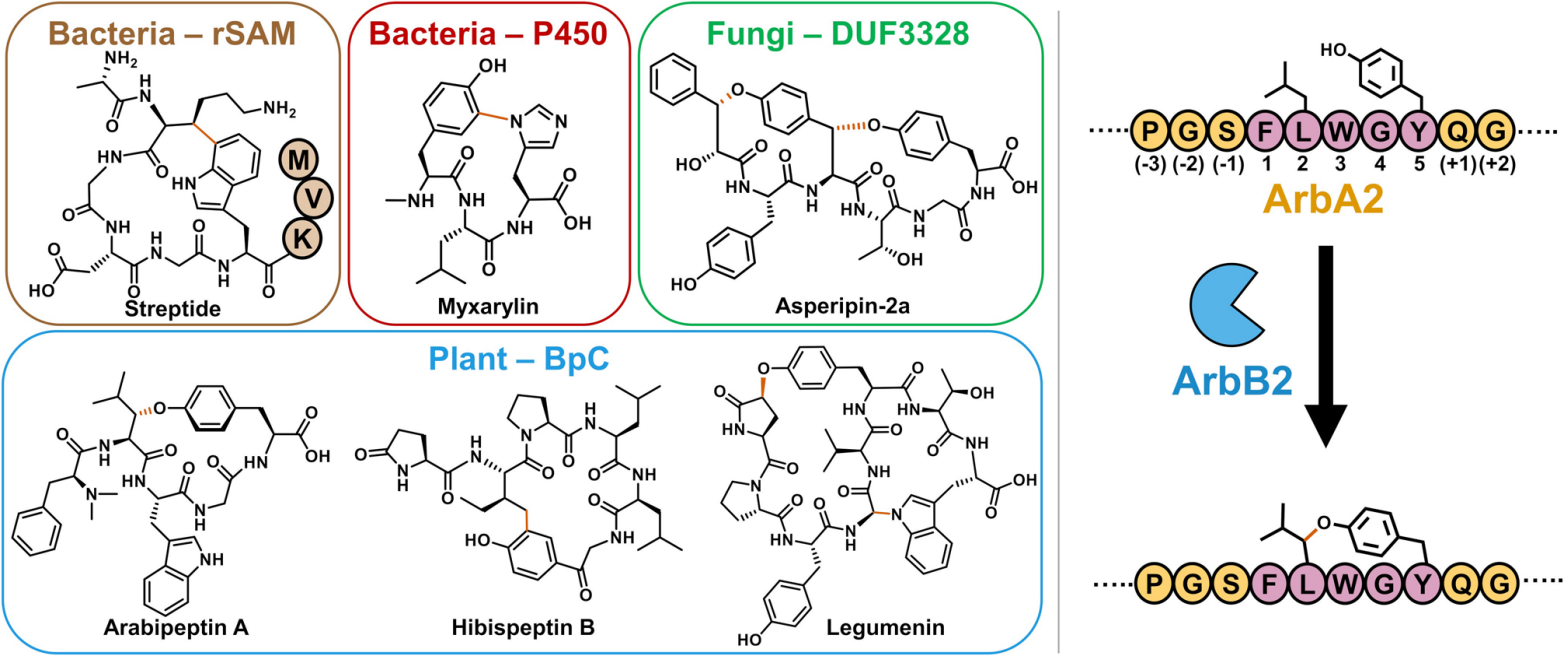
Side chain cross-linked RiPP natural products involving one or two redox active aromatic side chain(s). (Left) Example peptides, source, and biosynthetic enzyme class show the diversity of cross-linking reactions in biology. The installed side chain cross-links are indicated in vermillion. (Right) ArbB2 catalyzes installation of Tyr-O-Leu cross-link in the ArbA2 precursor peptide substrate. Standard peptide numbering scheme of the precursor peptides relative to the core (purple) is indicated.

BpCs represent an entirely new family of aromatic side chain cross-linking enzymes that were recently discovered in plants. This protein family is responsible for the cyclization of peptides forming the burpitide class of natural products and can install C–C, C–O, or C–N bonds between a redox active aromatic amino acid side chain and another amino acid.^21,23,25,34^ In contrast to P450s and rSAMs, which employ iron-containing cofactors, BpCs are copper-dependent. This feature is particularly intriguing because, although copper is the second most abundant redox active metal in biology, no copper enzymes were previously known to natively catalyze any type of peptide cyclization reaction. As a diverse class of metalloproteins, copper proteins can be categorized into several distinct types based on their ligand environments, spectroscopic features, and functional roles.^35^ A recent crystal structure of the BpC AhyBURP indicated the presence of four colocalized conserved histidine residues, likely responsible for coordinating two type 2 (T2) copper centers at the heart of this enzyme family.^25^

While all burpitides are defined by the function of a BpC, the actual cross-link they form can vary to create four known subclasses.^34^ For example, the lyciumin-type peptides are defined by a Trp-indole-N to carbon bond, while the cyclopeptide alkaloids are characterized by a Tyr-phenol-O to carbon cross-link (Figure 1). Additionally, the burpitide biosynthetic pathways have been found to exist in two parallel, but distinct types. In the first, the BpC is produced as a single polypeptide joined to its precursor peptide to create an autocatalytic enzyme (fused pathway).^21^ This system has been shown to be operational in the formation of diverse burpitides including legumenin (AhyBURP),^21,25^ moroidin (KjaBURP/BtoBURP),^22^ selanine A (SkrBURP),^21^ and nanmin.^26^ In contrast, other burpitides are produced from split pathways with distinct BpCs and precursor peptide genes.^34^ We have previously demonstrated the function of the split system for the biosynthesis of the cyclopeptide alkaloid arabipeptin A by ArbB2 ^23^ and implicated its presence in hibispeptins and moroidin (Figure 1).^23,36^

Despite their near ubiquity in eudicot and monocot plants, little is known about the exact chemistry, catalytic mechanism, and substrate recognition determinants for BpCs. These questions are made even more intriguing due to the unprecedented nature of the copper chemistry employed by this family of enzymes and the active site’s precise control over cross-linking in precursor peptides. This gap underscores the need to understand the chemistry involved and develop a catalytic system that can help elucidate the underlying mechanism.

Therefore, in this report, we describe the biochemical characterization of the split BpC ArbB2 and establish a functional, multiturnover catalytic system for a BpC for the first time. We demonstrate the O_2_-dependence and the necessity of an appropriate redox partner for a fully catalytic process. Using modeling, we successfully design minimal substrates that obviate the need for large 81-amino acid precursor peptides previously demonstrated for stochiometric activity.^23^ We then quantitatively explore the substrate scope of ArbB2 and demonstrate which binding interactions are important in the recognition sequence of the precursor peptide substrate. We extend this analysis to multicore peptide systems, showing that ArbB2 can process multiple cores on a single peptide substrate with a partially defined order. Finally, we illustrate that our biochemical insights into ArbB2 can be applied to other split BpCs to design simple substrates and achieve catalytic activity.

## Results and Discussion

### Modeling

Before beginning a detailed study of ArbB2’s reactivity, we first sought to identify a robust system for study. ArbB2 natively uses the precursor peptide ArbA2 as its substrate. ArbA2 is 162 amino acids in length and is composed of a signal sequence, N-terminal extension peptide, and three repeats of a recognition sequence responsible for protein binding and core peptide that matures to the final natural product. This is a highly complex substrate that would greatly complicate production and the in vitro study. Even trimming ArbA2 to a simplified 81-amino acid peptide as we had shown previously^23^ would create challenges for quantitative analysis. To address this, we used AlphaFold 3-based modeling^37^ to predict the key interactions between ArbB2 and ArbA2 to design minimal peptide substrates. As our focus is on the interactions between the precursor peptide and BpC in the split system, which are well suited for catalytic activity, the recently published structure of the autocatalytic AhyBURP^25^ with a fused substrate offered only minimal insight and modeling was necessary.

We first modeled the previously studied ArbB2_75–320_ construct in both its Apo and Holo forms, with the latter containing two copper ions (Figures 2A and S1). The overall fold of ArbB2 closely resembled the recently solved structure of AhyBURP (RMSD 2.6 Å over 160 of 246 C_α_, PDB: 8SY2),^25^ providing reasonable confidence in the model. Additionally, the copper ions were coordinated by the four conserved histidines, which generally aligned with the observations from the AhyBURP crystal structure. We recently recommended key structural assignments for the conserved features of BpCs to facilitate related discussions on reactivity and mechanisms. For instance, the histidine pairs are designated as HH-**A** and HH**-B** based on their relative positions in the sequence (vide infra).^24^

**Figure 2:**
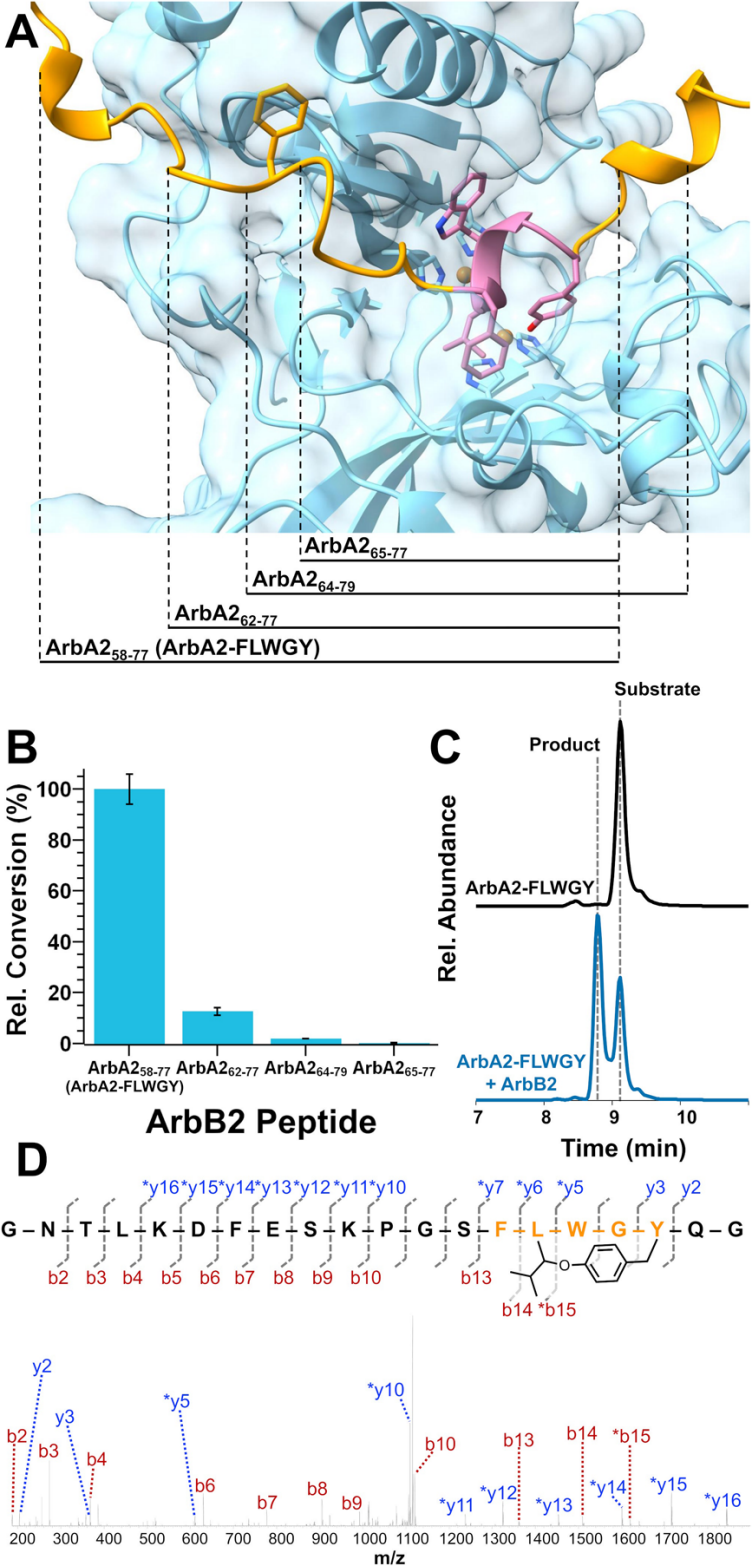
Model-guided design of minimal precursor peptides. (A) AlphaFold 3 model of ArbB2_75–320_ (blue) in complex with ArbA2_**23–150**_ (orange and coral). (B) ArbB2 shows substantial modification of ArbA2_**58–77**_ (ArbA2-FLWGY) and low levels of product with ArbA2_**62–77**_ and ArbA2_**64–79**_ substrates. (C) UHPLC-HRMS analysis of single turnover ArbB2 assays. Traces represent EICs for both the ArbA2_**58–77**_ substrate and cyclized product. (D) MS2 fragmentation of ArbA2_**58–77**_ (ArbA2-FLWGY) product supports cyclization at Tyr5 and Leu2. Error bars are the standard deviation of three trials.

As the substrate for ArbB2 is a peptide, we next modeled the ArbB2|ArbA2 complex to investigate the potential binding interactions. This approach has been previously applied in RiPP systems and has been shown to provide valuable insights.^38^ We first attempted the full length ArbA2 without the signal sequence but found the repeating core peptide units overlapped in the active site (Figure S2). To resolve this, we tested different truncations of ArbA2, modeling versions with lengths of 79, 45, and 30 amino acids. In each case, the F-L-W-G-Y core peptide fits into the active site, and a hydrophobic pocket in ArbB2 accommodated Phe­(-7), a phenylalanine in the recognition sequence located seven amino acids N-terminal to the core peptide (Figures 2A and S2). In the 79 and 45 amino acid ArbA2 peptide models, much of the N- and C-termini appeared to lack clear interaction with ArbB2. In contrast, most of the ArbA2 30mer appeared to associate with ArbB2 (Figure S2). Specifically, 15 amino acids of the recognition sequence N-terminal to the core peptide bound to ArbB2, while few clear interactions were evident C-terminal to the core peptide (Figure 2A). Ultimately, these results suggested that only a single combination of the repeating recognition sequence and core peptide may be needed for enzyme activity

### Identification of a Short Model Substrate

Based on AlphaFold 3 modeling, it appeared that much of the precursor peptide is dispensable. This would be advantageous as it allows for the construction of shorter substrates, which can be more readily produced and quantitatively analyzed compared with the full-length 162-amino acid ArbA2. Using the model as a guide, we designed three substrates of different sizes or core locations termed ArbA2_**58–77**_, ArbA2_**62–77**_, ArbA2_**64–79**_, and ArbA2_**65–77**_ (Figure 2A and Table 1).

**Table 1:** Peptide Substrate Sequences

Peptide Name	Sequence
ArbA2_65–77_	ESKPGSFLWGYQG
ArbA2_62–77_	KDFESKPGSFLWGYQG
ArbA2_64–79_	FESKPGSFLWGYQGND
ArbA2_58–77_ (ArbA2-FLWGY)	GNTLKDFESKPGSFLWGYQG
ArbA2-FLWGY F(−7)A	GNTLKDAESKPGSFLWGYQG
ArbA2-FLWGY S(−1)A	GNTLKDFESKPGAFLWGYQG
ArbA2-FLWGY Q(+1)A	GNTLKDFESKPGSFLWGYAG
ArbA2-FLWGY P(−3)L	GNTLKDFESKLGSFLWGYQG
CamA1-ILLY_113–132_	EGELKDFSVDPSGNILLYHG
CamA1-FFFY_20–40_	EGELKDFSVDPSGNFFFYHN
CamA2-ILWY_56–75_	GRVAKDISVDPSGNILWYHG

ArbB2_75–320_ was then expressed as a N-terminal Maltose Binding Protein (MBP)-fusion construct in Escherichia coli, purified, and refolded (see Supporting Information). Size exclusion chromatography showed that the MBP-ArbB2_75–320_ construct (referred to hereafter simply as ArbB2) was present as a monomer (Figure S3). To confirm that this monomeric state was also observed in free BpCs not fused to MBP, we used Tobacco Etch Virus (TEV) protease to cleave another BpC, CamB1 (vide infra), as ArbB2 had an internal TEV cut site. Analysis by size exclusion chromatography showed that CamB1 maintained a monomeric state after cleavage (Figure S3). Finally, modeling these split BpCs as either a monomer or a dimer did not alter their fold (Figure S3). These results contrast with the homodimeric state of the fused BpC AhyBURP.^25^ The purified ArbB2 was assayed in vitro, and Ultrahigh Performance Liquid Chromatography-High Resolution Mass Spectrometry (UHPLC-HRMS) analysis indicated product formation with ArbA2_**58–77**_, ArbA2_**62–77**_, and ArbA2_**64–79**_ (Figure 2B,C). Of these, ArbA2_**58–77**_ was clearly the best substrate at 78 ± 16% conversion in a 1:1 stoichiometric assay. MS2 fragmentation confirmed the location of cyclization to be between Tyr4 and Leu2 of the core peptide (Figures 2D, S4, and S5). Critically, chromatographic separation of the substrate and product allowed for quantitative deamination of product formation, as the isotopic mass envelope of the substrate and product overlap. In contrast, ArbA2_**65–77**_ was not accepted as a substrate as no product was observed (Figure 2B). Interestingly, ArbA2_**62–77**_ demonstrated more conversion than did ArbA2_**64–79**_ (Figure 2B). This suggests that the N-terminal extension found in ArbA2_**62–77**_ plays a more significant role in the binding and activity than the C-terminal extension of ArbA2_**64–79**_, aligning with the AlphaFold 3 model (Figure 2A). Ultimately, given these results and no clear improvement in turnover with longer substrates (Supplementary Note), ArbA2_**58–77**_ was selected for all subsequent assays and named ArbA2-FLWGY.

### Copper- and Dioxygen-Dependence

Previous studies have demonstrated that BpCs are copper-dependent^21^ and the first crystal structure of a BpC confirms that one copper ion is coordinated by the conserved histidine pairs of the HH-**B** side of the active site,^25^ while the HH-**A** side remains unoccupied.^24^ However, the active form of the copper within the active site is somewhat ambiguous.^25^ To begin establishing the redox chemistry involved, stoichiometric assays with a 1:1 enzyme to substrate ratio were conducted in the presence of excess Cu­(II) or Cu­(I) ions (Figure 3A). These assays were analyzed at different time points to compare the initial conversion rates between the reactions using each copper species. While control reactions lacking added copper showed no product formation, the assays supplemented with Cu­(I) consistently exhibited significantly higher conversion than those with Cu­(II), suggesting that Cu­(I) is the catalytically active species. This emphasizes the crucial role of electrons, or reducing equivalents, in facilitating the oxidative peptide cross-linking activity of BpCs.

**Figure 3:**
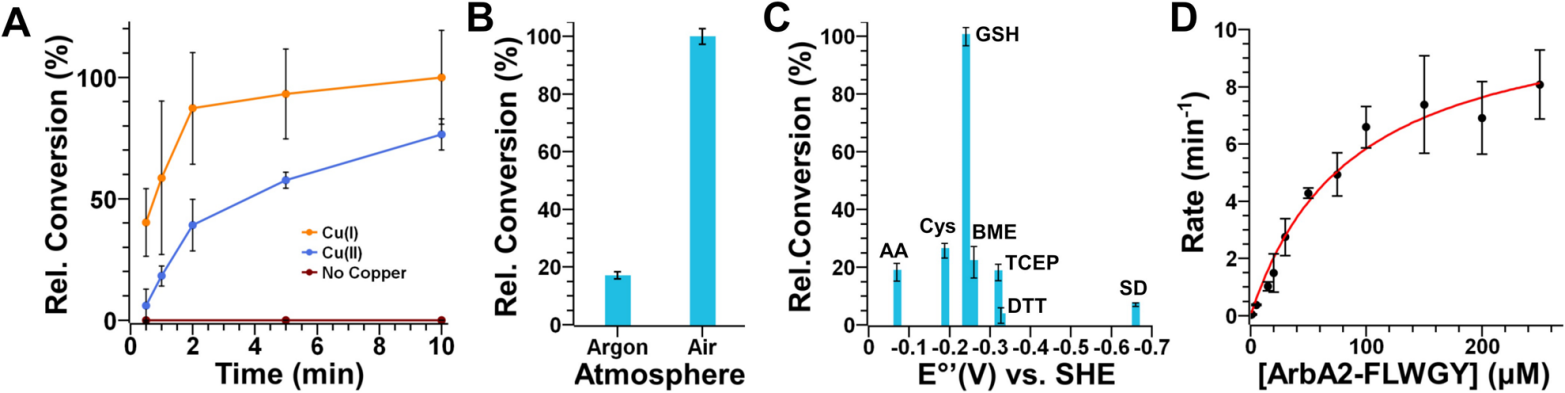
Catalytic competence for ArbB2. (A) Rate of cyclization of ArbA2-FLWGY by ArbB2 without the addition of copper or using an initial Cu­(I) or Cu­(II) cofactor. (B) Relative levels of ArbA2-FLWGY cyclization in low (argon) and ambient (air) oxygen atmospheres. (C) Reducing agent dependence on enzyme turnover. Note that the reduction potential values are listed vs standard hydrogen electrode (SHE) at pH = 7 and the assays were performed at pH = 8. (D) Michaelis–Menten kinetic analysis of ArbB2. Error bars indicate the standard deviation of three trials.

It has been shown that O_2_ is an essential substrate for AhyBURP.^25^ To verify whether O_2_ is required for the split BpC system, ArbA2-FLWGY was reacted with ArbB2 in the presence of Cu­(II) ions under both aerobic and anaerobic (argon) atmospheres. The substantial drop in the activity under an argon atmosphere confirms that the oxidized organic compound O_2_ indeed serves as a terminal electron acceptor for the ArbB2-catalyzed reaction (Figure 3B).

### Reductants Enable Multiple Turnovers

The improved reactivity of Cu­(I) suggested that the reduction of Cu­(II) to Cu­(I) is an essential step in the ArbB2 reaction mechanism and that an external source of electrons is required. This is supported by previous studies on the fused BpC AhyBURP that indicated that enzymatic activity can be improved with the addition of reducing agents.^25^ Therefore, we explored the reactivity of ArbB2 in a 150-fold excess of ArbA2-FLWGY substrate using seven different reductants with a wide range of reducing power, i.e., reduction potentials (from weakest to strongest): ascorbic acid (AA),^39,40^ cysteine (Cys),^41^ glutathione (GSH),^42^ dithiothreitol (DTT),^43,44^ 2-mercaptoethanol (BME),^45^ tris­(2-carboxyethyl)­phosphine (TCEP),^46^ and sodium dithionite (SD).^47^ Under the tested 150-fold excess ArbA2-FLWGY multiturnover in vitro conditions, glutathione enabled the most robust turnover with 75% conversion (∼110 turnovers, Figure 3C). Ascorbic acid, cysteine, BME, and TCEP also improved the levels of conversion but to a lesser degree. Finally, DTT and SD were the poorest reducing partners for the reaction. The apparent selectivity for glutathione is intriguing as this naturally occurring reductant is known to be present throughout plant cells at high concentrations (low mM),^48^ consistent with the mixed cellular localization of BURP-domain containing proteins in plants.^49^ Notably, these results contrast with those observed for the fused AhyBURP BpC which saw minimal reductant selectivity when catalyzing cyclization of the single fused core (a maximum of two turnovers^25^ vs 110 for ArbB2).

A closer examination of the reduction potentials of the redox partners and catalytic activity suggests a preference for a specific redox partner, neither too weak nor too strong (Figure 3C). This reproducible trend could reflect a mechanistic sensitivity to the redox environment, potentially influencing the BpC activity. Although preliminary, this idea is consistent with our recent commentary on the possible roles of redox conditions in BpC catalysis and warrants further investigation.^24^

This dependence on an external source of electrons also provides insight into the reaction scheme for BpCs.^24^ Copper enzymes are known to carry out various functions, including dioxygen binding, activation, and partial (2 e̅) or complete (4 e̅) reduction.^35^ The partial reduction typically produces hydrogen peroxide (H_2_O_2_), while the complete reduction yields water (H_2_O). To determine which process occurs in BpCs, we conducted semiquantitative H_2_O_2_ analysis of the catalytic assay over time. No H_2_O_2_ was detected during any stage of the multiturnover catalytic two-electron oxidative cyclization of ArbA2-FLWGY, which forms a C–O bond. This suggests that O_2_ is fully reduced to water in the reaction and is consistent with results from the fused BpC AhyBURP (Figure S6).^25^

### Kinetics and Total Turnover Number

Investigations into the steady-state kinetics of ArbB2 with the short substrate peptide ArbA2-FLWGY peptide revealed a *K*_m_ of 88.4 μM ± 16.9 and *k*_cat app_ of 11.0 min^–1^ ± 0.9 (Figure 3D). These values are comparable to that of the P450 OxyB, which has a *k*_cat app_ of 6 min^–1^ and a *K*_m_ ranging from 4 to 13 μM,^50^ despite ArbB2 catalyzing the more chemically challenging cross-linking of aromatic and aliphatic amino acids. In contrast, ArbB2 appeared to have substantially higher kinetic rates (*k*_cat app_) than rSAMs that conduct amino acid side chain cross-linking in RiPPs such as StrB (0.14 min^–1^),^17^ AgaB (0.12 min^–1^),^51^ and SuiB (0.18 min^–1^).^51^ StrB had a *K*_m_ of 160 μM,^17^ comparable to the 88.4 μM we observed for ArbB2. To complement the kinetic analysis, total turnover numbers (TTNs) of ArbB2 were examined using ArbA2-FLWGY at elevated substrate concentrations. Using 300-fold excess of ArbA2-FLWGY and an overnight incubation, we found a TTN of 175.06 ± 3.78 (see Supporting Information). While we were unable to find explicit TTNs for other enzymes that catalyze RiPP side chain cross-linking involving aromatic residues, we could extrapolate approximate in vitro TTN values using reported enzyme assay conditions. Using this estimation, P450_Blt_ has a TTN of ∼21 (∼85% conversion overnight)^8,52^ and the rSAM SuiB has a TTN of ∼ 26 (∼65% conversion in 1 h).^51^ While these are imperfect estimates, they suggest that BpCs, such as ArbB2, likely exhibit TTNs at least comparable to other enzyme families responsible for similar chemistry.

It should be noted that while activity for our ArbB2 construct appeared to be improved at a higher pH of 8.0 (Figure S7), the kinetic analysis were performed at pH 6.0 in an attempt to mimic the more physiologically relevant pH of the vacuole^53^ which is where some BpCs have been shown to be expressed.^54^ Reaction rates for the time points tested were also compared at pH 6.0 and 8.0 and shown to be minimally different (Figure S8).

### Mutants

A series of ArbB2 variants were generated to confirm the importance of the proposed active site residues. BpCs contain four invariant histidine residues that are responsible for copper coordination. Based on the AhyBURP structure and the holo ArbB2 AlphaFold 3 model, His237 and His249 (the HH-**A** pair) are expected to coordinate one copper ion, while His277 and His304 (the HH-**B** pair) coordinate the other (Figure 4A). As anticipated, the His237Ala, His249Ala, and His304Ala ArbB2 mutations all resulted in completely inactive variants, confirming their critical roles in copper binding and catalysis (Figure 4B). Likewise, His237Gln, His249Gln, and His304Gln ArbB2 variants were also catalytically inactive (Figure S9). Notably, the His277Ala mutant did not express, suggesting a critical stability or folding role for this residue.

**Figure 4:**
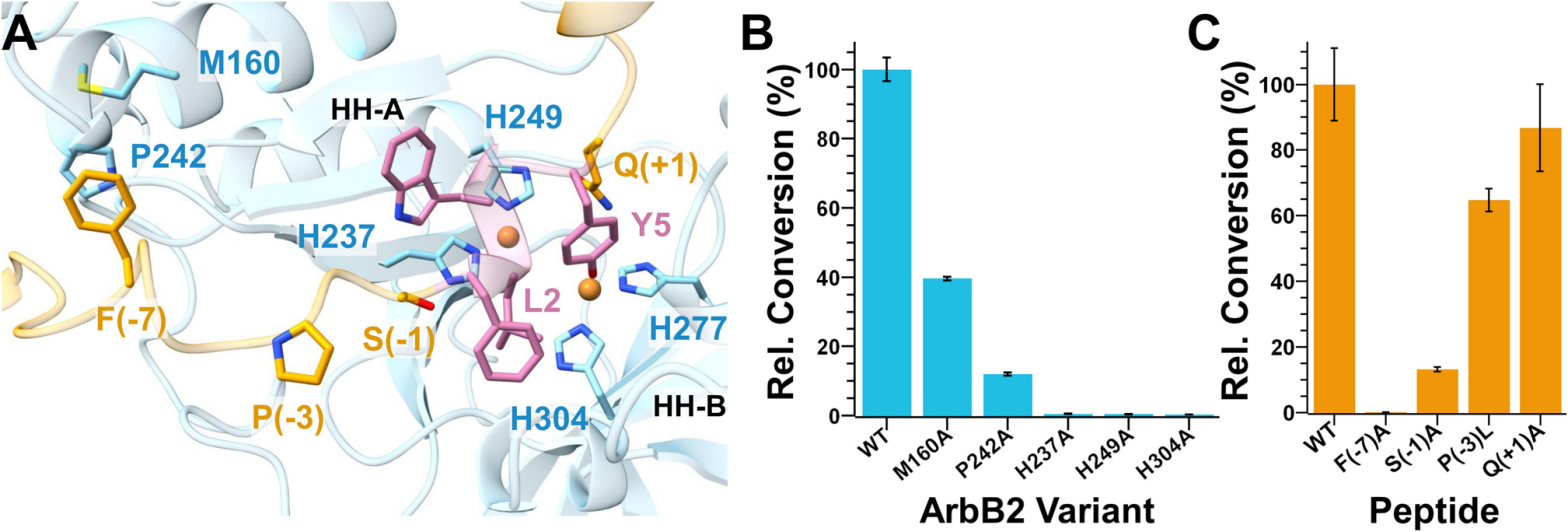
Impact of amino acid substitution on ArbB2 activity. (A) Model of holo ArbB2 (blue) in complex with ArbA2-FLWGY (orange and pink). (B) Relative conversion of ArbA2-FLWGY to product by ArbB2 and variants. (C) Relative conversion of ArbA2-FLWGY and variants to product by ArbB2. Error bars indicate the standard deviation of three trials.

We next explored the ArbB2|ArbA2-FLWGY binding interactions predicted by AlphaFold 3. A highly conserved Phe­(-7) of ArbA2 flanked by charged residues was observed to fit into a hydrophobic pocket in ArbB2 (Figure 4A and Table 1). Notably, this feature of a Phe surrounded by charged amino acids in the recognition sequence of the precursor peptide appears to be widespread in split burpitide biosynthetic pathways.^36^ Mutation of two amino acids in this pocket, Met160 and Pro242, to Ala showed a loss of ∼60 and 90% activity, respectively (Figure 4B). To directly confirm the necessity of the conserved phenylalanine in the ArbA2 peptide, Phe­(-7) was mutated to an alanine, resulting in a complete loss of activity (Figure 4C). Together, these experiments, along with sequence conservation, support the prominence of this binding determinant in both ArbB2 and other split BpCs.

Residues immediately N-terminal to the core also appeared to be significant based on the modeling. Ser­(-1) may form a hydrogen bond with the backbone amide of the core, helping to form the helical shape (Figure 4A). Pro­(-3) is also predicted to assist with positioning by allowing the core to fit through a pinch point at the front of the active site. Notably, Ser­(-1) is highly conserved in precursor peptides of C. arabica, while Pro­(-3) is more variable (Figure S10). To explore their significance, Ser­(-1) and Pro­(-3) were mutated. Ser­(-1)­Ala mutation resulted in a substantial loss of activity, whereas the Pro­(-3)­Leu mutant retained ∼65% of the activity of the native ArbA2-FLWGY (Figure 4C). Finally, we explored the necessity of the C-terminal residues. Gln­(+1) was mutated to Ala which resulted in a modest loss in activity, suggesting the C-terminus plays a minimal role in peptide binding (Figure 4C).

### Cyclization of Alternative Arabipeptins

While experiments up to this point have focused on the ArbA2 substrate and arabipeptin A’s F-L-W-G-Y core peptide sequence, we have previously shown C. arabica produces at least five distinct cyclopeptide alkaloids.^23^ Moreover, seven additional stand-alone precursor peptides are present in the genome and encode seven unique core sequences (Figure S11). Despite this diversity, the precursor peptides from only one of the three genetic loci are genomically clustered with BURP-domain containing proteins. Therefore, we sought to explore if these BpCs can catalyze cyclization of these diverse core peptide sequences.

Within the *arb* biosynthetic gene cluster are five precursor peptides representing two unique cores and three BpCs (ArbB1-3, Figure S11). Notably, ArbB1 appeared to be nonfunctional, as it was C-terminally truncated and lacked all four of the conserved active site CH motifs. Outside the *arb* cluster are two additional precursor peptides that contain six core sequences. To determine if ArbB2 could cyclize these alternative core sequences, 20 amino acid synthetic peptides were designed containing the observed core sequences of FLILY, VLILY, and LLLY. In each case, the native recognition sequence was used, which varied slightly from peptide to peptide (Figure 5).

**Figure 5:**
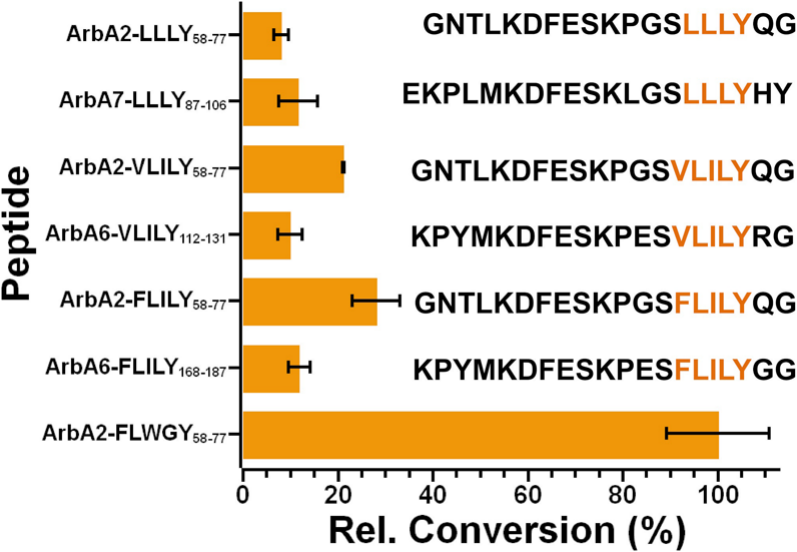
Cyclization of the alternative core sequences. Conversion levels were normalized to the ArbA2-FLWGY levels. Peptide amino acid sequences are listed. All ArbA2 sequences used the standardized ArbA2_58–77_ length. Error bars indicate the standard deviation of three trials.

Activity assays with ArbB2 showed cyclization of each precursor peptide, indicating that ArbB2 may be involved in the maturation of multiple cyclopeptide alkaloids. Under the assay conditions, ArbA2-FLWGY was the preferred substrate, whereas ArbA6-FLILY_168–187_, ArbA6-VLILY_112–131_, and ArbA7-LLLY_87–106_ showed similar activity with a relative conversion of ∼15–30% for each peptide (Figure 5). In particular, the ArbA2-LLLY_58–77_ product was noteworthy as this peptide had only a four amino acid core and fragmentation supported cyclization between the Y4 and L2 residues (Figures S12 and S13). This contrasts with the five amino acid cores and cross-linking at the 2 and 5 positions (Figures S14–S17). To evaluate if these differences were simply due to changes in the recognition sequence, we created a new set of peptides, all with the ArbA2-FLWGY recognition sequence. The ArbA2-FLILY_58–77_ and ArbA2-VLILY_58–77_ non-natural peptides demonstrated a modest increase in conversion compared with the native peptide, suggesting that small changes in the recognition sequence may impact cyclization efficiency (Figures 5 and S18–S23).

We next evaluated ArbB3, which is the other BpC in the *arb* locus. Unsurprisingly, ArbB3 also closely resembles the crystal structure of AhyBURP (RMSD of 2.7 Å, over 163 of 288 C_α_) as ArbB3 and ArbB2 share a high level of sequence identity at 93%. AlphaFold 3 modeling showed that amino acid substitutions were concentrated near the active site (Figure S24). To evaluate whether ArbB3 was functional or had differing substrate preferences, the ArbB3_23–288_ truncation was expressed and purified as an MBP fusion in the same manner as ArbB2. In vitro activity assays indicated that ArbB3 could cyclize ArbA2-FLWGY, ArbA6-FLILY_168–187_, ArbA6-VLILY_112–131_, and ArbA7-LLLY_87–106_ precursor peptides. Under these conditions, ArbB3 had a substrate selectivity similar to that of ArbB2, preferentially modifying ArbA2-FLWGY (Figure S25).

Finally, we explored ArbB2’s ability to modify non-native core sequences. All the bioinformatically and analytically^23^ observed core sequences indicate a cross-link between a Leu and Tyr residue. To test the ability of ArbB2 to catalyze a cyclization between a Tyr and other residues, peptides containing an Ile (F-I-W-G-Y core) or Val (F-V-W-G-Y core) in place of the Leu were synthesized and tested as substrates for ArbB2. Analysis of the assays by UHPLC-HRMS indicated the formation of products in each case but at a lower level than the native F-L-W-G-Y core sequence (Figure S26). MS2 fragmentation of the products suggested cyclization in some cases, but the nature and location of the putative cross-linkages are unknown (Figures S27–S34). Notably, these non-native cores produced two distinct product peaks with identical *m*/*z* values, some of which were consistent with the formation of the predicted macrocycle by MS2 analysis.

### Multicore Peptide Modification and Selectivity

A precursor peptide with multiple cores is a common feature in eukaryotic RiPP precursor peptides.^19,33,55–57^ Following this trend, multicore precursor peptides dominate both fused and split burpitide biosynthetic pathways.^21–23^ For example, C. arabica’s arabipeptin A sequence maps to ArbA2 and ArbA1 which contain three and five repeats of the FLWGY core, respectively. As only single core modification has been examined for both fused and split BpCs thus far, we sought to determine if ArbB2 can fully process a multiple core peptide and evaluate if there is any selectivity in modification order. Therefore, we heterologously expressed and purified a three-core version of ArbA2 that lacked the signal sequence and 12 amino acids at C-terminal portion (ArbA2_23–150_). In vitro assays were initiated with a 1:1 molar ratio of ArbB2 and ArbA2 (10 μM) and no reducing agent to prevent more than one turnover. Stepwise addition of glutathione to the reaction mixture followed by digestion with trypsin produced three distinct peptides, each corresponding to one core (Figure 6). This allowed us to track the formation of the cross-links in each core separately using UHPLC-HRMS. Our results showed that ArbB2 was indeed able to fully modify each of the three cores in ArbA2. Notably, there was also a clear and consistent preference for the order: core 2, followed by core 3, and finally core 1 (Figures 6 and S35). Whether this selectivity is due to minor variations in the recognition sequence, placement in the precursor peptide, or three-dimensional structure of the precursor peptide in solution remains to be explored.

**Figure 6:**
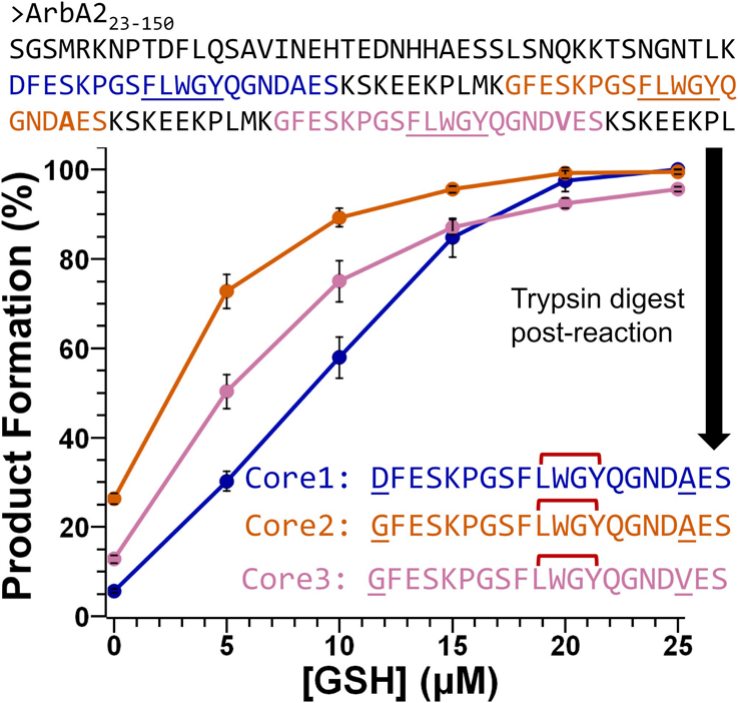
Processing of the multicore substrate. ArbB2 was reacted with ArbA2_23–150_ followed by the iterative addition of GSH. The reaction products were digested with trypsin to reveal three fragments by UHPLC-HRMS that mapped to core 1 (blue), core 2 (orange), and core 3 (coral). Error bars indicate the standard deviation of three trials. The sequence of ArbA2_23–150_ contains an N-terminal S-G-S remnant from the purification procedure.

### Split BpCs from Other Plants

Previous bioinformatic studies have indicated that split BpCs are responsible for diverse burpitides across many plants. Specifically, 89% of eudicot plants appear to have standalone BURP-domain containing proteins and are often genomically adjacent to putative precursor peptides.^23^ Therefore, to determine whether our biochemical insights extend beyond ArbB2 and ArbB3, we investigated putative BpCs from Ceanothus americanus. This plant is from the Rhamnaceae family and is phylogenetically distant from the Rubiaceae family of C. arabica. C. americanus is well known to produce cyclopeptide alkaloids such as frangulanine and homoamericine (Figure 7), but its molecules are structurally distinct from arabipeptin A in that they are oxidatively decarboxylated.^58,59^ As no genome is present for C. americanus, we searched the transcriptomes for BURP-domain containing proteins using the publicly available HMM (PF03181). This process identified 31 putative cyclases that were filtered by the presence of four conserved CH pairs. These cyclases were then modeled with three putative substrates identified in the C. americanus transcriptome whose cores correspond to known cyclopeptide alkaloids: ILLY (frangulanine), ILWY (homoamericine), and FFFY (metabolomically observed CAM603).^23,36^ Candidate cyclases that did not have a binding interaction conducive to turnover were filtered out.

**Figure 7:**
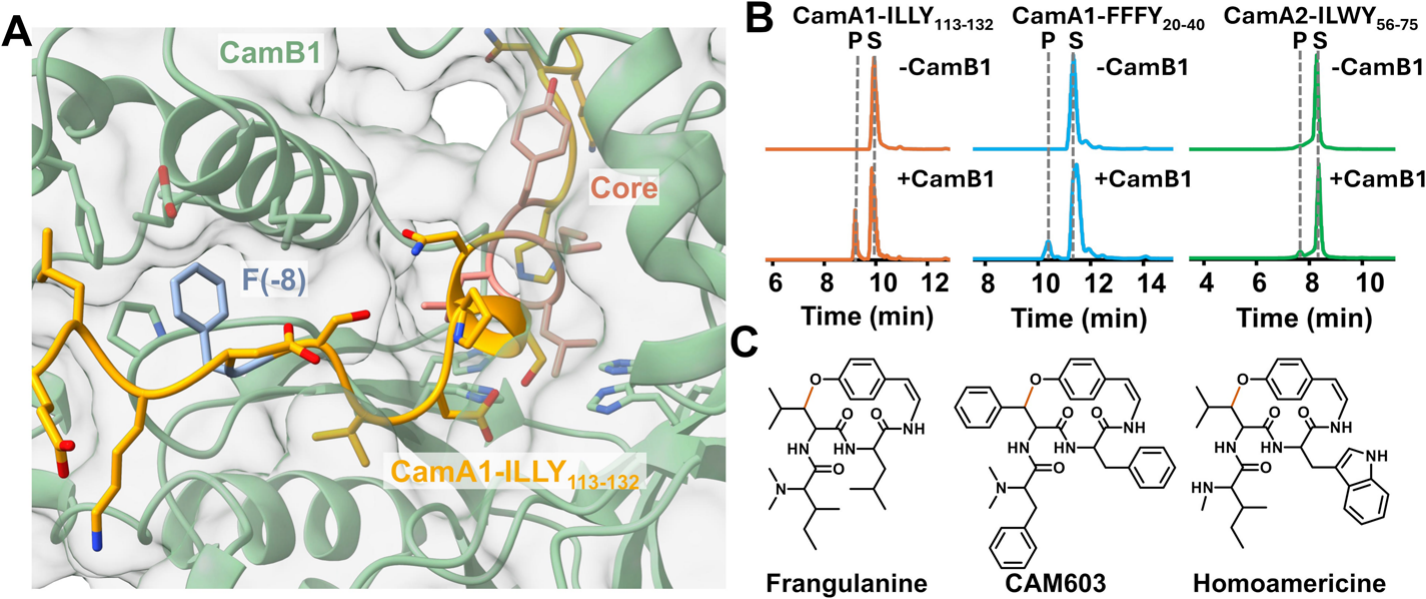
Reconstitution of CamB1 activity. (A) AlphaFold 3 model of CamB1 (mint green) in complex with CamA1-ILLY_113–132_ (orange and coral) shows core positioning near active site and conserved Phe (blue) fitting into a hydrophobic pocket of CamB1. (B) UHPLC-HRMS analysis of CamB1 assays shows modification of CamA1-ILLY_113–132_ (orange traces), CamA1-FFFY_20–40_ (blue traces), and CamA2-ILWY_56–75_ (green traces). Traces represent EICs for substrate and product. (C) Structures of the burpitides corresponding to each of the C. americanus core sequences with the bonds formed by CamB1 in vermillion.

This combination of multiple filtering steps led to the identification of putative BpC CamB1. Detailed examination of each of the three CamB1|CamA models showed apparent similarities to the C. arabica cyclases despite only 45% amino acid sequence identity. Additionally, the CamB1 model was structurally consistent with AhyBURP (RMSD of 2.0 Å over 156 of 256 C_α_). In each case, the core peptide sits in the four-His copper binding active site (Figures 7A and S36), suggesting that each of these native core sequences could serve as substrates for CamB1. As with ArbB2|ArbA2, a conserved hydrophobic amino acid flanked by charged residues in the precursor peptide fits into a hydrophobic binding pocket in CamB1 (Figures 7A and S36). Notably, the CamA2-ILWY_56–75_ peptide does not contain a phenylalanine at this position and instead an isoleucine residue binds to the pocket (Figure S36). The transcriptomic data of C. americanus indicate hydrophobic amino acids other than phenylalanine can bind to the pocket suggesting this example of an isoleucine binding within the pocket is a natural variant (Figure S37).

To validate this prediction, a CamB1 truncation lacking the signal sequence, CamB1_23–278_, was expressed as an MBP fusion in E. coli, purified, and refolded similarly to ArbB2. In vitro reconstitution indicated cyclization activity for each of the three 20 amino acid precursor peptides tested: CamA1-ILLY_113–132_, CamA1-FFFY_20–40_, and CamA2-ILWY_56–75_ (Figure 7B and Table 1). MS2 analysis supported cyclization between the conserved Tyr4 and second amino acid of the core peptide in each case (Figures S38–S43). Product quantitation indicated that the most preferred of the substrates for CamB1 was CamA1-ILLY_113–132_, followed by CamA1-FFFY_20–40_, and last CamA2-ILWY_56–75_ with 57, 34, and 14% conversion, respectively (Figure S44). These levels of conversion and the ability to cyclize multiple core peptide sequences mirror our observations for ArbB2.

## Summary and Conclusions

BpCs represent an intriguing new class of copper-dependent peptide cyclases. Although bioinformatically identified in 1998,^60^ their function has only recently been identified.^21–23,25^ To this point, only single-turnover activity has been reported with little information available concerning substrate recognition and scope.^21,23,25^ This report uses a combination of modeling, chemical, and biochemical studies to provide deeper insight into this unique enzyme family.

Despite no reported crystal structure of a BpC at the onset of this study, AlphaFold 3 effectively predicted the BURP-domain fold and identified testable binding interactions. Using this model, minimal substrates were successfully designed that reduce the 162 amino length of ArbA2 to 20 amino acids. This is notable as it suggests most of the precursor peptide of split BpCs is dispensable, and only a combination of recognition sequence and core peptide is essential for catalysis. Whether the leader-like N-terminal extension that proceeds with the first precursor peptide enhances peptide binding remains to be evaluated. Given that only single-turnover activity was observed, external electrons appeared to be necessary for catalytic turnover. Indeed, Cu­(I) was found to enhance the reaction rate over Cu­(II). Screening various reductants revealed a clear preference for glutathione, a physiologically relevant reductant found at millimolar concentrations in plant tissues. Turnover decreased as reductants deviated from glutathione’s reduction potential, suggesting that catalytic activity is optimized with a redox partner of moderate strength, neither too weak nor too strong. Combined with our verification of O_2_-dependence and absence of H_2_O_2_ production by these enzymes, a putative reaction scheme can be devised for BpCs wherein the oxidative cyclization of the Tyr and Leu in ArbA2 is coupled to a four-electron reduction of O_2_ to two water molecules-two electrons provided by an exogenous reductant and two from the substrate (eq 1).^24^ The mechanistic details of this reaction remain to be explored.


1

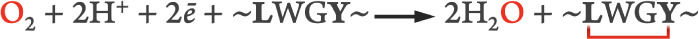



The successful catalytic reconstitution of ArbB2 enabled a detailed characterization of the biochemical properties of this enzyme. Kinetic analysis showed comparable or higher reaction rates relative to the unrelated but conceptually isofunctional rSAM and P450 enzyme families. Mutagenesis of both the enzyme and precursor peptide highlighted the importance of a hydrophobic pocket accommodating a Phe in the recognition sequence, as suggested by the AlphaFold 3 model. The prevalence of a single hydrophobic amino acid flanked by charged residues is widely present in recognition sequences of split BpC precursor peptides, indicating that this may be a nearly universal feature responsible for proper positioning of the core peptide into the active site. A similar feature is not apparent, and possibly unnecessary, in validated fused BpCs (Figure S1). Importantly, the biochemical methods developed to study ArbB2 extended effectively to both closely (ArbB3) and distantly (CamB1) related cyclopeptide alkaloid producing BpCs. We anticipate these approaches will facilitate study of other BpC family members, such as those responsible for side chain cross-links in hibispeptins and stephanotic acids. Together, these findings offer fundamental insights into the biochemical details of BpCs and provide a basis for future engineering and mechanistic studies of this new class of copper enzymes.

## Supplementary Material



## Notes

Experimental details on protein expression, protein purification, enzyme assays, analytical analysis, and supplementary figures, and tables (PDF)
