# Supplementary material for: Peptide Recognition Sequence Guides Catalytic Side Chain Cross-Linking of Plant Peptides by Copper-Dependent Cyclases
Source: J Am Chem Soc. 2025 Jun 5;147(24):20284–93. doi: 10.1021/jacs.4c15470 (PMC12186524; doi:10.1021/jacs.4c15470)
Supplement: Supplementary file 1 [file ja4c15470_si_001.pdf]

Peptide Recognition Sequence Guides Catalytic Side Chain Crosslinking of Plant Peptides by a Copper-Dependent Cyclase

Stella T. Lima,[‡] Michael A. Pasquale,[‡] M. Rafiul O. K. Noyon, Elizabeth A. Clark, Corinne R. Laws, Shabnam Hematian,[*] and Jonathan R. Chekan[*]

Department of Chemistry and Biochemistry, University of North Carolina at Greensboro, NC 27402, USA

[‡]Contributed Equally
*Corresponding Authors' Emails: s_hemati@uncg.edu (S.H.) and jrchekan@uncg.edu (J.R.C.)

Table of Contents

Data Deposition S03

Experimental Section
Peptides S03
Protein Modeling with AlphaFold3 .... S03
Protein Expression S03
Protein Purification S04
ArbA2 multicore precursor peptide S04
Gel filtration standard curve S05
In vitro enzymatic assays S05
Minimal substrate S05
Time course – Cu(II) S06
Copper and dioxygen dependence assays S06
Peroxide formation assays S06
pH S07
Reducing agents S07
General optimized assays S07
Total turnover S07
ArbB2 kinetics S07
ArbA2 multicore assays S07
UHPLC-HRMS method S08
Ionization Efficiency S08
Exact Mass of Peptides S08
Peptide Synthesis and Purification S09

DNA Sequences, Protein Sequences, Peptide Sequences, and Accession Numbers
Protein Sequences Used S09
Precursor peptides used in this study S12
Primers peptides used in this study S13
Protein Accession Numbers S14

Supplementary Figures
Figure S1 .... S15

Figure S2 ……………………………………………………………………………………S16
Figure S3 ……………………………………………………………………………………S17
Figure S4 ……………………………………………………………………………………S18
Figure S5 ……………………………………………………………………………………S19
Figure S6 ……………………………………………………………………………………S20
Figure S7 ……………………………………………………………………………………S21
Figure S8 ……………………………………………………………………………………S22
Figure S9 ……………………………………………………………………………………S23
Figure S10 ……………………………………………………………………………………S24
Figure S11 ……………………………………………………………………………………S25
Figure S12 ……………………………………………………………………………………S26
Figure S13 ……………………………………………………………………………………S27
Figure S14 ……………………………………………………………………………………S28
Figure S15 ……………………………………………………………………………………S29
Figure S16 ……………………………………………………………………………………S30
Figure S17 ……………………………………………………………………………………S31
Figure S18 ……………………………………………………………………………………S32
Figure S19 ……………………………………………………………………………………S33
Figure S20 ……………………………………………………………………………………S34
Figure S21 ……………………………………………………………………………………S35
Figure S22 ……………………………………………………………………………………S36
Figure S23 ……………………………………………………………………………………S37
Figure S24 ……………………………………………………………………………………S38
Figure S25 ……………………………………………………………………………………S39
Figure S26 ……………………………………………………………………………………S40
Figure S27 ……………………………………………………………………………………S41
Figure S28 ……………………………………………………………………………………S42
Figure S29 ……………………………………………………………………………………S43
Figure S30 ……………………………………………………………………………………S44
Figure S31 ……………………………………………………………………………………S45
Figure S32 ……………………………………………………………………………………S46
Figure S33 ……………………………………………………………………………………S47
Figure S34 ……………………………………………………………………………………S48
Figure S35 ……………………………………………………………………………………S49
Figure S36 ……………………………………………………………………………………S50
Figure S37 ……………………………………………………………………………………S51
Figure S38 ……………………………………………………………………………………S52
Figure S39 ……………………………………………………………………………………S53
Figure S40 ……………………………………………………………………………………S54
Figure S41 ……………………………………………………………………………………S55
Figure S42 ……………………………………………………………………………………S56
Figure S43 ……………………………………………………………………………………S57
Figure S44 ……………………………………………………………………………………S58
Figure S45 ……………………………………………………………………………………S59
Figure S46 ……………………………………………………………………………………S60
Figure S47 ……………………………………………………………………………………S61

Supplementary Note and Figure 48 ………………………………………………………S62

Supplementary References ……………………………………………………………S63

Data Deposition

Protein Name	Organism	NCBI Accession Numbers
CamB1	*Ceanothus americanus*	PV340619
CamA1	*Ceanothus americanus*	PV340617
CamA2	*Ceanothus americanus*	PV340616

Experimental Section

Peptides

The majority of the peptides were purchased from Biomatik (Kitchener - Ontario, Canada). C-terminally amidated ArbA2-FLWGY-NH2$_{58\text{-}77}$, ArbA2-FVWGY-NH2$_{58\text{-}77}$, and ArbA2-FIWGY-NH2$_{58\text{-}77}$ were synthesized as described in “Peptide Synthesis and Purification” section.

Protein Modeling with AlphaFold 3

AlphaFold 3[1] was accessed through its website interface. Protein sequences were submitted without including the signal peptide sequence, which had been pre-processed using SignalP 6.02. The sequences for BpCs examined in this project were entered as the primary input, alongside their precursor peptides. For cases where two copper ions were required, they were included in the third entry field.

Protein Expression

The ArbB2, ArbB3, and CamB1 amino acid sequences were analyzed to search for transmembrane sequences with TMHMM 2.0[2,3] and none were predicted. To predict signal peptide sequences, SignalP 5.0[4] was used. Detected signal peptides were excluded prior to cloning. The genes were synthesized codon optimized for expression in *E. coli* (Twist Bioscience, San Francisco, CA) and assembled into N-terminal hexahistidine tag (His$_6$) pET28-MBP (maltose binding protein) vector[5] using Gibson Assembly Master Mix (New England Biolabs). The vector was transformed into *E. coli* DH10B chemically competent cells for plasmid replication and were sequenced for confirmation (Plasmidsaurus). Plasmid was also transformed into *E. coli* BL21(DE3) for expression. The MBP-BpCs were expressed by inoculating 15 mL of LB media supplemented with 50 µg/mL kanamycin. These cultures were incubated overnight at 37 ºC at 200 rpm. The starter cultures were then used the next day to inoculate 1 L of Terrific Broth supplemented with 50 µg/mL kanamycin in a 2.8 L flask. This was incubated at 37 ºC at 200 rpm until an OD$_{600}$ of 0.8 was achieved the cultures were then cooled to 18 ºC. After an hour, isopropyl-β-D-thiogalactopyranoside (IPTG) was added with a final concentration of 1 mM to induce protein expression. The cultures were then incubated overnight at 18 ºC at 200 rpm. The cultures were harvested at 4,000 × *g* at 4 ºC for 1 hour. The pellet was resuspended in resuspension buffer (20 mM Tris Base pH 8.0, 500 mM NaCl, and 10% glycerol) for lysis and stored at -75 ºC until purification.

Protein Purification

The resuspended *E. coli* DE3 cells were lysed using a Branson 150 Sonifier Cell Disruptor, microtip, 80% amplitude, 15 s pulse on/45 s pulse off a total of 8 minutes. The lysed cells were then centrifuged at 15,000 × *g* for 30 minutes or until lysate appeared clear. The lysate containing the soluble protein was unfolded with 8 M urea for 1 hour with slow stirring. The denatured protein was purified using a Cytiva ÄKTAGo FPLC system at 4 ºC and all buffers used in the purification system had been filtered through 0.22 µm polyvinylidene difluoride membrane filters. The denatured protein was loaded onto a Cytiva 5-mL HisTrap HP column that had been equilibrated with Buffer A (20 mM imidazole, 20 mM Tris base pH 8.0, 0.5 M NaCl, and 8 M urea) at a flow rate of 2 mL/min. The column was then washed with Buffer A until the UV returned to the base line ~30 mL. The His_6-tagged MBP-BpCs were then eluted with Buffer B (250 mM imidazole, 20 mM Tris base pH 8.0, 0.5 M NaCl, and 8 M urea) with a linear gradient from 100% Buffer A to 100% Buffer B over 20 minutes (40 mL), collecting 5 mL fractions. The fractions were assessed using SDS-PAGE (12% acrylamide). Fractions that were ~90% pure were combined and diluted to <0.6 mg/mL. The diluted protein was then refolded using dialysis under reducing conditions in the following order: Buffer 1 (4 M urea, 50 mM Tris base pH 8.0, 250 mM NaCl, 10 mM BME, 10% glycerol, and 700 mM arginine) overnight, Buffer 2 (1 M urea, 50 mM Tris base pH 8.0, 250 mM NaCl, 10 mM BME, 10% glycerol, and 700 mM arginine) for 4 hours, Buffer 3 (50 mM Tris base pH 8.0, 250 mM NaCl, 10 mM BME, 10% glycerol, and 700 mM arginine) overnight. The refolded protein was then concentrated to ~2 mL using an Amicon Ultra-15 50 kDa spin concentrator. Protein was then applied to a Cytiva HiLoad 16/60 Superdex 200 size exclusion column pre-equilibrated with GF Buffer (20 mM citrate-phosphate pH 7.5, 300 mM NaCl, and 10% glycerol). Fractions (3 mL) containing non aggregated protein were collected and concentrated to ~2 mL using 50 kDa spin concentrators. They were loaded onto a pre-equilibrated HiLoad 16/60 Superdex 75 size exclusion column (GE Healthcare Life Sciences) with GF Buffer. Fractions (3 mL) were collected and assessed using SDS-PAGE (12% acrylamide). Fractions more than ~90% pure were collected and concentrated, and frozen. Protein aliquots were stored at 75 ºC until purification until use. Proteins concentration was assessed using a Qubit Protein BR Assay kit on a Qubit 4 Fluorometer (Invitrogen) and determined to be 174.4 µM for enzymatic assays. For all ArbB2 mutants the same method of purification was applied, and following concentration were obtained: H226A: 11.8 µM, H293A: 190 µM, H238A: 247.8 µM, M149A: 11.8 µM, P231A 29.6 µM. The MBP-$ArbB3_{23-288}$ was concentrated to 21.6 µM. The MBP-$CamB1_{23-278}$ was concentrated to 74.7 µM. See **Figures S45** and **S46** for SDS-PAGE of purified protein used in this study.

ArbA2 multicore precursor peptide

The $ArbA2_{23-150}$ multicore sequence was amplified by PCR from the ArbA2 sequence optimized for expression in *E. coli*, excluding the signal sequence predicted by SignalP 6.0[4]. The $ArbA2_{23-150}$ was assembled into pET28-MBP plasmid using Gibson Assembly Master Mix (New England Biolabs). Transformations in *E. coli* DH10B and BL21(DE3), for plasmid verification/replication and expression respectively, were carried out, and the assembled sequence was confirmed (Plasmidsaurus). The transformed *E. coli* BL21(DE3) cells containing the MBP-ArbA2 $_{23-150}$, excluding the signal sequence predicted by SignalP 6[4]. The $ArbA2_{23-150}$ was assembled into pET28-MBP plasmid using Gibson Assembly Master Mix (New England Biolabs). The transformed BL21(DE3) containing the MBP-ArbA2 $_{23-150}$ plasmid was incubated with 20 mL of LB medium containing 50 µg/mL kanamycin at 37 ºC and 200 rpm as a starter culture. The following day, this starter culture was used to inoculate 1 L of Terrific Broth media containing 50 µg/mL kanamycin in 2.8 L flasks, shaking at 37 ºC and 200 rpm until an OD_{600} of ~0.8 was reached. The

incubation temperature was then lowered to 18 ºC, and after 1 hour, isopropyl-β-D-thiogalactopyranoside (IPTG) was added to a final concentration of 1 mM to induce protein production. The culture was incubated at 18 ºC with shaking for approximately 4 hours at 200 rpm. The cells were subsequently harvested by centrifugation at 4000 × *g* and 4 ºC for 1 hour. The pellet was resuspended in lysis buffer (20 mM Tris Base pH 8.0, 500 mM NaCl, and 10 % glycerol) and cells containing MBP-ArbA2$_{23-150}$ was subjected to sonication on ice (Branson 150 Sonifier Cell Disruptor, microtip, 80% amplitude, with 15-second pulses on and 45-second pulses off for a total of 8 minutes). The resulting lysate was centrifuged at 15,000 × *g* for 40 minutes at 4 ºC. Protein purification was carried out at 4 ºC using an ÄKTAGo FPLC system (Cytiva). The clarified lysate was applied to a 5 mL HisTrap HP column (Cytiva), pre-equilibrated with Buffer A (30 mM imidazole, 20 mM Tris base, pH 8.0, and 1 M NaCl) at a flow rate of 2 mL/min. The column was washed with 30 mL of Buffer A. His$_6$-tagged MBP-ArbA2$_{23-150}$ was eluted using Buffer B (250 mM imidazole, 20 mM Tris base, pH 8.0, and 1 M NaCl) with a linear gradient from 100% Buffer A to 100% Buffer B over 40 mL, while collecting 5 mL fractions. Purity was analyzed by SDS-PAGE (12% acrylamide), and fractions with at least 90% purity were pooled together. MBP-ArbA2$_{23-150}$ was concentrated to approximately 2 mL using Amicon Ultra-15 10 kDa spin concentrators. The sample was then applied to a pre-equilibrated HiLoad 16/60 Superdex 75 size exclusion column (GE Healthcare Life Sciences) with GF Buffer (20 mM citrate-phosphate pH 7.5, 300 mM NaCl, and 10% glycerol), and purified fractions of 3 mL were collected. MBP-ArbA2$_{23-150}$ was concentrated to around 2 mL, and tobacco etch virus (TEV) protease was added for overnight incubation at 4 ºC, leaving some uncleaved protein as a control for verification of cleavage efficiency of MBP by SDS-PAGE (12% acrylamide). The cleaved ArbA2$_{23-150}$ precursor peptide was concentrated to 1140 µM, determined using Qubit Protein BR Assay kit on Qubit 4 Fluorometers (Invitrogen).

Gel filtration standard curve

The Bio-Rad Gel Filtration Standard mixture was used to generate the protein standard curve (**Figure S3**). 2 mL of mixture containing thyroglobulin (670,00 Da), γ-globulin (158,000 Da), ovalbumin (44,000 Da), myoglobin (17,000 Da), and vitamin B12 (1,350 Da), was inject to a Cytiva HiLoad 16/60 Superdex 200 or 75 size exclusion column pre-equilibrated with GF Buffer (20 mM citrate-phosphate pH 7.5, 300 mM NaCl, and 10% glycerol). The elution times were plotted as follows: log (molecular weight) versus the gel phase distribution coefficient (K_{av}). ($K_{av} = (V_e-V_o)/(V_t-V_o)$; Ve: analyte retention volume; V_o: void volume; V_t: total volume). MBP-ArbB2$_{75-320}$ was analyzed alone on a separate run using the same conditions and its elution time was used to estimate its molecular weight.

In vitro enzymatic assays

Single-Turnover with Minimal Substrate

ArbB2 single-turnover minimal substrate assays were conducted in 50 mM citrate-phosphate buffer at pH 7.5 with 1 mM $CuSO_4$, 10 µM enzyme, and 10 µM substrate at RT for 1 hour. Assays were quenched by adding two volumes of 75% acetonitrile with 0.1% formic acid. Samples were analyzed by Ultra-High Performance Liquid Chromatography-High Resolution Mass Spectrometry (UHPLC-HRMS).

Catalytic Assays with ArbA2-FLWGY

ArbB2 activity assay were performed in 50 mM of citrate-phosphate buffer (pH 6.0) adding 150 µM of linear peptide, 1 mM of $CuSO_4$, 500 µM of glutathione, and 5 µM of purified ArbB2 enzyme.

Total reaction volume was adjusted to 100 µL with citrate-phosphate buffer and incubated at RT. Aliquots of 15 µL were taken at 5, 10, 20, 30, 45, and 60 min, and quenched with two volumes of 75% acetonitrile with 0.1% formic acid. All assays were prepared in triplicate. The samples were then centrifuged at maximum speed (21,000 × *g*) for 10 min, and supernatants were collected for UPLC-HRMS analysis.

Stoichiometric Assays with Copper(I) or Copper(II)
To determine the active copper form, assays were conducted in 50 mM citrate-phosphate buffer at pH 7.5 with either 1 mM [tetrakis(acetonitrile)copper(I)] hexafluorophosphate, $[Cu^{I}(CH_3CN)_4]PF_6$ (for Cu(I)), or copper(II) sulfate, $CuSO_4$ (for Cu(II)). Each assay contained 10 µM enzyme and 10 µM ArbA2-FLWGY substrate in a 200 µL total volume, incubated at RT. The 25 mM Cu(I) stock solution was prepared in acetonitrile in a Vacuum Atmospheres OMNI-Lab inert atmosphere glovebox under nitrogen (<0.5 ppm of O_2 and H_2O) and transferred with a Hamilton gastight syringe. The 25 mM copper(II) stock solution was prepared in water. Fractions of 25 µL were taken at 0.5, 1, 2, 5, and 10 min and quenched with 50 µL of 75% MeCN 0.1% formic acid. Samples were then centrifuged at maximum speed (21,000 × *g*) for 10 min, and supernatants were collected for UPLC-HRMS analysis. Each assay was performed in triplicate.

Dioxygen Dependence
Dioxygen dependence assays were conducted in 50 mM citrate-phosphate buffer (pH 8.0) with 1 mM $CuSO_4$, 500 µM glutathione, 3 µM enzyme, and 150 µM ArbA2-FLWGY substrate. For anaerobic conditions, the assay mixtures, excluding the enzyme, were incubated at RT under a water-saturated argon flow for 20 min using a Schlenk line. The pipettes and tips were also purged with argon before use. Then enzyme was then added to initiate reactivity, and the assays were kept under argon for 2h. Each assay was performed in triplicate. Parallel assays under aerobic conditions were also conducted as controls. All reactions were quenched with two volumes of 75% acetonitrile containing 0.1% formic acid, centrifuged at 21,000 × *g* for 10 min, and the supernatants were collected for UPLC-MS analysis.

Peroxide formation assays
The formation of any hydrogen peroxide was analyzed by semiquantitative QUANTOFIX peroxide test strips. To ensure accurate detection of peroxide without interference, six standards with known H_2O_2 concentrations were prepared at 0, 0.5, 2, 5, 10, and 20 mg/L, corresponding to 0, 14.7 58.8 147, 294, and 588 µM, respectively. Each standard was prepared in a solution containing 50 mM citrate-phosphate buffer (pH 8.0), 1 mM $CuSO_4$, 500 µM glutathione, and 150 µM ArbA2-FLWGY substrate. Triplicates of these mixtures were made and tested for hydrogen peroxide concentration with the test strips. Different shades of blue color correspond to the concentration of hydrogen peroxide in mg/L (**Figure S6**). In **Figure S6**, the reference for the concentrations is given which is acquired from the box of the test strips. The catalytic assays were also performed with 50 mM citrate-phosphate buffer pH 8.0, 1 mM $CuSO_4$ with either 1 µM enzyme, and 150 µM substrate or 2 µM enzyme, and 300 µM incubated at RT for 5, 15, 30, 60, 120 min and tested for peroxide formation. Samples were also quenched with two volumes of 75% acetonitrile with 0.1% formic acid, centrifuged at maximum speed (21000 × *g*) for 10 min. The supernatants were collected and subjected to UHPLC-HRMS analysis, which showed conversion of substrate into product. The formation of >100-200 µM of cyclized product during catalysis was confirmed, revealing that no trace amounts of hydrogen peroxide were produced.

pH

To determine the optimal in vitro pH for ArbB2, 25 µL assays were performed in 50 mM citrate-phosphate buffer at pH 5.0, 5.5, 6.0, 6.5, 7.0, 7.5 or 8.0, 150 µM of ArbA2-FLWGY, 1 mM $CuSO_4$, 500 µM GSH, and 1 µM ArbB2. Assays were incubated at RT for 30 min and completed in triplicate. Reactions were quenched with two volumes of 75% acetonitrile with 0.1% formic acid and centrifuged at maximum speed (21,000 × *g*) for 10 min. Supernatants were collected and subjected to UPLC-HRMS analysis.

Reducing Agents

To determine the optimal in vitro reducing agent for ArbB2 activity, we tested ascorbic acid, cysteine, glutathione, β-mercaptoethanol, 1,4-dithiothreitol, tris(2-carboxyethyl)phosphine hydrochloride, and sodium dithionite. Reactions were performed in triplicate using 50 mM of citrate-phosphate buffer (pH 6.0), 150 µM ArbA2-FLWGY, 1 mM $CuSO_4$, 500 µM reducing agent, and 1 µM ArbB2. Assays were conducted at a 25 µL scale and incubated at RT for 45 min. Reactions were quenched with two volumes of 75% acetonitrile with 0.1% formic acid, centrifuged at maximum speed (21,000 × *g*) for 10 min, and then supernatants were collected and subjected to UPLC-HRMS analysis. Note that stock solutions of reducing agents were freshly prepared to minimize air oxidation.

General Optimized Assay

After determining the optimal in vitro pH, temperature, and reducing agent, a standardized assay condition was used for all studied BpCs and substrates. Reactions (25 µL) were completed in triplicate using 50 mM citrate-phosphate buffer (pH 8.0), 150 µM precursor peptide, 1 mM $CuSO_4$, 500 µM GSH, and 1 µM purified BpC. Assays were incubated at RT for 60 min and quenched with two volumes of 75% acetonitrile with 0.1% formic acid, centrifuged at 21,000 × *g* for 10 min, and then supernatants were collected and subjected to UPLC-HRMS analysis.

Total Turnover

To determine the total turnover (TTO) number of ArbB2, 1 µM ArbB2 was incubated with 300 µM ArbA2-FLWGY, 1 mM $CuSO_4$, and 500 µM GSH in 50 mM citrate-phosphate buffer pH 8.0 at RT for 18 h. The assay was quenched in 50% acetonitrile with 0.1% formic acid and subjected to UPLC-MS analysis. The assays were run in triplicate and a TTO number of 175.06 ± 3.78.

ArbB2 kinetics

Kinetics assays were performed with 50 mM citrate-phosphate pH 6.0, 1 mM CuSO4, 500 µM GSH, 100 nM enzyme, 500 nM, 1, 5, 15, 20, 25, 30, 50, 75, 100, 150, 200, and 250 µM ArbA2-FLWGY incubated at RT and quenched at 2, 4, and 6 minutes with 50% acetonitrile with 0.1% formic acid. These reactions were run in triplicate and analyzed using UPLC-HRMS.

ArbA2 multicore assay

The $ArbA2_{23\text{-}150}$ multicore precursor peptide was reacted with purified ArbB2 in a concentration of 10 µM of substrate and 10 µM of enzyme, 1 mM of $CuSO_4$, 5 µM of GSH, in 50 mM of citrate-phosphate buffer pH 8.0 at room temperature. Every 10 minutes of reaction a 25 µL aliquot was collected and quenched with 2 mM of the copper chelator EDTA (ethylenediaminetetraacetic acid tetrasodium salt hydrate). More GSH was added to the reaction in a final concentration of 5 µM until 50 minutes of total reaction time. The assays were carried out in triplicate. All samples were digested with 6 units of Trypsin-ultra™, Mass Spectrometry Grade (New England Biolabs) for 3 hours at 37 ºC, quenched within 50% acetonitrile with 0.1% formic acid, centrifuged at 21,000 × *g* for 10 min, and then supernatants were collected and subjected to UPLC-MS analysis.

UHPLC-HRMS Method

General UHPLC-HRMS analysis was performed on an Ultimate 3000 UPLC system (Thermo Scientific) coupled to an LTQ Orbitrap Elite (Thermo Scientific) mass spectrometer in positive mode. Compound separation was carried out using the chromatography method as following: solvent A, water with 0.1% formic acid, and solvent B, acetonitrile with 0.1% formic acid. The method was as follows: 15% B for 1 min (directed to waste), 15-30% B gradient for 11 min, 30-100% B for 2 min, and 100-15% B gradient for 1 min. The columns used for chromatographic separation were a Kinetex® 1.7 µM C18 100 Å, 50 x 2.1 mm, ACQUITY UPLC® BEH C18 1.7 µm 2.1 x 50 mm, and bioZen™ 1.7 µm Peptide XB-C18 2.1 x 50 mm column. For MS2 fragmentation, the hundred most abundant ions were selected to generate the b and y ions for fragmentation tables, using a mass tolerance of 20 ppm, mass precision of four decimals, and enabling curve smoothing (Boxcar, 7 points).

Ionization Efficiency

Data processing was performed by utilizing the EIC of the most abundant isotopes of the product and substrate. Their respective peak areas were used to determine the relative amounts of substrate and product in any given assay. For this to be accurate, there should be no change in ionization efficiency. To evaluate this, we compared ionization efficiencies of equimolar amounts of ArbB2-FLWGY and cyclized ArbB2-FLWGY (**Figure S47**). No statistically significant difference was observed.

Exact Masses of Peptides

The following exact masses were used for generation of EICs.

Peptide	Expected m/z Substrate	Expected m/z Product
ArbA2$_{54-77}$	888.1040	887.4321
ArbA2$_{56-77}$	811.7231	811.0512
ArbA2$_{58-77}$ (ArbA2-FLWGY)	744.6981	744.0262
ArbA2-FLWGY-NH2$_{58-77}$	744.3701	743.6982
ArbA2-FVWGY-NH2$_{58-77}$	739.6982	739.0263
ArbA2-FIWGY-NH2$_{58-77}$	744.3701	743.6982
ArbA2$_{59-77}$	725.6909	725.0190
ArbA2$_{60-77}$	687.6766	687.0047
ArbA2$_{61-77}$	653.9941	653.3222
ArbA2$_{62-77}$	616.2994	615.6275
ArbA2$_{64-79}$	611.6154	610.9435
ArbA2$_{65-77}$	728.3488	727.6769
ArbA2$_{58-79}$	821.0547	820.3828
ArbA2$_{58-81}$	887.7479	887.0760
ArbA2-FLWGY F(-7)A	1078.5278	1077.5200
ArbA2-FLWGY S(-1)A	1108.5460	1107.5380
ArbA2-FLWGY Q(+1)A	1087.0265	1086.0190
ArbA2-FLWGY P(-3)L	1124.5592	1123.5510
ArbA2-FLILY$_{58-77}$	1108.0772	1107.0690
ArbA2-VLILY$_{58-77}$	1084.0772	1083.0690
ArbA2-LLLY$_{58-77}$	1034.5430	1033.5350
ArbA6-FLILY$_{168-187}$	1175.5969	1174.5891
ArbA6-VLILY$_{112-131}$	801.0936	800.4217
ArbA7-LLLY$_{87-106}$	1206.6391	1205.6310

CamA1-ILLY$_{113-132}$	731.0296	730.3577
CamA2-ILWY$_{56-75}$	729.0457	728.3738
CamA1-FFFY$_{20-40}$	784.0211	783.3492

Peptide Synthesis and Purification

ArbA2-FLWGY-NH2$_{58-77}$ and two mutants (ArbA2-FLIGY-NH2$_{58-77}$, ArbA2-FLVGY-NH2$_{58-77}$) were synthesized by solid-phase peptide synthesis on a Biotage Initiator+ Alstra Automated Microwave Peptide Synthesizer using 4-(2',4'-Dimethoxyphenyl-Fmoc-aminomethyl) phenoxy resin (rink amide resin). The synthesis of each peptide began at the C-terminus and progressed toward the N-terminus. N-α-Fmoc-L-amino acids were purchased from ChemImpex, and coupling agents N, N'-Diisopropylcarbodiimide (DIC) and Oxyma at 0.5 M solution in dimethylformamide (DMF) using microwave irradiation for each amino acid addition. The process began with swelling the N-terminal Gly with DMF at 70°C for 20 minutes, deprotected with 20% (v/v) piperidine in DMF at 25°C for 13 minutes, coupled using DIC/Oxyma at 75 °C for 5 minutes, then deprotected with 20% (v/v) piperidine in DMF at 25°C for 13 minutes. Following this initial setup, each amino acid was sequentially added with the methods for coupling with DIC/Oxyma at 75°C for 5 minutes and deprotection with 20% (v/v) piperidine in DMF at 25 °C for 13 minutes. Asparagine (N) at the N-terminus of the peptide sequence was coupled with DIC/oxyma at 50 °C for 10 minutes and was deprotected with 20% (v/v) piperidine at 25 °C for 13 minutes. Following the synthesis, the resin was dried after being washed with dichloromethane (DCM) three times with ethyl ether. The dried resin with peptide product was cleaved using a trifluoroacetic acid (TFA) cocktail made of 94.5% (v/v) TFA, 2.5% (v/v) H_2O, 2.5% (v/v) triisopropylsilane (TIPS), and 0.5% (v/v) ethanedithiol. The peptide cleavage was carried out at room temperature for three hours while being stirred at 700 RPM in the peptide synthesis reaction vessel. The reacted solution was pushed through the reaction vessel into a clean vial and dried using line air until the final volume reached about 1 mL. Pre-chilled ethyl ether was added to precipitate the peptide, and the sample was centrifuged, the ethyl ether supernatant was decanted, and the peptide was dried using line air. The dried solid was dissolved in 50% (v/v) acetonitrile-basic water, and the mass of the crude peptide was confirmed using UHLC-HRMS. Peptides were purified using HyperSep™ C18 columns using water and acetonitrile. Synthesized peptides are mainly eluted with water, as these are soluble in water, and some are eluted at 0.1-5% (v/v) acetonitrile. Fractions containing peptide were collected in clean, pre-weighed scintillation vials and dried using the SpeedVac (Savant SpeedVac SPD120 Vacuum Concentrator) connected to a vapor trap (Savant RVT5105 Refrigerated Vapor Trap) and vacuum pump (Thermo Scientific VLP120 Vacuum Pump) overnight, weighed, and then stored at -20°C.

DNA Sequences, Protein Sequences, Peptide Sequences, and Accession Numbers

Protein sequences used

>ArbB2 – BURP cyclase protein [Coffea arabica] XP_027066250.1
MTCKGNNTYQLMDSKALASCVLLLHLLIVLGACDIIPKAKYSGTNAIRLHSMDANNPHRNDKTH
HVAHVHEKKSMHDPSLSSSHMMHQIDPRATVFFVLDDLKLGKTLSILFPDGDPSPLSSPYLWPR
EQADAIPFSLAKLPQILQHFSFPQGSRKAQVMEHALRACETKPMKGEPKACATSYESLVDFARK
ILGLNTDIEVLSTHRLTKSNAARLQNYTITEAPERISTLKMVGCHTMPYPYIVFYCHYQQGDNRLY
RTVLSGENGDRVEGLAICHMDTSQWNHDHVSFQVLGIEPGTAPVCHFFPAEDFVLVPSTSSI

>ArbB2$_{75\text{-}320}$ – truncated expressed protein in *E. coli*
MHDPSLSSSHMMHQIDPRATVFFVLDDLKLGKTLSILFPDGDPSPLSSPYLWPREQADAIPFSL
AKLPQILQHFSFPQGSRKAQVMEHALRACETKPMKGEPKACATSYESLVDFARKILGLNTDIEV
LSTHRLTKSNAARLQNYTITEAPERISTLKMVGCHTMPYPYIVFYCHYQQGDNRLYRTVLSGEN
GDRVEGLAICHMDTSQWNHDHVSFQVLGIEPGTAPVCHFFPAEDFVLVPSTSSI

>ArbB3 – BURP cyclase protein [Coffea arabica] XP_027069063.1
MDSKALVSCVLLLHLLIVLGACDIIPKAKDSGTNAIRLQGMDANNPHRNDKTHHVAHVHEKKSM
HDPSLSSSHMMHQMDPRATVFFVLDDLKLGKTLSILFPDGDPSPLSSPYLWPREQADAIPFSLA
KLPQILQHFSFPQGSHQAQVMEHTLRACETKPMKGESRACATSYESLVDFARKILGLNTDIEVL
STHRLAKSNAARLQNYTITEAPKRISTLKMVGCHNMPYPFIVFGCHYQPGDNNLYRTVLSGENG
DRVEATARCHMDTSQWSHDHVSFRVLGIEPGTAPVCHFFPAEDFVLVPSTSSI

>arbB3 – codon optimized sequence for expression in *E. coli*
ATGGACATTATTCCAAAGGCCAAGGACTCTGGAACAAACGCTATCCGGCTGCAGGGTATGG
ATGCCAATAACCCGCATCGAAATGACAAAACGCATCACGTTGCACACGTGCATGAGAAGAA
GTCGATGCACGACCCATCTCTTTCTTCGAGTCACATGATGCATCAGATGGATCCACGCGCCA
CTGTATTCTTTGTACTGGACGATCTCAAGTTGGGGAAAACCCTTTCTATCCTGTTTCCAGAC
GGAGATCCTAGTCCTCTTAGCAGCCCTTACCTTTGGCCGCGAGAGCAAGCGGATGCCATTC
CTTTCTCACTTGCCAAACTGCCACAAATCCTGCAGCACTTTTCTTTCCCGCAAGGCTCACAC
CAAGCACAAGTAATGGAGCACACATTGCGAGCCTGCGAGACGAAGCCGATGAAGGGAGAG
TCGAGAGCCTGTGCTACTAGCTACGAAAGTCTGGTTGACTTCGCTCGTAAGATCTTGGGATT
AAATACGGACATTGAAGTTTTATCAACGCATCGTCTCGCCAAGAGCAACGCGGCGCGCCTT
CAGAATTATACTATTACTGAAGCTCCGAAGCGCATATCTACCCTTAAGATGGTGGGCTGCCAC
AATATGCCCTACCCGTTCATTGTTTTCGGTTGTCACTACCAACCTGGGGACAACAACTTGTA
CCGTACAGTGTTATCTGGAGAAAACGGCGATCGAGTCGAGGCTACCGCGCGATGCCATATG
GACACGTCTCAATGGTCCCACGACCACGTCAGTTTTCGGGTGCTGGGCATCGAACCAGGC
ACGGCGCCAGTTTGTCACTTCTTCCCGGCAGAGGACTTTGTGCTGGTTCCCAGCACAAGTT
CAATCTAG

>ArbB3$_{23\text{-}288}$– truncated expressed protein sequence in *E. coli*
DIIPKAKDSGTNAIRLQGMDANNPHRNDKTHHVAHVHEKKSMHDPSLSSSHMMHQMDPRATV
FFVLDDLKLGKTLSILFPDGDPSPLSSPYLWPREQADAIPFSLAKLPQILQHFSFPQGSHQAQVM
EHTLRACETKPMKGESRACATSYESLVDFARKILGLNTDIEVLSTHRLAKSNAARLQNYTITEAP
KRISTLKMVGCHNMPYPFIVFGCHYQPGDNNLYRTVLSGENGDRVEATARCHMDTSQWSHDH
VSFRVLGIEPGTAPVCHFFPAEDFVLVPSTSSI

>CamB1
MAKGFASCVLILYLFFLMCSSGNGSDHQESANIVENHSHAHNNPMSHNMDYDMYMAPRVGFF
TGDDLHVGKTMTVQFFTKDPSSLPPFLSREEADRIPFSLNEFPHLLKLFSFSQGSHEAKLIERTL
QTCAQKPIVGERKTCATSKESLVEFVSSVLGGRNGVDFRALKSTHLGKPSSTFQNYTFLDVKEV
NSPNMVACHIMDYPYAVFVCHTQTSKVYQILLAGHEDGDIINAVAVCHIDTSHWAPDHISFRFLG
VKPGTVPVCHFFGPHNLIWVQN

>camB1- codon optimized sequence for expression in *E. coli*
ATGGCTAAGGGGTTTGCATCCTGCGTGTTAATACTGTACCTTTTCTTTCTCATGTGCAGTTCG
GGTAACGGTAGCGACCATCAGGAAAGCGCTAACATCGTTGAGAACCACTCTCACGCCCACA
ACAATCCCATGAGTCACAACATGGATTACGATATGTACATGGCGCCTAGAGTAGGCTTCTTCA
CTGGGGACGATCTGCACGTCGGCAAGACTATGACAGTTCAATTCTTCACTAAAGACCCAAG
CTCTCTTCCACCCTTCCTCTCACGTGAAGAGGCCGATCGCATTCCTTTCTCTCTTAATGAGT

TCCCTCACTTGCTTAAGCTTTTCAGTTTCTCACAAGGGTCCCACGAGGCTAAGCTGATCGAA
AGAACCCTGCAAACTTGTGCACAAAAGCCTATAGTTGGCGAGAGAAAGACGTGCGCCACGT
CTAAGGAATCCCTGGTAGAGTTTGTCTCGTCTGTCCTTGGTGGAAGAAATGGGGTAGACTT
CCGGGCGCTCAAAAGCACACACTTGGGTAAACCATCGTCAACATTCCAAAACTATACTTTCT
TAGATGTCAAGGAAGTGAATTCGCCCAATATGGTAGCTTGTCACATTATGGATTATCCATACG
CCGTTTTCGTTTGTCATACACAAACGAGTAAAGTGTATCAGATCCTGTTAGCGGGCCATGAA
GACGGCGATATCATCAACGCGGTTGCTGTGTGTCACATCGACACGAGTCATTGGGCCCCCG
ACCACATTAGCTTCCGCTTCCTCGGCGTGAAACCGGGGACTGTACCAGTCTGTCATTTCTT
CGGTCCACACAACCTCATTTGGGTCCAAAACTAA

>CamB1$_{23\text{-}278}$ truncated expressed protein sequence in *E. coli*
NGSDHQESANIVENHSHAHNNPMSHNMDYDMYMAPRVGFFTGDDLHVGKTMTVQFFTKDPS
SLPPFLSREEADRIPFSLNEFPHLLKLFSFSQGSHEAKLIERTLQTCAQKPIVGERKTCATSKESL
VEFVSSVLGGRNGVDFRALKSTHLGKPSSTFQNYTFLDVKEVNSPNMVACHIMDYPYAVFVCH
TQTSKVYQILLAGHEDGDIINAVAVCHIDTSHWAPDHISFRFLGVKPGTVPVCHFFGPHNLIWVQ
N

>ArbA2 – precursor peptide [Coffea arabica] XP_027066141.1
MASSITLIAVFSIALFACITEARKNPTDFLQSAVINEHTEDNHHAESSLSNQKKTSNGNTLKDFES
KPGSFLWGYQGNDAESKSKEEKPLMKGFESKPGSFLWGYQGNDAESKSKEEKPLMKGFESK
PGSFLWGYQGNDVESKSKEEKPLMKDFESKPGSFL

arbA2 – codon optimized sequence for expression in *E.coli*
ATGCGTAAGAATCCTACGGACTTTCTGCAATCGGCCGTGATAAACGAGCACACCGAGGATAA
CCACCATGCAGAGAGTTCCCTGTCAAACCAGAAGAAGACGAGCAATGGAAATACCCTCAAG
GATTTCGAATCTAAGCCGGGATCTTTCTTGTGGGGATATCAAGGTAATGATGCCGAGTCCAA
GTCTAAGGAAGAGAAGCCCCTGATGAAGGGTTTCGAGTCCAAGCCGGGTTCCTTCTTATGG
GGTTACCAAGGGAATGATGCCGAGAGTAAATCGAAAGAGGAGAAGCCGCTGATGAAAGGAT
TCGAGTCAAAGCCTGGCTCTTTCTTGTGGGGTTATCAAGGTAACGACGTAGAAAGTAAATCG
AAAGAGGAAAAGCCTCTTATGAAGGATTTCGAATCGAAGCCGGGTTCGTTTCTGTAG

>ArbA2$_{23\text{-}150}$ – truncated expressed multicore precursor peptide sequence in *E. coli*
RKNPTDFLQSAVINEHTEDNHHAESSLSNQKKTSNGNTLKDFESKPGSFLWGYQGNDAESKSK
EEKPLMKGFESKPGSFLWGYQGNDAESKSKEEKPLMKGFESKPGSFLWGYQGNDVESKSKE
EKPL

>ArbA2$_{23\text{-}150}$ – truncated multicore precursor peptide purified sequence
SGSMRKNPTDFLQSAVINEHTEDNHHAESSLSNQKKTSNGNTLKDFESKPGSFLWGYQGNDA
ESKSKEEKPLMKGFESKPGSFLWGYQGNDAESKSKEEKPLMKGFESKPGSFLWGYQGNDVE
SKSKEEKPL

>ArbA1 – precursor peptide [Coffea arabica] XP_027065604.1
MASSITLIAVFSIALFACITEARKNPTDFLQSAVINEHTEDNHHAESSLSNQKKTSNGNTLKDFES
KPGSFLWGYQGNDAESKSKEEKPLMKGFESKPGSFLWGYQGNDAESKSKEEKPLMKGFESK
PGSFLWGYQGNDVESKSKEEKPLTKDFESKPGSFLWGYQGNHAESKSKKEKPLMKDFESKPG
SFLWGYQGNHAEYKEKKPLVKDN

>ArbA3 – precursor peptide [Coffea arabica] XP_027066439.1
MASSITLIAVFSIALFACITEARKNPTDFLQSAVINEHTEDNHHAESSLSNQKKTSNGNTLKDFES
KPGSFLWGYQGNEAESKSKEEKPLMKGFESKPGSFLWGYQGNDVESKSKEEKPFMKGFESK

PGSFLWGYQGNNAESKSKEENPLMKDFESKPGSFLWGYQGNDAESKSEEEKPLMKDFELKP
GSFLWGYQGNHAEYKEEKPLVKDN

>ArbA4 – precursor peptide [Coffea arabica] XP_027065408.1
MASSITLIAVFSIALFGCITEARKNPTDFLQSAVINEHTEDNHHAEPSPSNQKKTTNGNTLKDFES
KRGSHLFYHGDDASPLVKDFESKLGSLLLYHYGAEEKPLRKDSLFGAPLRKDFESKLGSLLLYH
YGAEEKPLRKDFESKLGSLLLYHYGAEEKTLMKDFESKRGSHLFYHGDDAKPMKDN

>ArbA5 – precursor peptide [Coffea arabica] XP_027065343.1
MASSITLIAVFSIALFGCITEARKNPTDFLQSAVINEHTEDNHHAEPSPSNQKKTTNGNTLKDFES
KRGSHLFYHGDDASPLVKDFESKLGSLLLYHYGAEEKPLRKDSLFGAPLRKDFESKLGSLLLYH
YGAEEKPLRKDFESKLGSLLLYHYGAEEKTLMKDFESKRGSHLFYHGDDAKPMKDN

>ArbA6 – precursor peptide [Coffea arabica] XP_027086167.1
MRSSVALVAFFSIALLACFTEARKDPRGILRPAASPGAFTEQNEHLGSNTLNEFESKPGSILHAD
EPRSILPYHGRDANSKEEKPQMKDFESKSESVLLFYGGDKANLQEAKPYMKDFESKPESVLILY
RGDKANLQEAKPHMKDFESKPESVLLFYGGDKANLQEAKPYMKDFESKPESFLILYGGDKANL
QEAKPHMKDFESKPESVLPFYGGDKANLQEAKPYMKDFESKPESFLILYGGDKANLQEGKLHI

>ArbA7 – precursor peptide [Coffea arabica] XP_027086167.1
MASSITLIAVFSIALFACITEARKNPTDSLQSAVINEHTEDNHHAELSLSNQKKTSDGNTLKDFES
KPGSLLWNYQGNHAESKSKEEKPLMKDFESKLGSLLLYHYQGNHAESKSKEEKPLMKDFESKL
G

>MBP-Tag sequence fused to N-term
MGSSHHHHHHSSGLVPRGSHMKIEEGKLVIWINGDKGYNGLAEVGKKFEKDTGIKVTVEHPDK
LEEKFPQVAATGDGPDIIFWAHDRFGGYAQSGLLAEITPDKAFQDKLYPFTWDAVRYNGKLIAYP
IAVEALSLIYNKDLLPNPPKTWEEIPALDKELKAKGKSALMFNLQEPYFTWPLIAADGGYAFKYEN
GKYDIKDVGVDNAGAKAGLTFLVDLIKNKHMNADTDYSIAEAAFNKGETAMTINGPWAWSNIDT
SKVNYGVTVLPTFKGQPSKPFVGVLSAGINAASPNKELAKEFLENYLLTDEGLEAVNKDKPLGA
VALKSYEEELAKDPRIAATMENAQKGEIMPNIPQMSAFWYAVRTAVINAASGRQTVDEALKDAQ
TNSSSHHHHHANSVPLVPRGSENLYFQSGS

Precursor peptides used in this study

<u>ArbA2 Lengths:</u>

ArbA2$_{54\text{-}77}$: KTSNGNTLKDFESKPGSFLWGYQG
ArbA2$_{56\text{-}77}$: SNGNTLKDFESKPGSFLWGYQG
ArbA2$_{58\text{-}77}$ (ArbA2-FLWGY): GNTLKDFESKPGSFLWGYQG
ArbA2$_{59\text{-}77}$: NTLKDFESKPGSFLWGYQG
ArbA2$_{60\text{-}77}$: TLKDFESKPGSFLWGYQG
ArbA2$_{61\text{-}77}$: LKDFESKPGSFLWGYQG
ArbA2$_{62\text{-}77}$: KDFESKPGSFLWGYQG
ArbA2$_{64\text{-}79}$: FESKPGSFLWGYQGND
ArbA2$_{65\text{-}77}$: ESKPGSFLWGYQG
ArbA2$_{58\text{-}79}$: GNTLKDFESKPGSFLWGYQGND
ArbA2$_{58\text{-}81}$: GNTLKDFESKPGSFLWGYQGNDAE
ArbA2-FLWGY-NH2$_{58\text{-}77}$: GNTLKDFESKPGSFLWGYQG-NH_2

<u>ArbA2 Mutations:</u>

ArbA2-FLWGY F(-7)A: GNTLKDAESKPGSFLWGYQG
ArbA2-FLWGY S(-1)A: GNTLKDFESKPGAFLWGYQG
ArbA2-FLWGY Q(+1)A: GNTLKDFESKPGSFLWGYAG
ArbA2-FLWGY P(-3)L: GNTLKDFESKLGSFLWGYQG
ArbA2-FIWGY-NH2$_{58-77}$: GNTLKDFESKPGSFIWGYQG-NH_2
ArbA2-FVWGY-NH2$_{58-77}$: GNTLKDFESKPGSFVWGYQG-NH_2

C. arabica Cores:
ArbA2-FLILY$_{58-77}$: GNTLKDFESKPGSFLILYQG
ArbA2-VLILY$_{58-77}$: GNTLKDFESKPGSVLILYQG
ArbA2-LLLY$_{58-77}$: GNTLKDFESKPGSLLLYQG
ArbA6-FLILY$_{168-187}$: KPYMKDFESKPESFLILYGG
ArbA6-VLILY$_{112-131}$: KPYMKDFESKPESVLILYRG
ArbA7-LLLY$_{87-106}$: EKPLMKDFESKLGSLLLYHY

C. americanus Peptides
CamA1-ILLY$_{113-132}$: EGELKDFSVDPSGNILLYHG
CamA2-ILWY$_{56-75}$: GRVAKDISVDPSGNILWYHG
CamA1-FFFY$_{20-40}$: EGELKDFSVDPSGNFFFYHN

Primers peptides used in this study
pET28-MBP-up: GGATCCGGATTGGAAGTACAGGTTCTCAGATCC
pET28-MBP-down: CTCGAGCACCACCACCACCACCACTGAG
arbB3_F: CTGTACTTCCAATCCGGATCCATGGACATTATTCCAAAGGCCAAGG
arbB3_R: GGTGGTGGTGGTGCTCGAGCTAGATTGAACTTGTGCTGGGAACC
arbB2-H163A_F: CAAGATGGTGGGTTGTGCCACCATGCCCTACCCC
arbB2-H163A_R: GGGGTAGGGCATGGTGGCACAACCCACCATCTTG
arbB2-H175A_F: CTATATCGTGTTCTATTGCGCCTATCAGCAGGGCGACAAT
arbB2-H175A_R: ATTGTCGCCCTGCTGATAGGCGCAATAGAACACGATATAG
arbB2-H203A_F: GGAAGGTTTGGCAATTTGCGCTATGGACACAAGTCAATGG
arbB2-H203A_R: CCATTGACTTGTGTCCATAGCGCAAATTGCCAAACCTTCC
arbB2-H230A_F: CGCGCCCGTCTGCGCTTTCTTCCCGGCA
arbB2-H230A_R: TGCCGGGAAGAAAGCGCAGACGGGCGCG
arbB2-K82A_F: CCGCAAGGTAGCCGCGCGGCACAGGTTATGGA
arbB2-K82A_R: TCCATAACCTGTGCCGCGCGGCTACCTTGCGG
arbB2-P168A_F: GTCACACCATGCCCTACGCCTATATCGTGTTCTAT
arbB2-P168A_R: ATAGAACACGATATAGGCGTAGGGCATGGTGTGAC
arbB2-M86A_F: GCCGCAAGGCACAGGTTGCGGAACACGCCTTAC
arbB2-M86A_R: GTAAGGCGTGTTCCGCAACCTGTGCCTTGCGGC
arbA2-FLWGY-multicore_F: ATGCGTAAGAATCCTACGGACTTTCTGC
arbA2-FLWGY-multicore_R: AAGAGGCTTTTCCTCTTTCGATTTACTTTCTAC
CamB1_G24_F: CTGTACTTCCAATCCGGATCCATGAACGGTAGCGACCATCAGGAAAGCGC
CamB1_R:
GGTGGTGGTGGTGCTCGAGTTATTAGTTTTGGACCCAAATGAGGTTGTGTGGACCG

Protein Accession Numbers
ArbB1: XP_027067479.1
ArbB2: XP_027066250.1
ArbB3: XP_027069063.1
ArbA1: XP_027065604.1
ArbA2: XP_027066141.1
ArbA3: XP_027066439.1
ArbA4: XP_027065408.1
ArbA5: XP_027065343.1
ArbA6: XP_027086167.1
ArbA7: XP_027072356.1
CamB1: PV340619
CamA1: PV340617
CamA2: PV340616

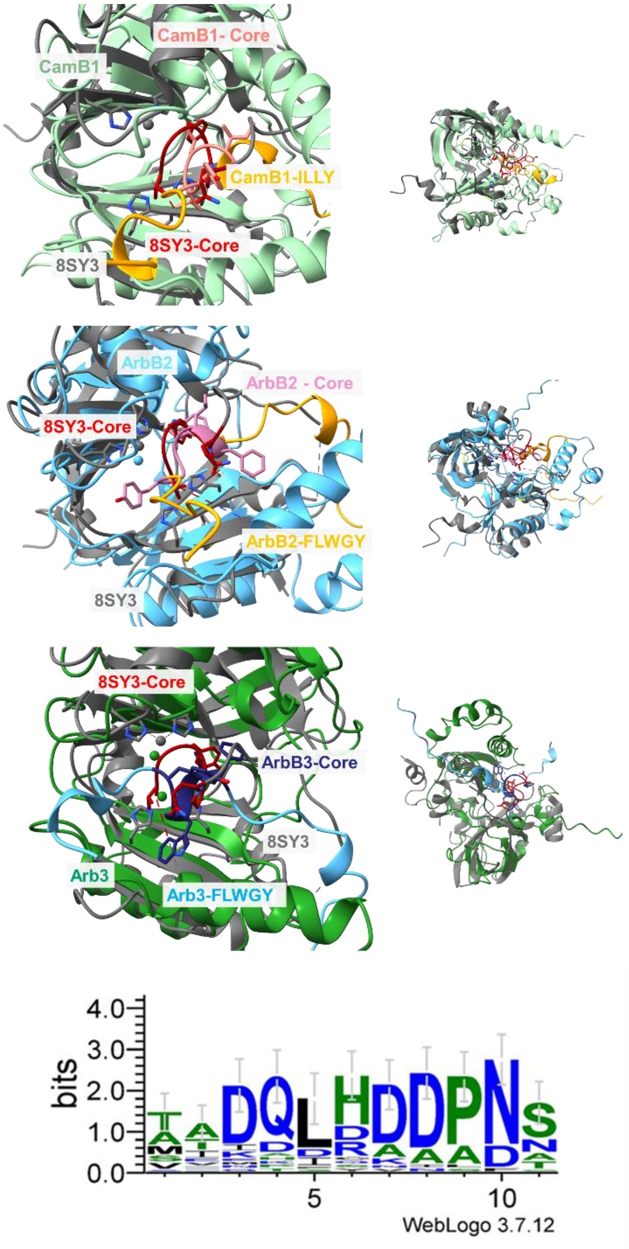

Figure S1. AlphaFold 3 models of ArbB2$_{75\text{-}320}$, CamB1$_{23\text{-}278}$, and ArbB3$_{23\text{-}288}$ aligned to the experimentally determined X-ray crystal structure of AhyBURP (PDB: 8SY2). WebLogo generated with 25 unique recognition sequences found KjaBURP, BtoBURP, CcaBURP1, CcaBURP2, SkrBURP, and AhyBURP.

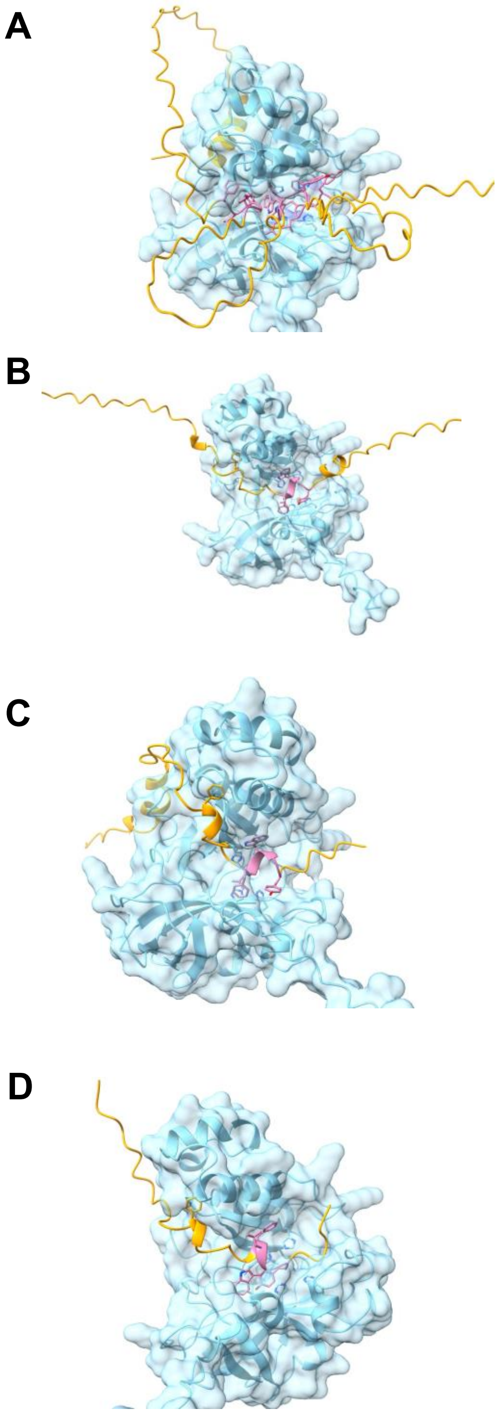

Figure S2. AlphaFold3 models – A) ArbB2 with ArbA2 full length without the signal peptide sequence (141mer); B) ArbB2 with truncated ArbA2 (79mer); C) ArbB2 BpC with truncated ArbA2 (45mer); D) ArbB2 with truncated ArbA2 (30mer).

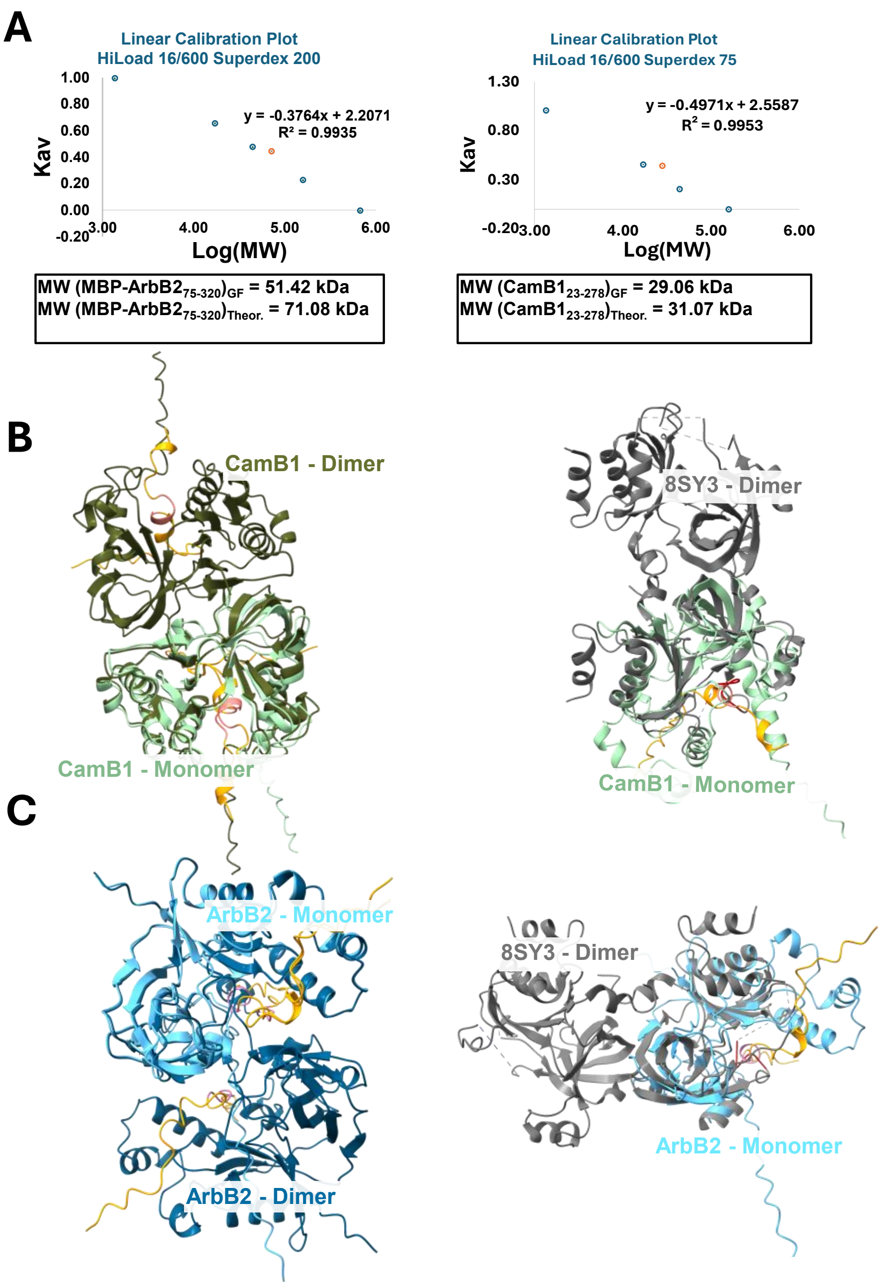

Figure S3. A) Left- Linear calibration plot for HiLoad 16/600 Superdex 200 size exclusion chromatography column. Orange point indicates the data for MBP-ArbB2$_{75\text{-}320}$. Because MBP-ArbB2$_{75\text{-}320}$ is a fusion protein with a flexible linker, it may not behave like a globular protein. However, the observed predicted molecular weight is much more consistent with a monomer than a dimer. Right- Linear calibration plot for HiLoad 16/600 Superdex 75 size exclusion chromatography column. Orange point indicates the data for CamB1$_{23\text{-}278}$. The observed mass is more consistent with a monomer than a dimer. B) Left-: CamB1-monomer alignment with CamB1-dimer. Right: CamB1-monomer alignment with AhyBURP Crystal Structure. C) Left- ArbB2-monomer alignment with ArbB2-dimer. Right: ArbB2-monomer alignment with AhyBURP Crystal structure. The lack of changes in the AlphaFold3 models between the monomeric and dimeric state further support the observed monomer by gel filtration analysis

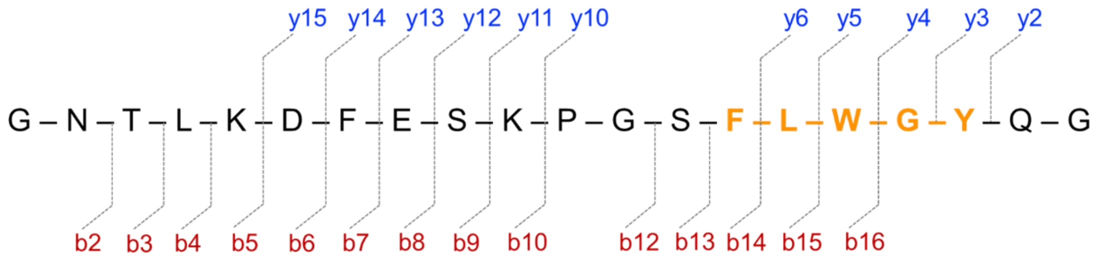

Fragment (z)	m (obs)	m (cal)	Δm (ppm)
b2(+1)	172.0715	172.0717	0.99
b3(+1)	273.1192	273.1193	0.55
b4(+1)	386.2042	386.2034	2.05
b5(+1)	514.2996	514.2984	2.39
b6(+1)	629.3263	629.3253	1.57
b7(+1)	776.3951	776.3937	1.78
b8(+1)	905.4382	905.4363	2.09
b9(+1)	992.4716	992.4683	3.28
b10(+1)	1120.5633	1120.5633	0
b11(+1)	-	1217.6161	-
b12(+1)	1274.6249	1274.6375	9.88
b13(+1)	1361.6642	1361.6696	3.96
b14(+1)	1508.7320	1508.7380	3.95
b15(+1)	1621.8141	1621.8220	4.88
b16(+1)	1807.8928	1807.9013	4.72
b17(+1)	-	1864.9228	-
b17(+2)	932.9643	932.9651	0.86
b18(+1)	-	2027.9861	-
b19(+1)	-	2213.0662	-

Fragment (z)	m (obs)	m (cal)	Δm (ppm)
y2(+1)	204.0977	204.0979	1.08
y3(+1)	367.1626	367.1612	3.68
y4(+1)	424.1865	424.1827	8.93
y5(+1)	610.2627	610.2620	1.11
y6(+1)	723.3455	723.3461	0.80
y7(+1)	-	870.4145	-
y8(+1)	-	957.4465	-
y9(+1)	-	1014.4680	-
y10(+1)	1111.5185	1111.5207	2.02
y11(+1)	1239.6125	1239.6157	2.58
y12(+1)	1326.6470	1326.6477	0.55
y13(+1)	1455.6899	1455.6903	0.29
y14(+1)	1602.7535	1602.7587	3.26
y15(+1)	1717.7831	1717.7857	1.50
y16(+1)	-	1845.8806	-
y17(+1)	-	1958.9647	-
y18(+1)	-	2060.0124	-
y19(+1)	-	2174.0553	-

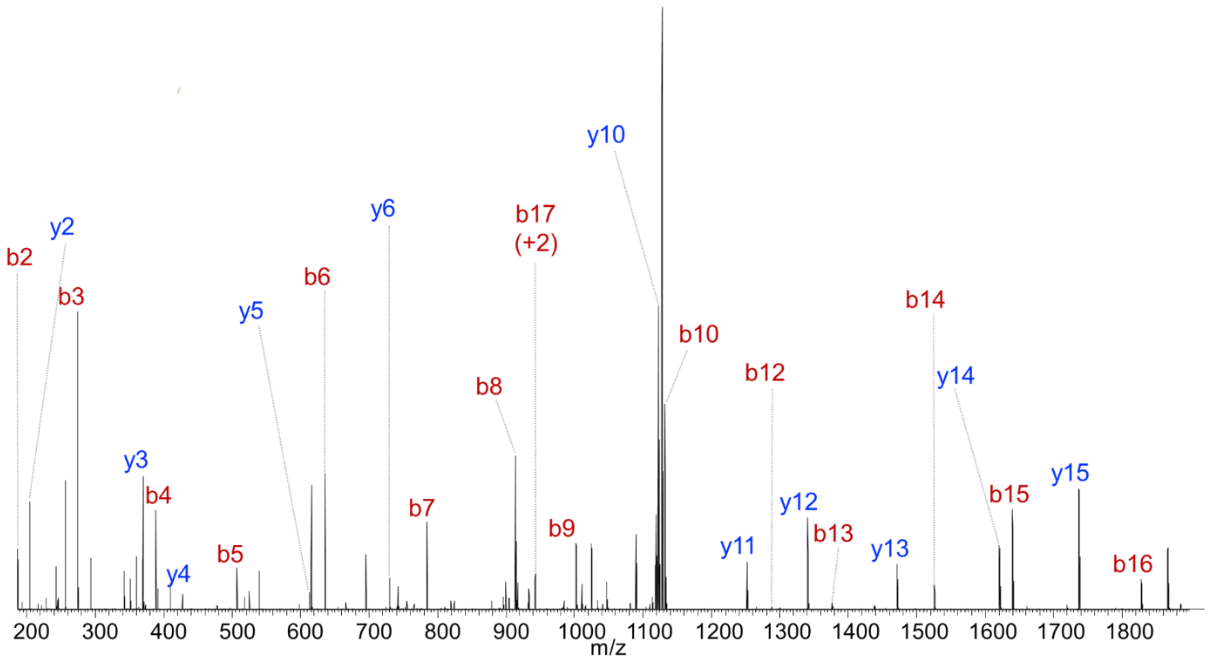

Figure S4. MS2 fragmentation of linear ArbA2-FLWGY substrate. Core peptide is highlighted in orange.

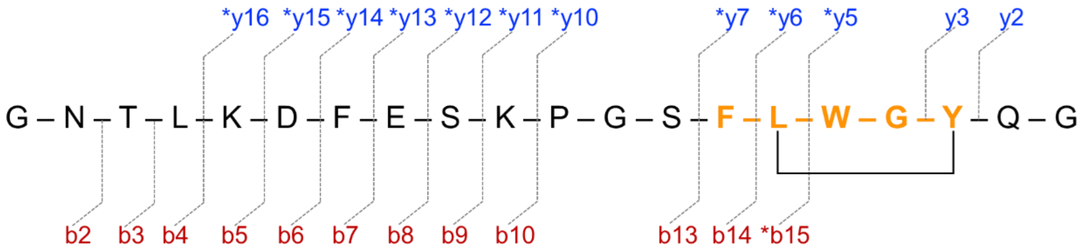

Fragment (z)	m (obs)	m (cal)	Δm (ppm)
b2(+1)	172.0717	172.0715	0.99
b3(+1)	273.1192	273.1193	0.55
b4(+1)	386.2033	386.2034	0.28
b5(+1)	514.2989	514.2984	1.03
b6(+1)	629.3262	629.3253	1.41
b7(+1)	776.3931	776.3937	0.80
b8(+1)	905.4344	905.4363	2.11
b9(+1)	992.4699	992.4683	1.57
b10(+1)	1120.5611	1120.5633	1.96
b11(+1)	-	1217.6161	-
b12(+1)	-	1274.6375	-
b13(+1)	1361.6626	1361.6696	5.10
b14(+1)	1508.7342	1508.7380	2.49
b15(+1)*	1619.8011	1619.8064	3.25
b16(+1)*	-	1805.8857	-
b17(+1)*	-	1862.9071	-
b18(+1)*	-	2025.9705	-
b19(+1)*	-	2154.0290	-

Fragment (z)	m (obs)	m (cal)	Δm (ppm)
y2(+1)	204.0979	204.0979	0.10
y3(+1)	367.1611	367.1612	0.27
y4(+1)	-	424.1827	-
y5(+1)	610.2626	610.2620	0.95
*y6(+1)	-	721.3304	-
*y7(+1)	-	868.3988	-
*y8(+1)	-	955.4309	-
*y9(+1)	-	1012.4523	-
*y10(+1)	1109.5023	1109.5051	2.51
*y11(+1)	1237.5985	1237.6000	1.24
*y12(+1)	1324.6306	1324.6321	1.11
*y13(+1)	1453.6731	1453.6747	1.07
*y14(+1)	1600.7432	1600.7431	0.08
*y15(+1)	1715.7674	1715.7700	1.52
*y16(+1)	1843.8679	1843.8650	1.59
*y17(+1)	-	1956.9490	-
*y18(+1)	-	2057.9967	-
*y19(+1)	-	2172.0396	-

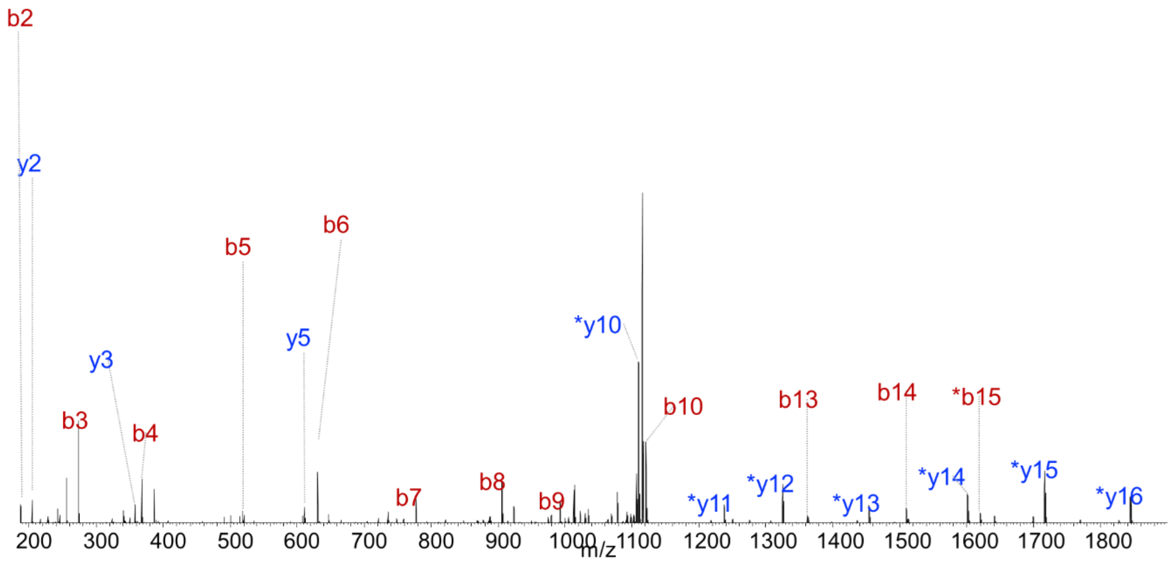

Figure S5. MS2 fragmentation of cyclic ArbA2-FLWGY product. Stars (*) indicate the loss of two hydrogens in the MS2 fragment ions. Core peptide is highlighted in orange.

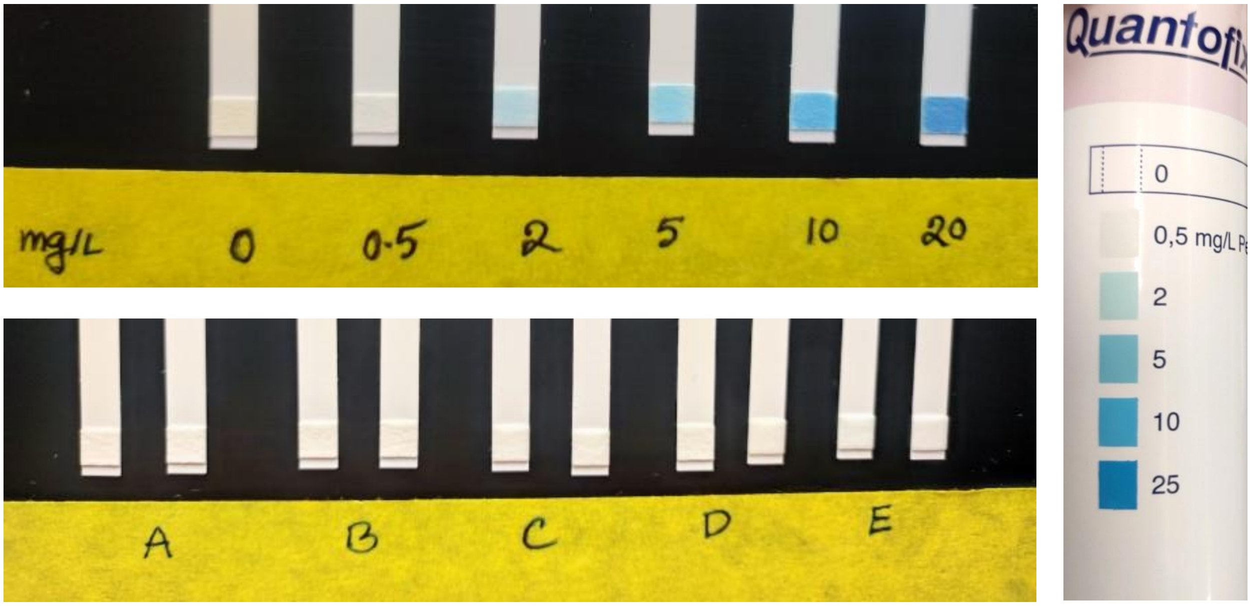

Figure S6. H_2O_2 detection experiment. The experiments were conducted using the same reaction compositions, 50 mM citrate-phosphate buffer (pH 8.0) with 1 mM $CuSO_4$, 500 μM glutathione, and 150 μM ArbA2-FLWGY substrate, but with varying concentrations of H_2O_2 (top) in place of enzyme (bottom). The presence of H_2O_2 was monitored using semiquantitative QUANTOFIX peroxide test strips at different time intervals (A = 5 min, B = 15 min, C = 30 min, D = 60 min, E = 120 min). Two different enzyme to substrate concentrations were tested (1:150 on the left, 2:300 on the right, respectively) to ensure detection of even trace amounts of hydrogen peroxide.

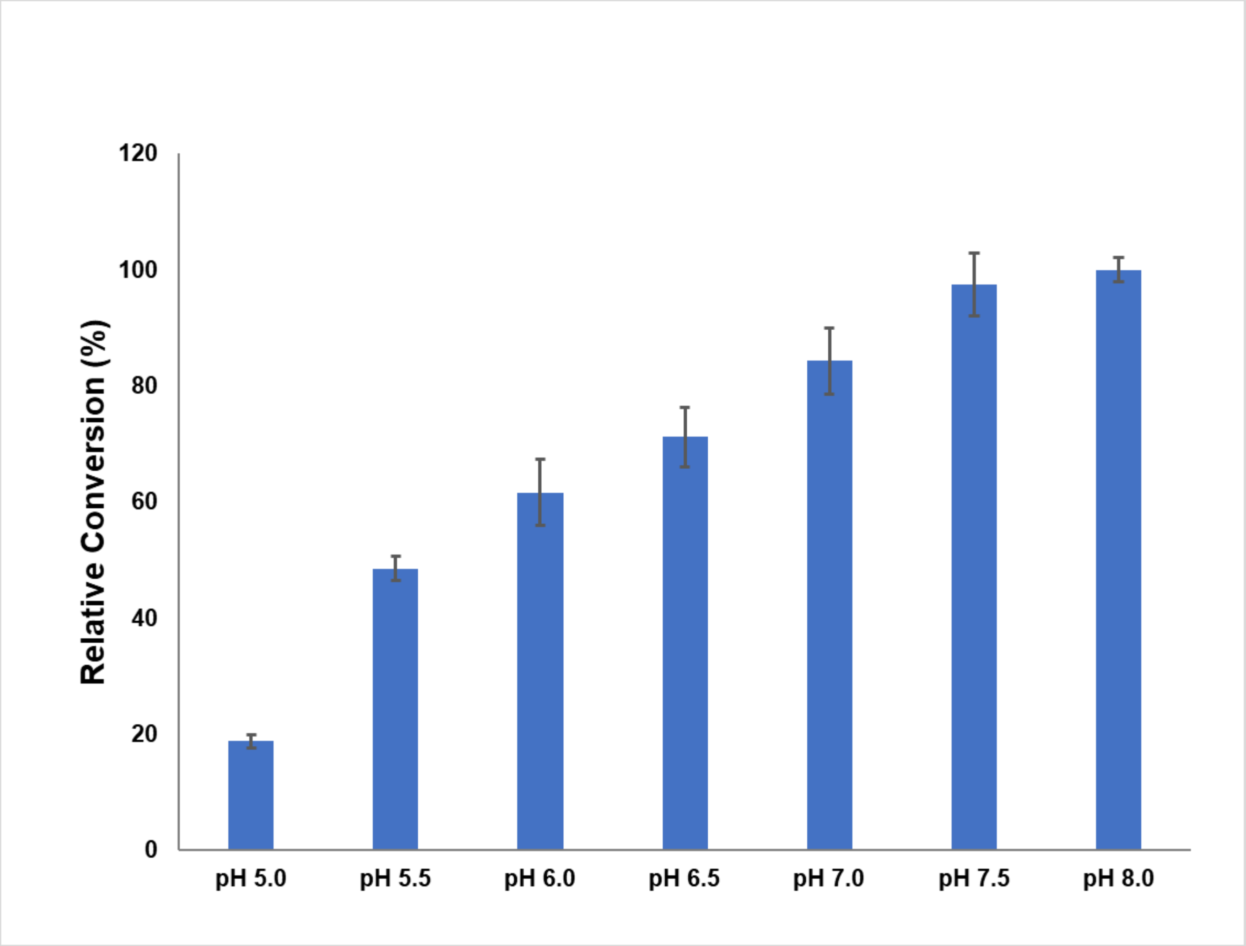

Figure S7. ArbB2 optimal pH. Assays were performed in citrate-phosphate buffer varying the pH (5.0, 5.5, 6.0, 6.5, 7.0, 7.5, and 8.0) in a stoichiometric ratio of 1:150 enzyme/substrate and quenched after 1 hour. Error bars indicated the standard deviation of three trials.

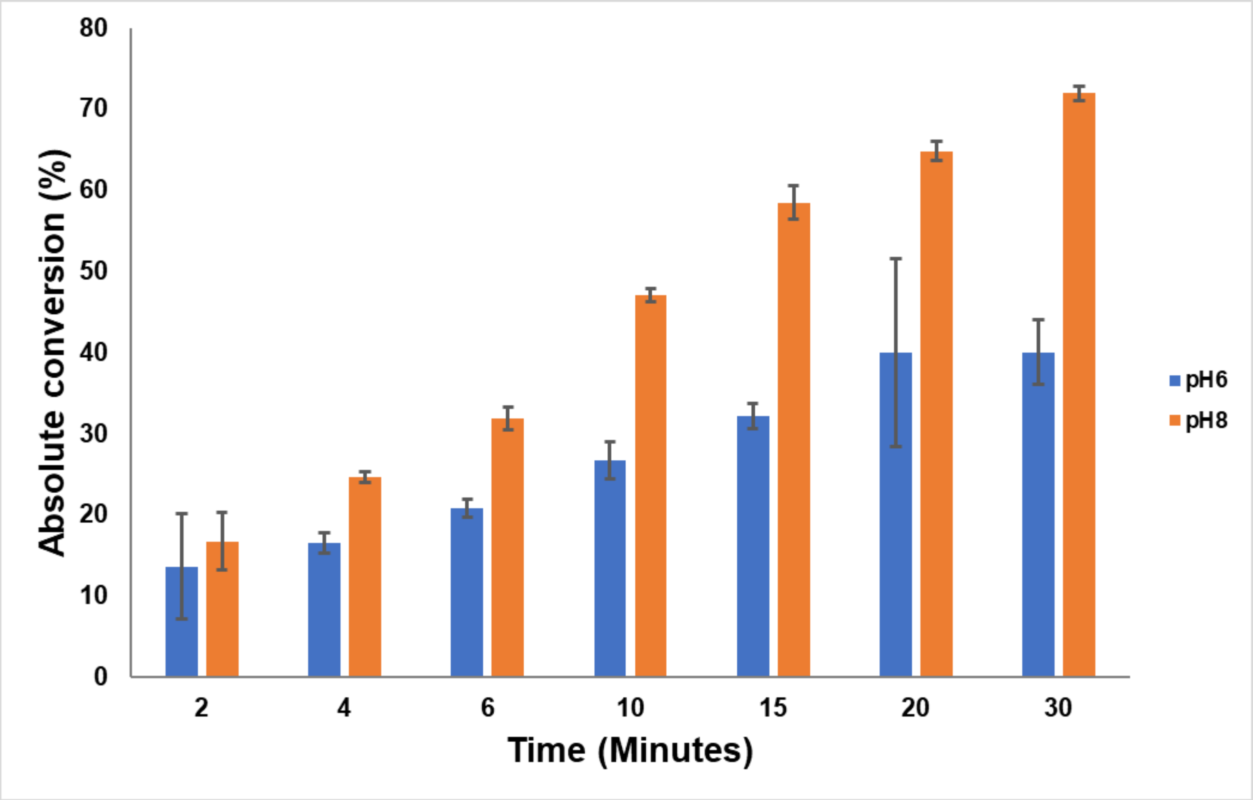

Figure S8. ArbB2 time course assay was carried out as described before. Assays were performed in citrate-phosphate buffer (pH 6.0 and pH 8.0) in a stoichiometric ratio of 1:150 enzyme/substrate and quenched after 2, 4, 6, 10, 15, 20, and 30 minutes. Error bars indicated the standard deviation of three trials.

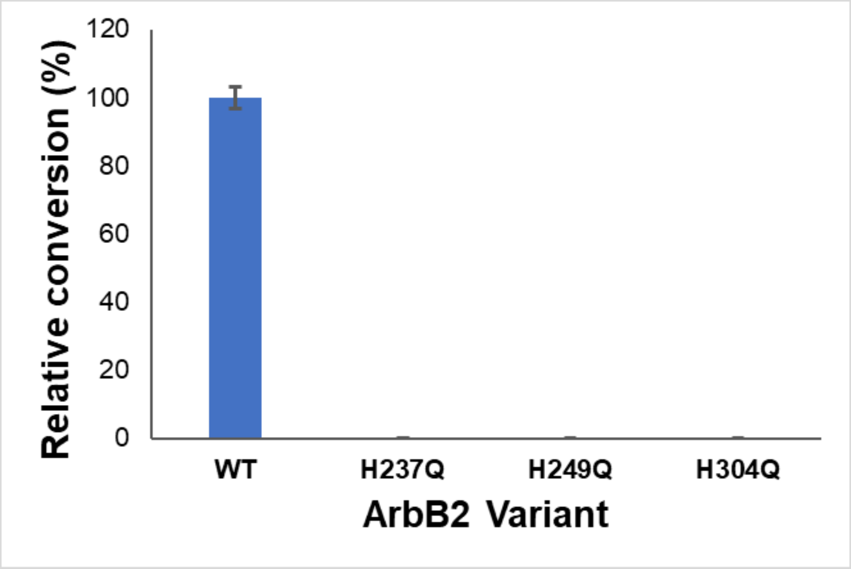

Figure S9. Impact of amino acid substitution on ArbB2 activity. Assays were performed in citrate-phosphate buffer (pH 8.0), in a ratio of 1:150 enzyme/substrate, at room temperature, and quenched after 60 minutes. Error bars indicated the standard deviation of three trials.

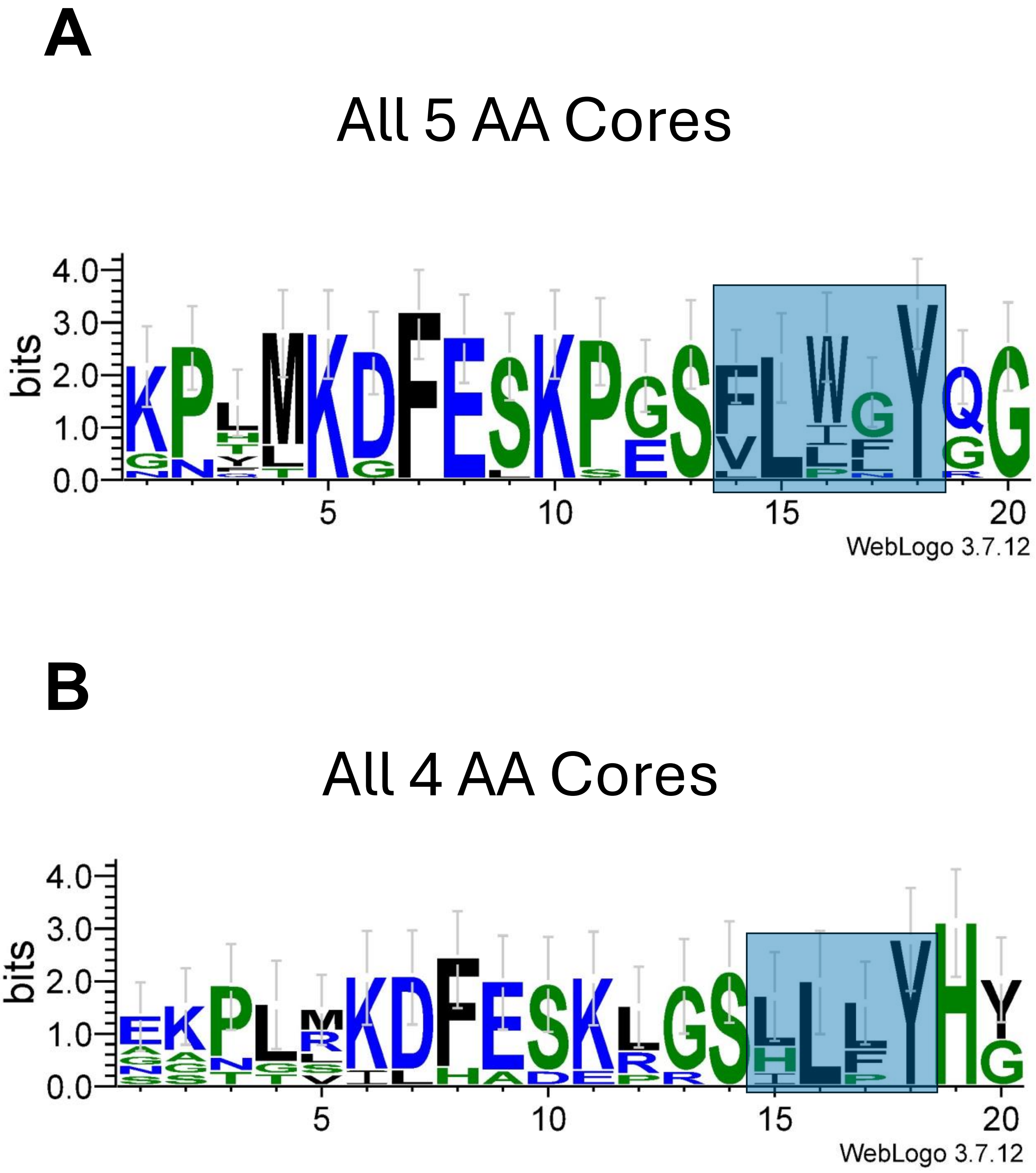

Figure S10. *C. arabica* Weblogos generated with: A) 13 aligned unique precursor peptides containing five residues core and B) seven aligned unique precursor peptides containing four residues core. The highlighted sequence is the predicted core motif, and all precursor peptides were 20mer in length.

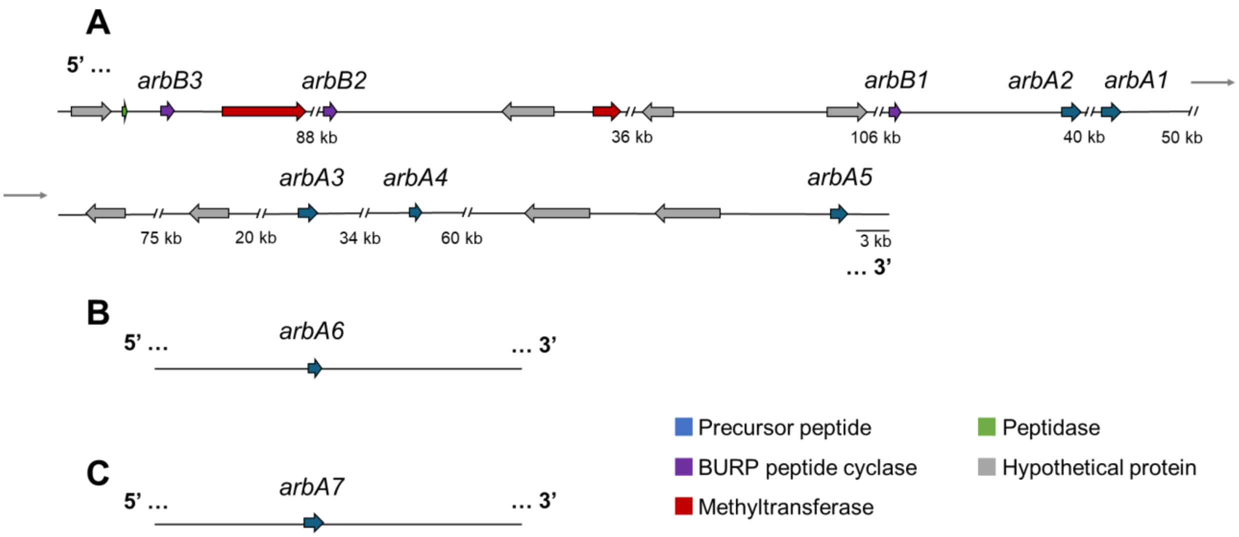

Figure S11. *C. arabica* biosynthetic gene cluster (*arb*). A) genes located at chromosome 6c; B) gene *arbA6* located at chromosome 9c; C) gene *arbA7* located at chromosome 6e. Protein accession numbers are indicated in the experimental section.

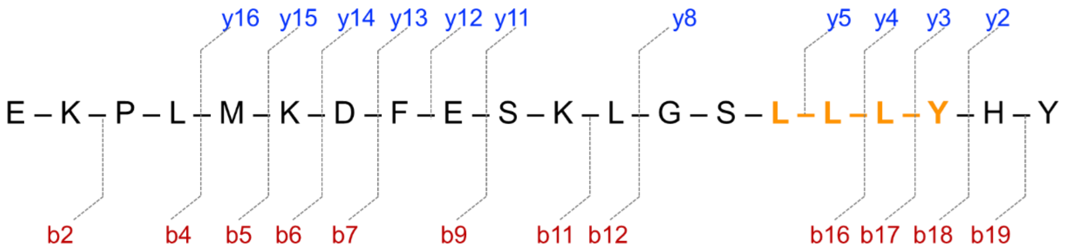

Fragment (z)	m (obs)	m (cal)	Δm (ppm)
b2(+1)	258.1446	258.1448	0.77
b3(+1)	-	355.1976	-
b4(+1)	468.2817	468.2816	0.13
b5(+1)	599.3217	599.3221	0.75
b6(+1)	727.4180	727.4171	1.21
b7(+1)	842.4440	842.4440	0.02
b8(+1)	-	989.5124	-
b9(+1)	1118.5576	1118.5550	2.26
b10(+1)	-	1205.5871	-
b11(+1)	1333.6842	1333.6820	1.64
b12(+1)	1446.7674	1446.7661	0.91
b13(+1)	-	1503.7876	-
b14(+1)	-	1590.8196	-
b15(+1)	-	1703.9037	-
b16(+1)	1816.9849	1816.9877	1.54
b16(+2)	908.9961	908.9975	1.54
b17(+1)	1930.0840	1930.0718	6.35
b17(+2)	965.5457	965.5395	6.35
b18(+2)	1047.0790	1047.0712	7.49

Fragment (z)	m (obs)	m (cal)	Δm (ppm)
y2(+1)	319.1396	319.1401	1.54
y3(+1)	482.2029	482.2035	1.16
y4(+1)	595.2880	595.2875	0.81
y5(+1)	708.3717	708.3716	0.17
y6(+1)	-	821.4556	-
y7(+1)	-	908.4876	-
y8(+1)	965.5142	965.5091	5.30
y9(+1)	-	1078.5932	-
y10(+1)	-	1206.6881	-
y11(+1)	1293.7213	1293.7202	0.84
y12(+1)	1422.7611	1422.7628	1.18
y13(+1)	1569.8345	1569.8312	2.11
y14(+1)	1684.8613	1684.8581	1.88
y15(+1)	1812.9504	1812.9531	1.48
y16(+1)	1944.0036	1943.9936	5.17
y16(+2)	972.5054	972.5004	5.17
y17(+1)	-	2057.0776	-
y18(+1)	-	2154.1304	-
y19(+1)	-	2282.2253	-

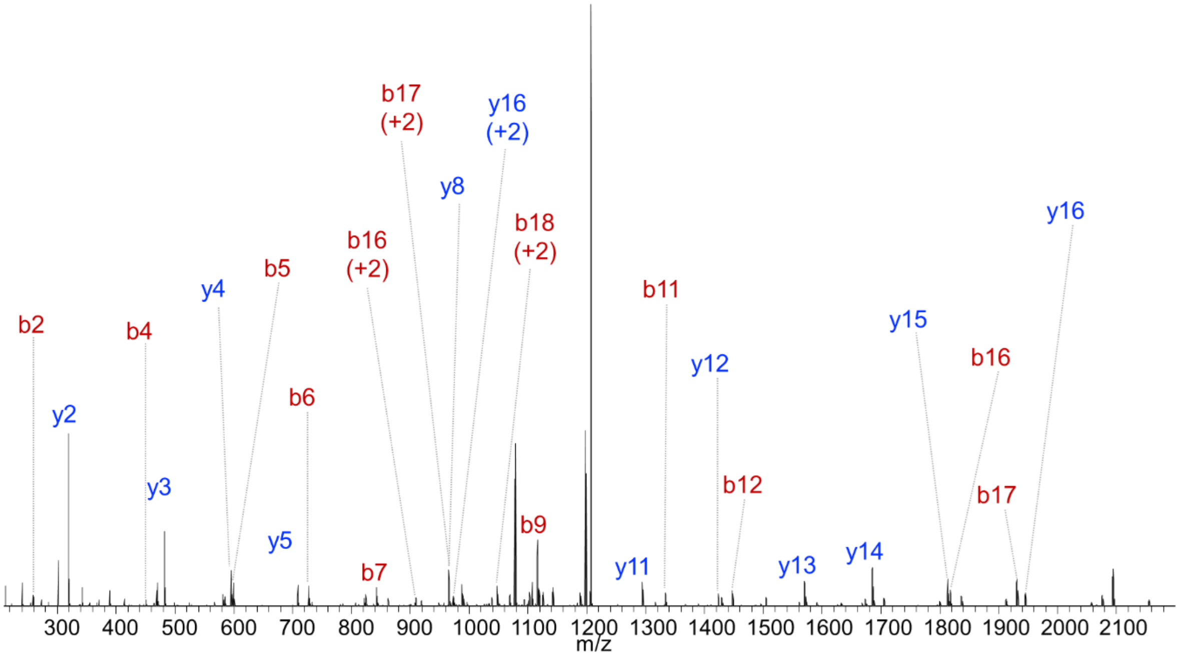

Figure S12. MS2 fragmentation of linear ArbA7-LLLY$_{87\text{-}106}$ substrate. Core peptide is highlighted in orange.

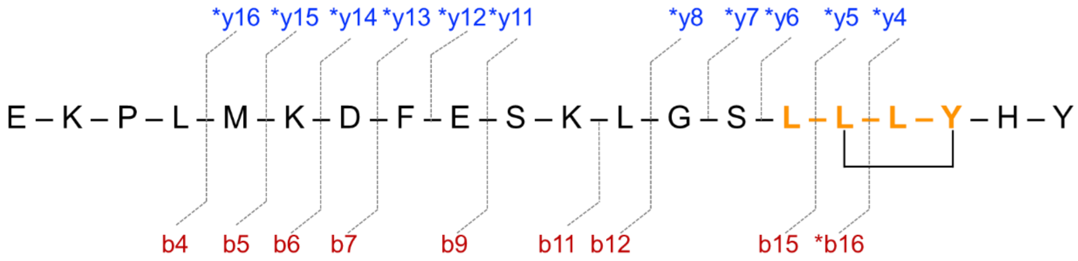

Fragment (z)	m (obs)	m (cal)	Δm (ppm)
b2(+1)	-	258.1448	-
b3(+1)	-	355.1976	-
b4(+1)	468.2820	468.2816	0.75
b5(+1)	599.3226	599.3221	0.77
b6(+1)	727.4168	727.4171	0.41
b7(+1)	842.4447	842.4440	0.78
b8(+1)	-	989.5124	-
b9(+1)	1118.5542	1118.5550	0.75
b10(+1)	-	1205.5871	-
b11(+1)	1333.6825	1333.6820	0.35
b12(+1)	1446.7698	1446.7661	2.56
b13(+1)	-	1503.7875	-
b14(+1)	-	1590.8196	-
b15(+1)	1703.9071	1703.9036	2.03
b16(+1)*	1814.9728	1814.9720	0.43
b17(+1)*	-	1928.0561	-

Fragment (z)	m (obs)	m (cal)	Δm (ppm)
y3(+1)	-	482.2034	-
y4(+1)	595.2883	595.2875	1.33
y5(+1)*	706.3559	706.3568	1.29
y6(+1)*	819.4411	819.4399	1.40
y7(+1)*	906.4728	906.4720	0.90
y8(+1)*	963.4923	963.4934	1.18
y9(+1)*	-	1076.5775	-
y10(+1)*	-	1204.6725	-
y11(+1)*	1291.7048	1291.7045	0.24
y12(+1)*	1420.7495	1420.7471	1.70
y13(+1)*	1567.8148	1567.8155	0.44
y14(+1)*	1682.8470	1682.8424	2.72
y15(+1)*	1810.9407	1810.9374	1.83
y16(+1)*	1941.9874	1941.9779	4.90
y17(+1)*	-	2055.0619	-
y18(+1)*	-	2152.1147	-
y19(+1)*	-	2280.2097	-

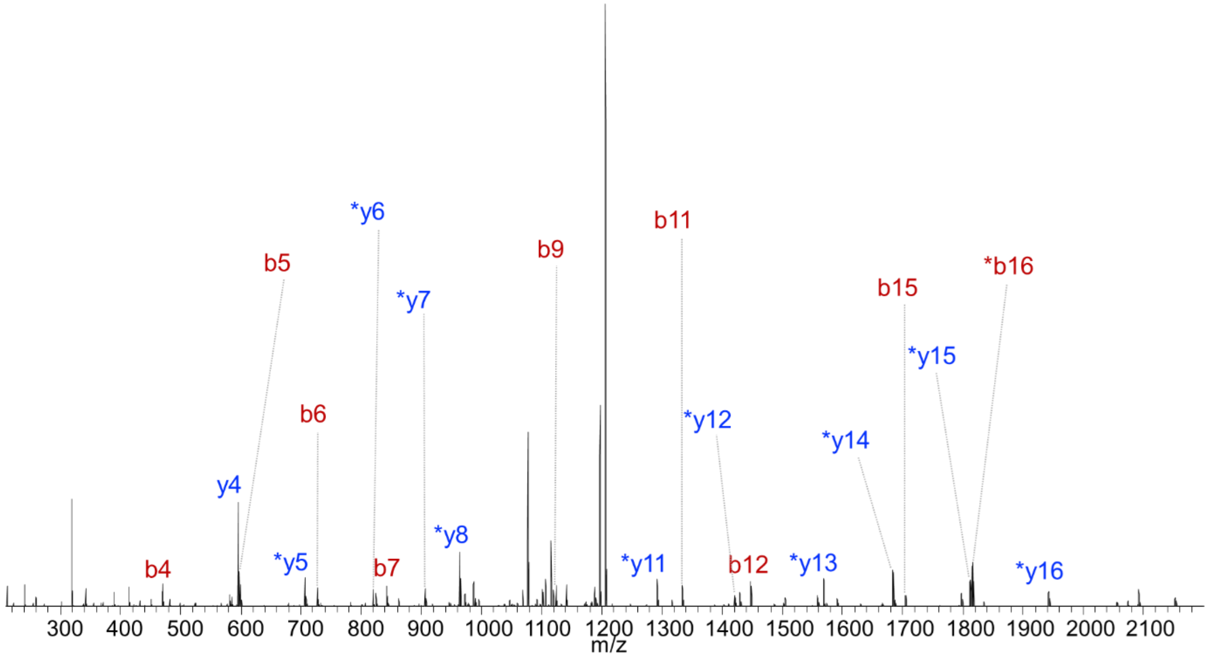

Figure S13. MS2 fragmentation of cyclic ArbA7-LLLY$_{87\text{-}106}$ product. Stars (*) indicate the loss of two hydrogens in the MS2 fragment ions. Core peptide is highlighted in orange.

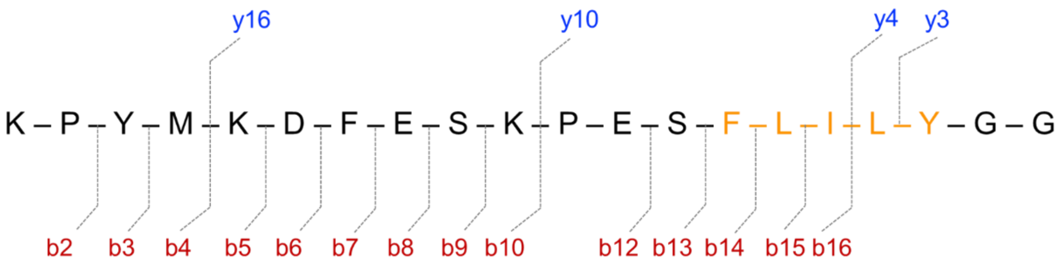

Fragment (z)	m (obs)	m (cal)	Δm (ppm)
b2(+1)	226.1548	226.1550	0.88
b3(+1)	389.2178	389.2183	1.36
b4(+1)	520.2588	520.2588	0
b5(+1)	648.3548	648.3538	1.57
b6(+1)	763.3814	763.3807	0.89
b7(+1)	910.4475	910.4491	1.79
b8(+1)	1039.4932	1039.4917	1.42
b9(+1)	1126.5231	1126.5238	0.58
b10(+1)	1254.6183	1254.6187	0.33
b11(+1)	-	1351.6715	-
b12(+1)	1480.7297	1480.7141	10.53
b13(+1)	1567.7380	1567.7461	5.17
b14(+1)	1714.8201	1714.8145	3.26
b15(+1)	1827.8966	1827.8986	1.07
b16(+1)	1940.9906	1940.9826	4.12
b17(+1)	-	2054.0667	-
b18(+1)	-	2217.1300	-
b19(+1)	-	2274.1515	-

Fragment (z)	m (obs)	m (cal)	Δm (ppm)
y2(+1)	-	133.0608	-
y3(+1)	296.1235	296.1241	2.13
y4(+1)	409.2077	409.2082	1.20
y5(+1)	-	522.2922	-
y6(+1)	-	635.3763	-
y7(+1)	-	782.4447	-
y8(+1)	-	869.4767	-
y9(+1)	-	998.5193	-
y10(+1)	1095.5735	1095.5721	1.28
y11(+1)	-	1223.6671	-
y12(+1)	-	1310.6991	-
y13(+1)	-	1439.7417	-
y14(+1)	-	1586.8101	-
y15(+1)	-	1701.8370	-
y16(+1)	1829.9163	1829.9320	8.57
y17(+1)	-	1960.9725	-
y18(+1)	-	2124.0358	-
y19(+1)	-	2221.0886	-

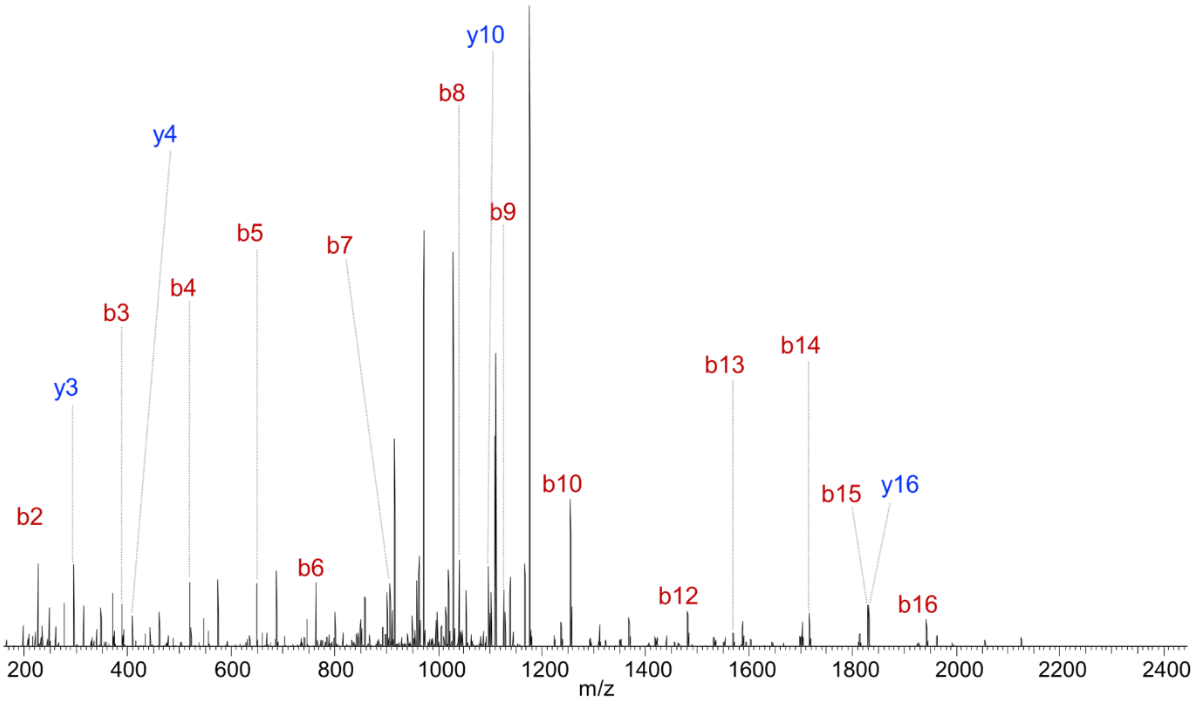

Figure S14. MS2 fragmentation of linear ArbA6-FLILY$_{168\text{-}187}$ substrate. Core peptide is highlighted in orange.

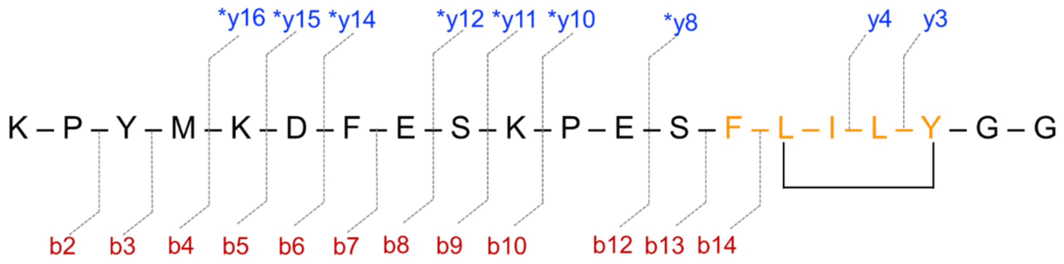

Fragment (z)	m (obs)	m (cal)	Δm (ppm)
b2(+1)	226.1548	226.1550	0.88
b3(+1)	389.2181	389.2183	0.59
b4(+1)	520.2593	520.2588	0.92
b5(+1)	648.3556	648.3538	2.81
b6(+1)	763.3815	763.3807	1.02
b7(+1)	910.4483	910.4491	0.91
b8(+1)	1039.4908	1039.4917	0.89
b9(+1)	1126.5238	1126.5238	0.04
b10(+1)	1254.6178	1254.6187	0.73
b11(+1)	-	1351.6715	-
b12(+1)	1480.7142	1480.7141	0.09
b13(+1)	1567.7518	1567.7461	3.63
b14(+1)	1714.8212	1714.8145	3.91
b15(+1)*	-	1825.8829	-
b16(+1)*	-	1938.9670	-
b17(+1)*	-	2052.0510	-
b18(+1)*	-	2215.1143	-
b19(+1)*	-	2272.1358	-

Fragment (z)	m (obs)	m (cal)	Δm (ppm)
y2(+1)	-	133.0608	-
y3(+1)	296.1238	296.1241	1.11
y4(+1)	409.2081	409.2082	0.24
y5(+1)	-	522.2922	-
y6(+1)*	-	633.3606	-
y7(+1)*	-	780.4291	-
y8(+1)*	867.4599	867.4611	1.38
y9(+1)*	-	996.5037	-
y10(+1)*	1093.5568	1093.5564	0.33
y11(+1)*	1221.6524	1221.6514	0.82
y12(+1)*	1308.6827	1308.6834	0.56
y13(+1)*	-	1437.7260	-
y14(+1)*	1584.7936	1584.7944	0.52
y15(+1)*	1699.8174	1699.8214	2.34
y16(+1)*	1827.9033	1827.9163	7.13
y17(+1)*	-	1958.9568	-
y18(+1)*	-	2122.0201	-
y19(+1)*	-	2219.0729	-

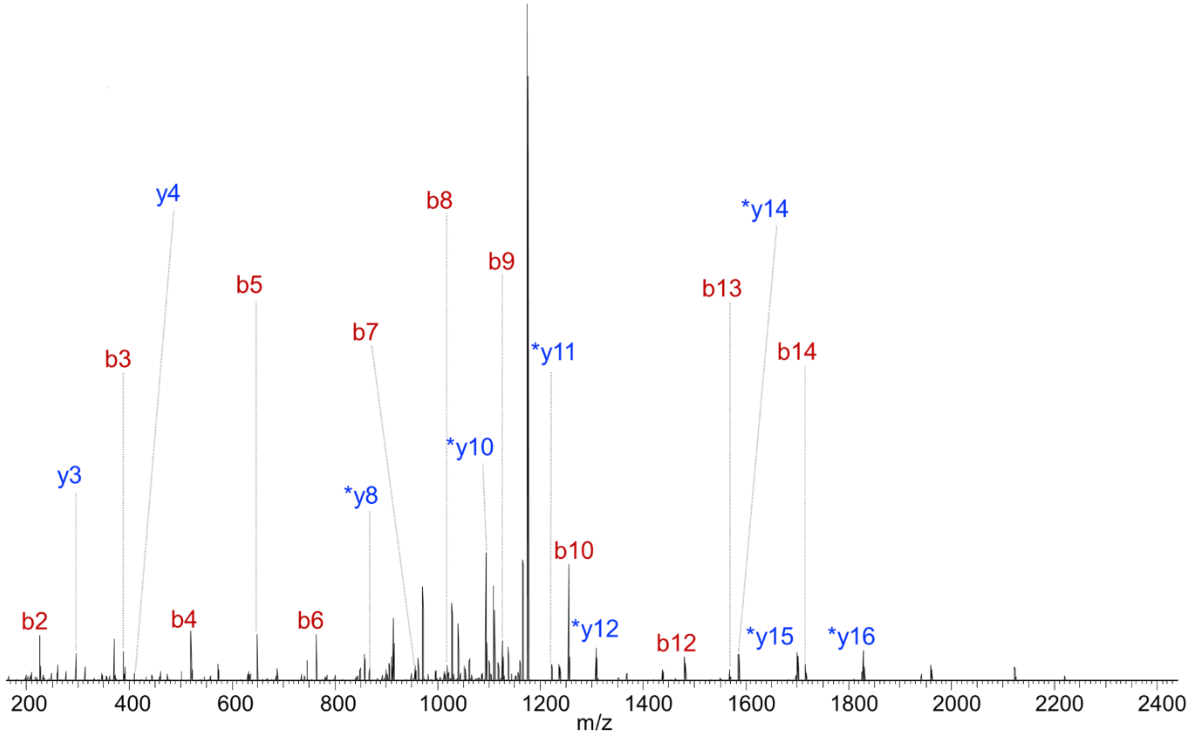

Figure S15. MS2 fragmentation of cyclic ArbA6-FLILY$_{168\text{-}187}$ product. Stars (*) indicate the loss of two hydrogens in the MS2 fragment ions. Core peptide is highlighted in orange.

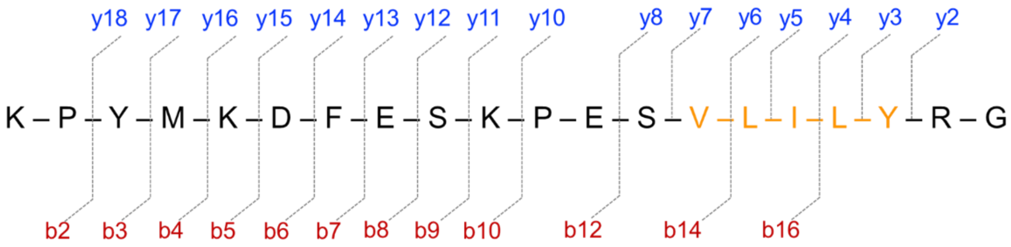

Fragment (z)	m (obs)	m (cal)	Δm (ppm)
b2(+1)	226.1547	226.1550	1.33
b3(+1)	389.2177	389.2183	1.62
b4(+1)	520.2588	520.2588	0
b5(+1)	648.3550	648.3538	1.88
b6(+1)	763.3816	763.3807	1.15
b7(+1)	910.4483	910.4491	0.91
b8(+1)	1039.4897	1039.4917	1.94
b9(+1)	1126.5225	1126.5238	1.11
b10(+1)	1254.6176	1254.6187	0.88
b11(+1)	-	1351.6715	-
b12(+1)	1480.7088	1480.7141	3.55
b13(+1)	-	1567.7461	-
b14(+1)	1666.8124	1666.8145	1.26
b15(+1)	-	1779.8986	-
b16(+1)	1892.9840	1892.9826	0.73
b17(+1)	-	2006.0667	-
b18(+1)	-	2169.1300	-
b19(+1)	-	2325.2311	-

Fragment (z)	m (obs)	m (cal)	Δm (ppm)
y2(+1)	232.1402	232.1404	1.08
y3(+1)	395.2035	395.2038	0.71
y4(+1)	508.2880	508.2878	0.31
y5(+1)	621.3721	621.3719	0.32
y6(+1)	734.4568	734.4560	1.14
y7(+1)	833.5259	833.5244	1.80
y8(+1)	920.5579	920.5564	1.63
y9(+1)	-	1049.5990	-
y10(+1)	1146.6521	1146.6517	0.31
y11(+1)	1274.7476	1274.7467	0.70
y12(+1)	1361.7787	1361.7787	0.03
y13(+1)	1490.8188	1490.8213	1.70
y14(+1)	1637.8868	1637.8897	1.79
y15(+1)	1752.9157	1752.9167	0.56
y16(+1)	1881.0164	1881.0116	2.53
y17(+1)	2012.0651	2012.0521	6.45
y18(+1)	2175.1255	2175.1155	4.62
y19(+1)	-	2272.1682	-

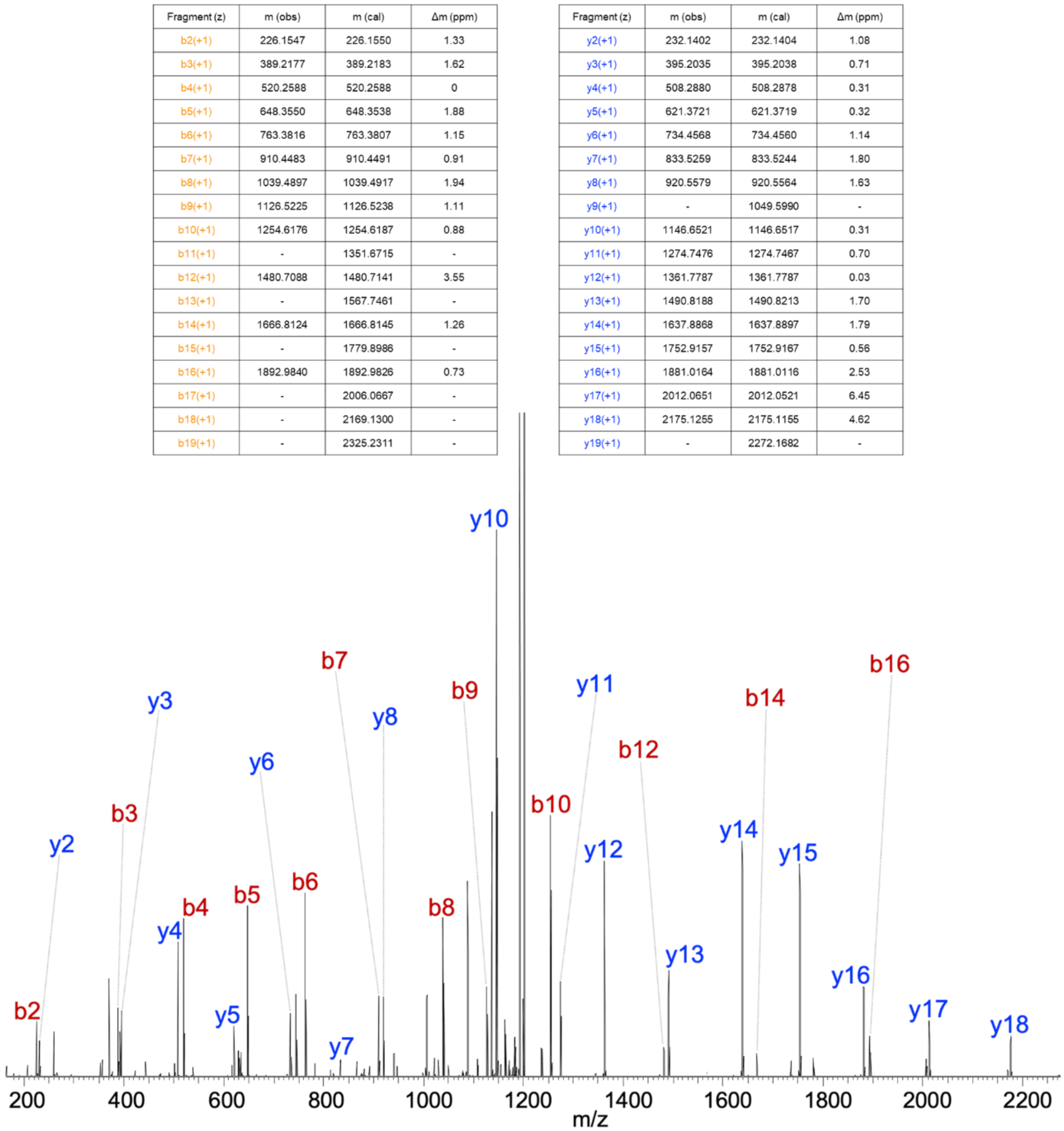

Figure S16. MS2 fragmentation of linear ArbA6-VLILY$_{112\text{-}131}$ substrate. Core peptide is highlighted in orange.

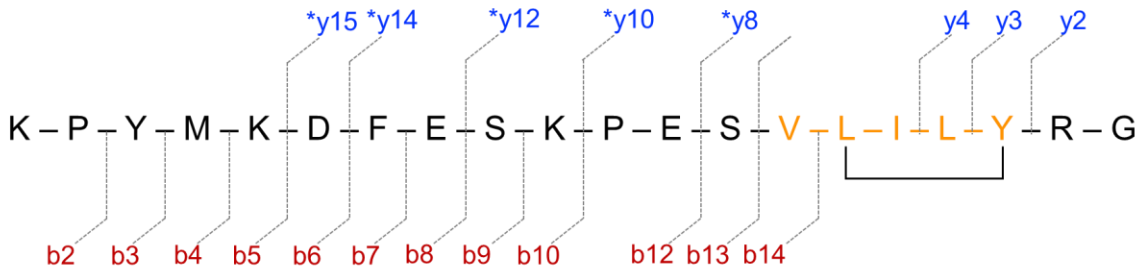

Fragment (z)	m (obs)	m (cal)	Δm (ppm)
b2(+1)	226.1547	226.1550	1.33
b3(+1)	389.2180	389.2183	0.85
b4(+1)	520.2590	520.2588	0.35
b5(+1)	648.3546	648.3538	1.26
b6(+1)	763.3814	763.3807	0.89
b7(+1)	910.4486	910.4491	0.58
b8(+1)	1039.4889	1039.4917	2.71
b9(+1)	1126.5235	1126.5238	0.22
b10(+1)	1254.6176	1254.6187	0.88
b11(+1)	-	1351.6715	-
b12(+1)	1480.7055	1480.7141	5.78
b13(+1)	1567.7468	1567.7461	0.45
b14(+1)	1666.8163	1666.8145	1.08
b15(+1)*	-	1777.8829	-
b16(+1)*	-	1890.9670	-
b17(+1)*	-	2004.0510	-
b18(+1)*	-	2167.1143	-
b19(+1)*	-	2323.2155	-

Fragment (z)	m (obs)	m (cal)	Δm (ppm)
y2(+1)	232.1401	232.1404	1.51
y3(+1)	395.2039	395.2038	0.30
y4(+1)	508.2879	508.2878	0.12
y5(+1)	-	621.3719	-
y6(+1)*	-	732.4403	-
y7(+1)*	-	831.5087	-
y8(+1)*	918.5455	918.5407	5.22
y9(+1)*	-	1047.5833	-
y10(+1)*	1144.6346	1144.6361	1.30
y11(+1)*	-	1272.7310	-
y12(+1)*	1359.7660	1359.7631	2.15
y13(+1)*	-	1488.8057	-
y14(+1)*	1635.8701	1635.8741	2.43
y15(+1)*	1750.9053	1750.9010	2.44
y16(+1)*	-	1878.9960	-
y17(+1)*	-	2010.0365	-
y18(+1)*	-	2173.0998	-
y19(+1)*	-	2270.1526	-

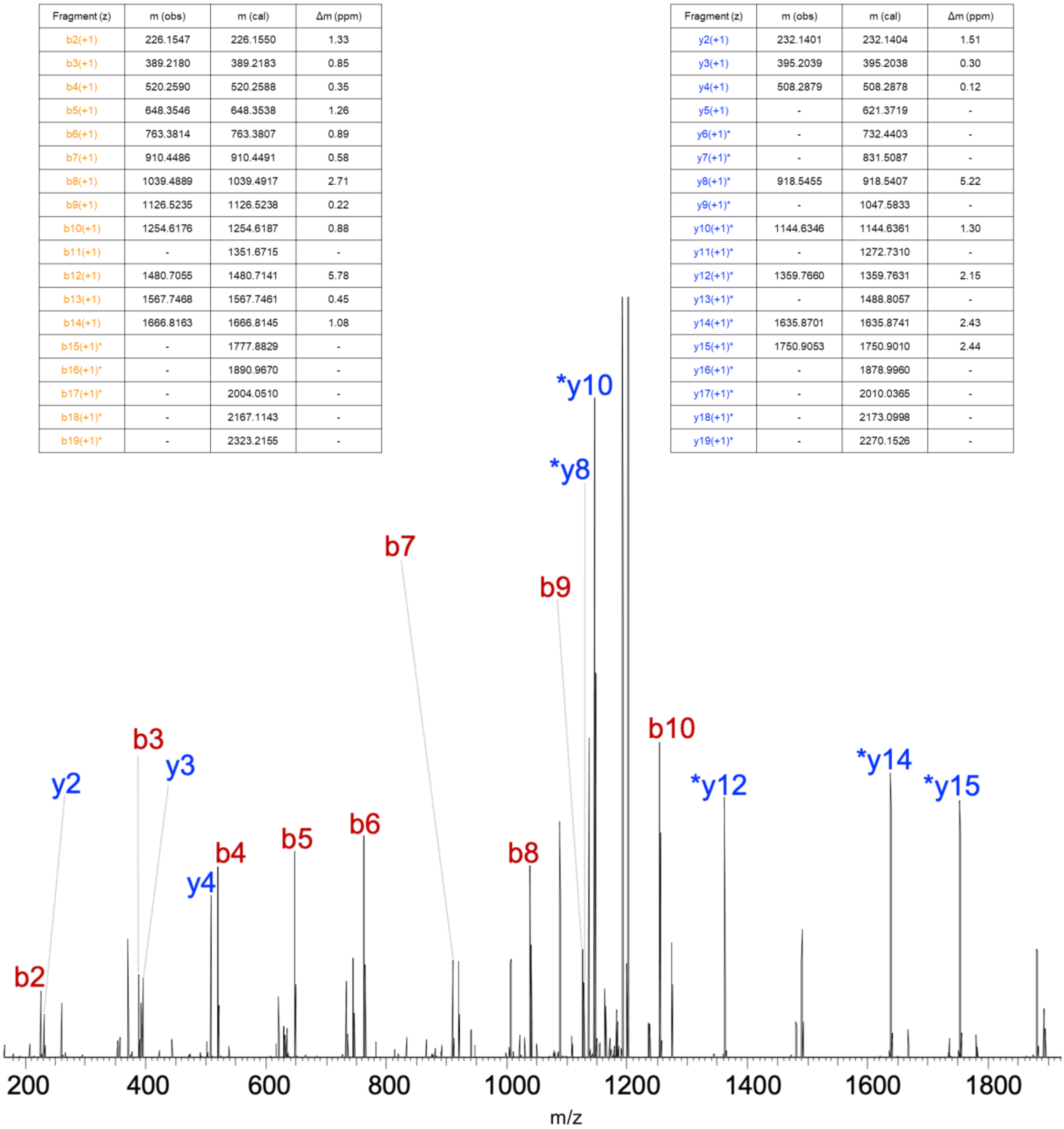

Figure S17. MS2 fragmentation of cyclic ArbA6-VLILY$_{112\text{-}131}$ product. Stars (*) indicate the loss of two hydrogens in the MS2 fragment ions. Core peptide is highlighted in orange.

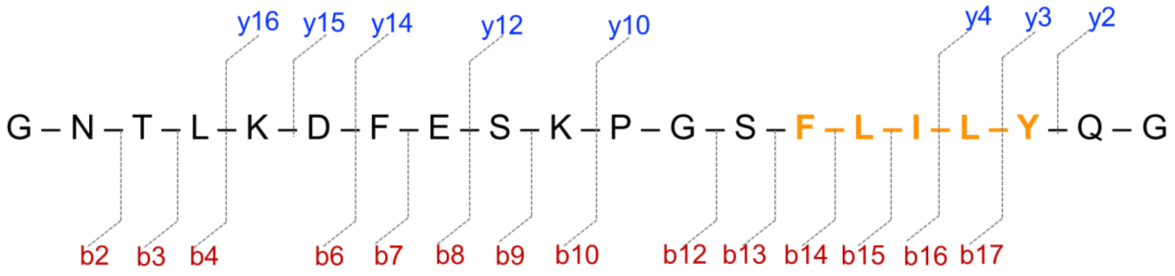

Fragment (z)	m (obs)	m (cal)	Δm (ppm)
b2(+1)	172.0715	172.0717	0.99
b3(+1)	273.1193	273.1193	0
b4(+1)	386.2037	386.2034	0.75
b5(+1)	-	514.2984	-
b6(+1)	629.3268	629.3253	2.37
b7(+1)	776.3953	776.3937	2.04
b8(+1)	905.4369	905.4363	0.65
b9(+1)	992.4702	992.4683	1.87
b10(+1)	1120.5639	1120.5633	0.54
b11(+1)	-	1217.6161	-
b12(+1)	1274.6349	1274.6375	2.04
b13(+1)	-	1361.6696	-
b13(+2)	681.3427	681.3384	6.31
b14(+1)	1508.7335	1508.7380	2.98
b14(+2)	754.8773	754.8726	6.23
b15(+1)	1621.8225	1621.8220	0.30
b16(+1)	1734.9037	1734.9061	1.37
b17(+1)	1847.9894	1847.9901	0.40
b18(+1)	-	2011.0535	-
b19(+1)	-	2139.1121	-

Fragment (z)	m (obs)	m (cal)	Δm (ppm)
y2(+1)	204.0978	204.0979	0.59
y3(+1)	367.1613	367.1612	0.14
y4(+1)	480.2450	480.2453	0.65
y5(+1)	-	593.3293	-
y6(+1)	-	706.4134	-
y7(+1)	-	853.4818	-
y8(+1)	-	940.5139	-
y9(+1)	-	997.5353	-
y10(+1)	1094.5886	1094.5881	0.47
y11(+1)	-	1222.6831	-
y12(+1)	1309.7153	1309.7151	0.17
y13(+1)	-	1438.7577	-
y14(+1)	1585.8224	1585.8261	2.32
y15(+1)	1700.8556	1700.8530	1.52
y16(+1)	1828.9448	1828.9480	1.74
y16(+2)	914.9783	914.9776	0.76
y17(+1)	-	1942.0320	-
y18(+1)	-	2043.0797	-
y19(+1)	-	2157.1226	-

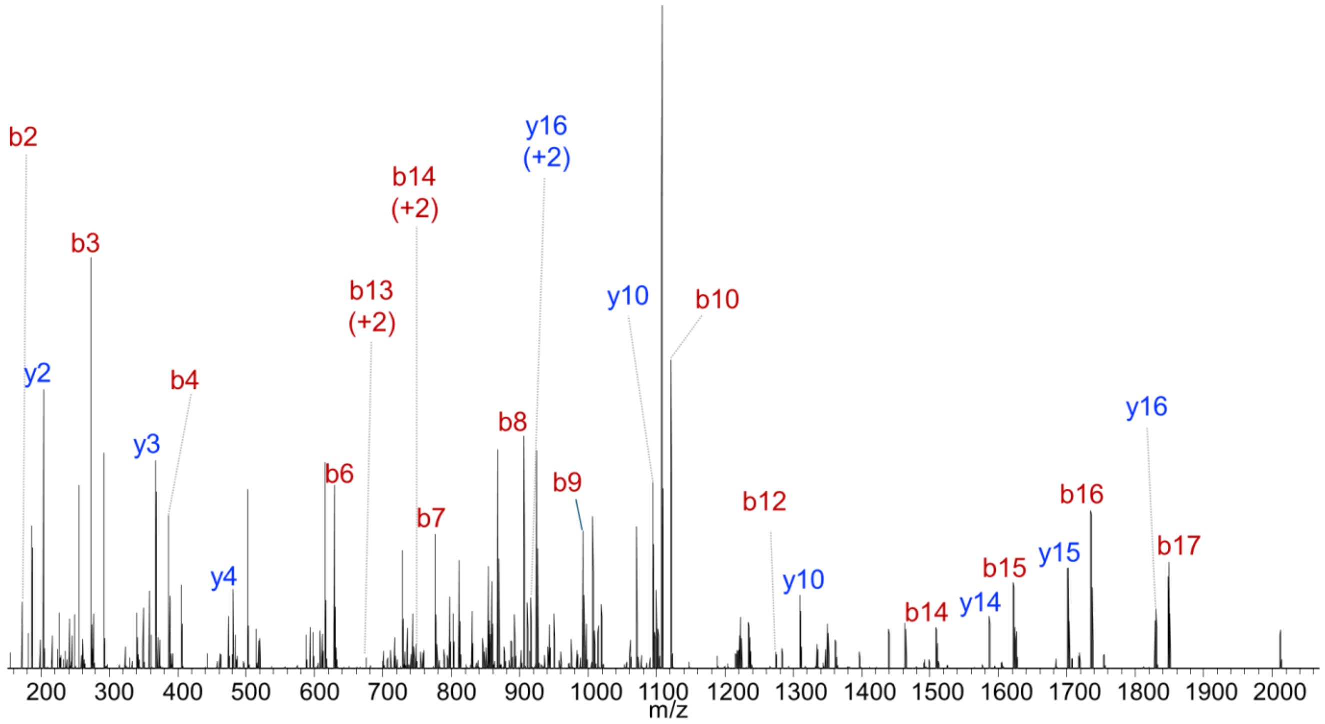

Figure S18. MS2 fragmentation of linear ArbA2-FLILY$_{58\text{-}77}$ substrate. Core peptide is highlighted in orange.

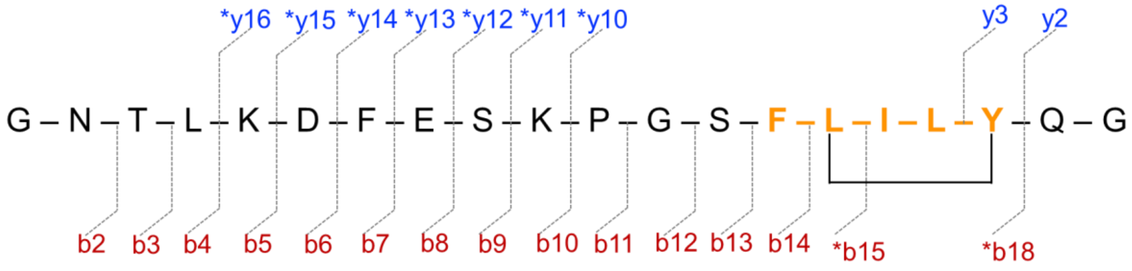

Fragment (z)	m (obs)	m (cal)	Δm (ppm)
b2(+1)	172.0716	172.0717	0.41
b3(+1)	273.1194	273.1193	0.18
b4(+1)	386.2038	386.2034	1.01
b5(+1)	514.2995	514.2984	2.20
b6(+1)	629.3241	629.3253	1.92
b7(+1)	776.3955	776.3937	2.29
b8(+1)	905.4383	905.4363	2.20
b9(+1)	992.4666	992.4683	1.75
b10(+1)	1120.5639	1120.5633	0.54
b11(+1)	1217.6210	1217.6161	4.02
b12(+1)	1274.6377	1274.6375	0.16
b13(+1)	1361.6757	1361.6696	4.52
b14(+1)	1508.7403	1508.7380	1.55
b15(+1)*	1619.8071	1619.8064	0.46
b16(+1)*	-	1732.8904	-
b17(+1)*	-	1847.9902	-
b18(+1)*	2009.0377	2009.0378	0.05
b19(+1)*	-	2137.0964	-

Fragment (z)	m (obs)	m (cal)	Δm (ppm)
y2(+1)	204.0979	204.0979	0
y3(+1)	367.1614	367.1612	0.41
y4(+1)	-	480.2453	-
y5(+1)	-	593.3293	-
y6(+1)*	-	704.3978	-
y7(+1)*	-	851.4662	-
y8(+1)*	-	938.4982	-
y9(+1)*	-	995.5197	-
y10(+1)*	1092.5724	1092.5724	0.03
y11(+1)*	1220.6630	1220.6674	3.60
y12(+1)*	1307.7003	1307.6994	0.67
y13(+1)*	1436.7425	1436.7420	0.34
y14(+1)*	1583.8119	1583.8104	0.93
y15(+1)*	1698.8365	1698.8374	0.51
y16(+1)*	1826.9356	1826.9323	1.80
y17(+1)*	-	1940.0164	-
y18(+1)*	-	2041.0641	-
y19(+1)*	-	2155.1070	-

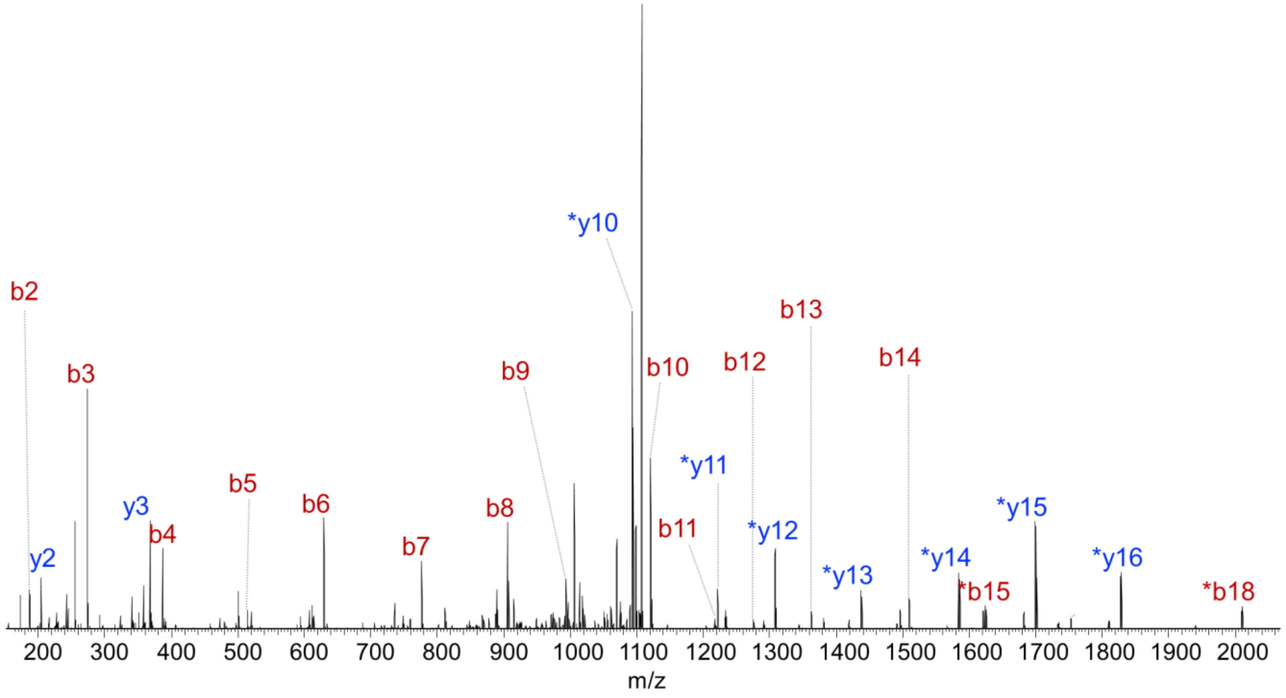

Figure S19. MS2 fragmentation of cyclic ArbA2-FLILY$_{58\text{-}77}$ product. Stars (*) indicate the loss of two hydrogens in the MS2 fragment ions. Core peptide is highlighted in orange.

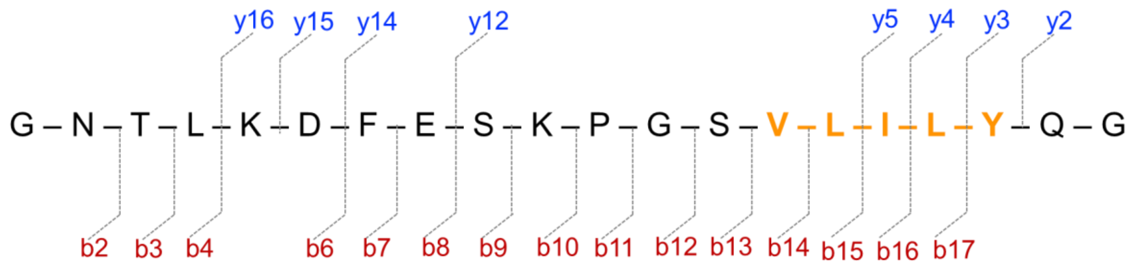

Fragment (z)	m (obs)	m (cal)	Δm (ppm)
b2(+1)	172.0717	172.0717	0
b3(+1)	273.1193	273.1193	0
b4(+1)	386.2039	386.2034	1.27
b5(+1)	-	514.2984	-
b6(+1)	629.3264	629.3253	1.73
b7(+1)	776.3941	776.3937	0.49
b8(+1)	905.4356	905.4363	0.78
b9(+1)	992.4669	992.4683	1.45
b10(+1)	1120.5633	1120.5633	0
b11(+1)	1217.6171	1217.6161	0.82
b12(+1)	1274.6398	1274.6375	1.80
b13(+1)	1361.6655	1361.6696	3.01
b14(+1)	1460.7456	1460.7380	5.23
b15(+1)	1573.8212	1573.8220	0.52
b16(+1)	1686.9114	1686.9061	3.15
b17(+1)	1799.9897	1799.9901	0.24
b18(+1)	-	1963.0535	-
b19(+1)	-	2091.1121	-

Fragment (z)	m (obs)	m (cal)	Δm (ppm)
y2(+1)	204.0978	204.0979	0.59
y3(+1)	367.1613	367.1612	0.14
y4(+1)	480.2453	480.2453	0
y5(+1)	593.3318	593.3293	4.10
y6(+1)	-	706.4134	-
y7(+1)	-	805.4818	-
y8(+1)	-	892.5138	-
y9(+1)	-	949.5353	-
y10(+1)	-	1046.5881	-
y11(+1)	-	1174.6830	-
y12(+1)	1261.7094	1261.7151	4.50
y13(+1)	-	1390.7577	-
y14(+1)	1537.8316	1537.8261	3.59
y15(+1)	1652.8488	1652.8530	2.55
y16(+1)	1780.9532	1780.9480	2.93
y17(+1)	-	1894.0320	-
y18(+1)	-	1995.0797	-
y19(+1)	-	2109.1226	-

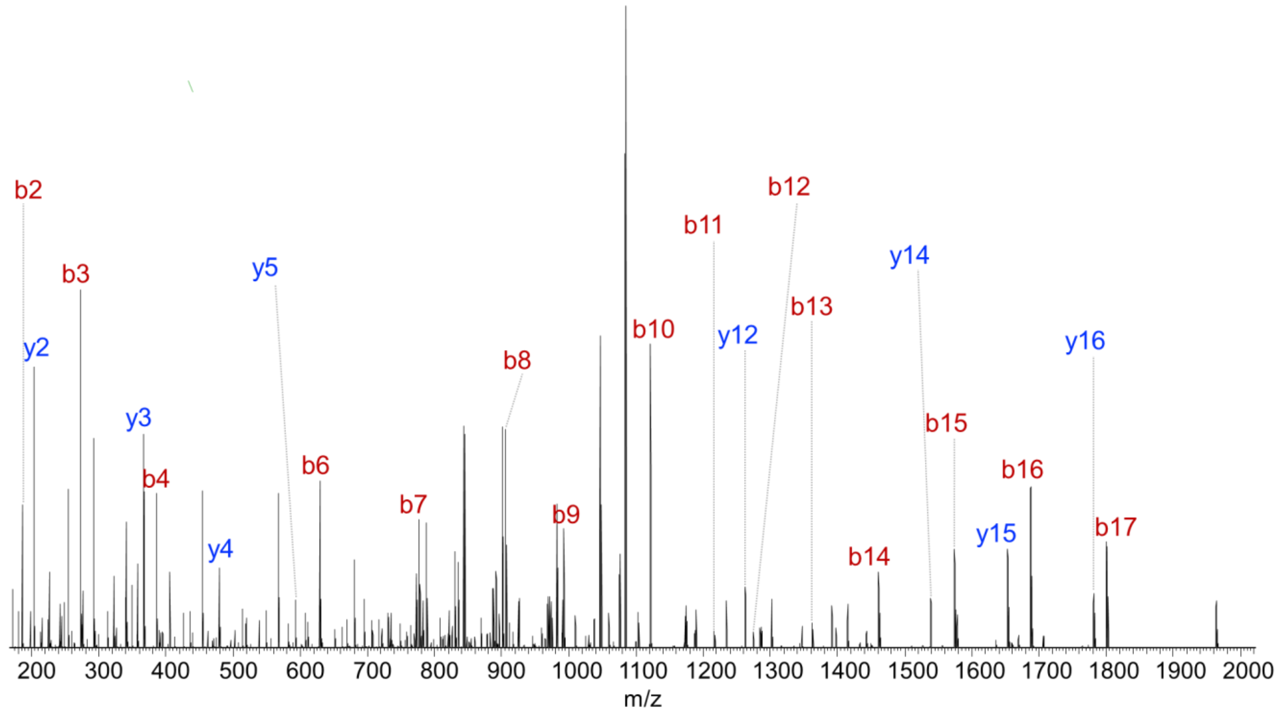

Figure S20. MS2 fragmentation of linear ArbA2-VLILY$_{58-77}$ substrate. Core peptide is highlighted in orange.

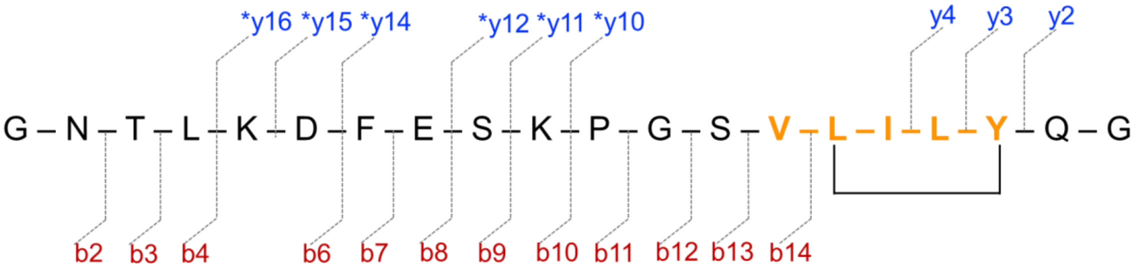

Fragment (z)	m (obs)	m (cal)	Δm (ppm)
b2(+1)	172.0715	172.0717	0.99
b3(+1)	273.1193	273.1193	0
b4(+1)	386.2037	386.2034	0.75
b5(+1)	-	514.2984	-
b6(+1)	629.3238	629.3253	2.40
b7(+1)	776.3956	776.3937	2.42
b8(+1)	905.4365	905.4363	0.21
b9(+1)	992.4672	992.4684	1.15
b10(+1)	1120.5638	1120.5633	0.45
b11(+1)	1217.6207	1217.6161	3.78
b12(+1)	1274.6338	1274.6375	2.90
b13(+1)	1361.6693	1361.6696	0.22
b14(+1)	1460.7388	1460.7380	0.58
b15(+1)*	-	1571.8064	-
b16(+1)*	-	1684.8904	-
b17(+1)*	-	1797.9745	-
b18(+1)*	-	1961.0378	-
b19(+1)*	-	2089.0964	-

Fragment (z)	m (obs)	m (cal)	Δm (ppm)
y2(+1)	204.0978	204.0979	0.59
y3(+1)	367.1614	367.1612	0.41
y4(+1)	480.2447	480.2453	1.27
y5(+1)	-	593.3294	-
y6(+1)*	-	704.3978	-
y7(+1)*	-	803.4662	-
y8(+1)*	-	890.4982	-
y9(+1)*	-	947.5197	-
y10(+1)*	1044.5708	1044.5724	1.56
y11(+1)*	1172.6612	1172.6674	5.28
y12(+1)*	1259.6971	1259.6994	1.84
y13(+1)*	-	1388.7420	-
y14(+1)*	1535.8132	1535.8104	1.81
y15(+1)*	1650.8451	1650.8374	4.69
y16(+1)*	1778.9290	1778.9323	1.87
y17(+1)*	-	1892.0164	-
y18(+1)*	-	1993.0641	-
y19(+1)*	-	2107.1070	-

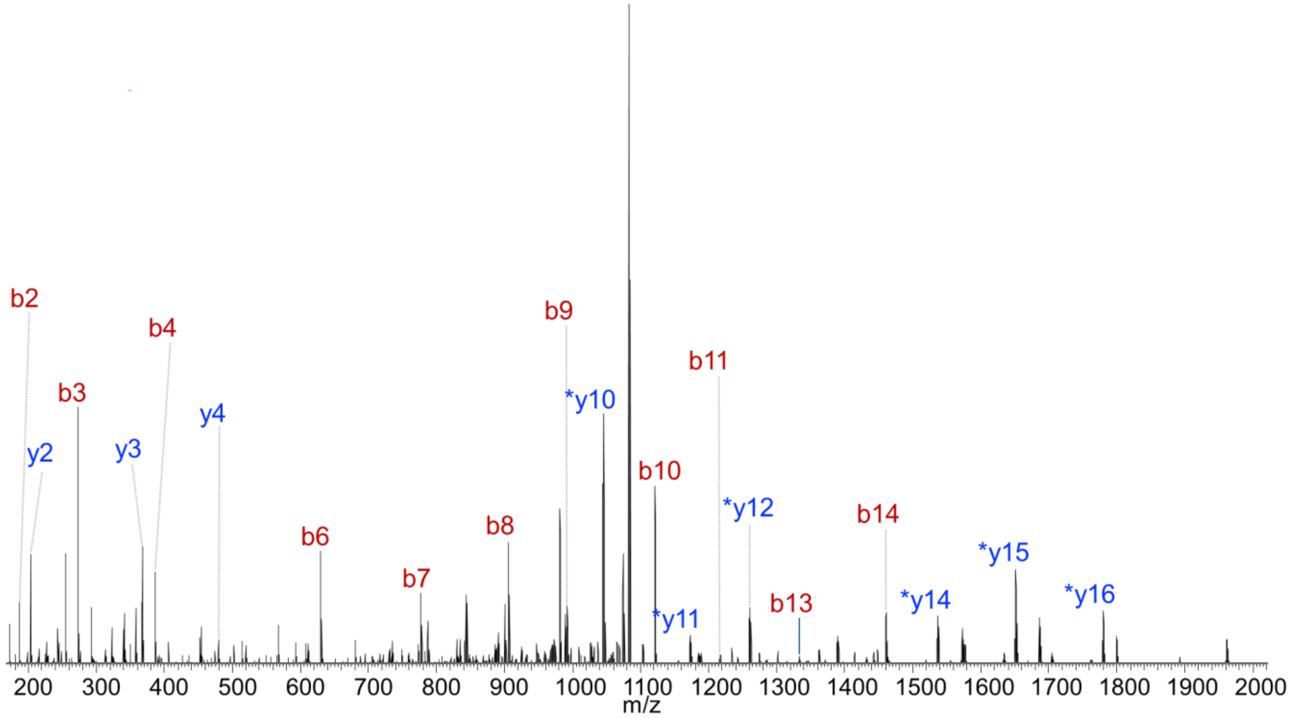

Figure S21. MS2 fragmentation of the linear ArbA2-VLILY$_{58\text{-}77}$ product. Stars (*) indicate the loss of two hydrogens in the MS2 fragment ions. Core peptide is highlighted in orange.

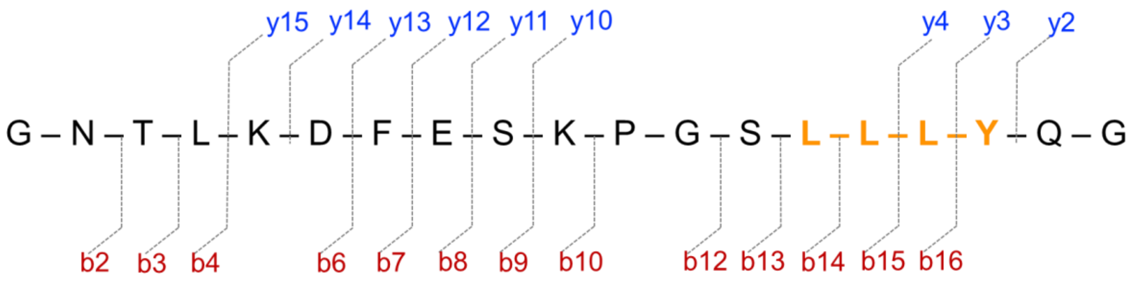

Fragment (z)	m (obs)	m (cal)	Δm (ppm)
b2(+1)	172.0715	172.0717	0.99
b3(+1)	273.1195	273.1193	0.55
b4(+1)	386.2038	386.2034	1.01
b5(+1)	-	514.2984	-
b6(+1)	629.3261	629.3253	1.26
b7(+1)	776.3959	776.3937	2.81
b8(+1)	905.4361	905.4363	0.23
b9(+1)	992.4660	992.4683	2.36
b10(+1)	1120.5638	1120.5633	0.45
b11(+1)	-	1217.6161	-
b12(+1)	1274.6292	1274.6375	6.51
b13(+1)	1361.6695	1361.6696	0.07
b14(+1)	1474.7519	1474.7536	1.16
b15(+1)	1587.8384	1587.8377	0.46
b16(+1)	1700.9221	1700.9218	0.22
b17(+1)	-	1863.9851	-
b18(+1)	-	1992.0437	-

Fragment (z)	m (obs)	m (cal)	Δm (ppm)
y2(+1)	204.0979	204.0979	0
y3(+1)	367.1617	367.1612	1.23
y4(+1)	480.2455	480.2453	0.40
y5(+1)	-	593.3293	-
y6(+1)	-	706.4134	-
y7(+1)	-	793.4454	-
y8(+1)	-	850.4669	-
y9(+1)	947.5203	947.5197	0.65
y10(+1)	1075.6131	1075.6146	1.43
y11(+1)	1162.6456	1162.6467	0.92
y12(+1)	1291.6861	1291.6893	2.45
y13(+1)	1438.7543	1438.7577	2.34
y14(+1)	1553.7823	1553.7846	1.49
y15(+1)	1681.8798	1681.8796	0.14
y16(+1)	-	1794.9636	-
y17(+1)	-	1896.0113	-
y18(+1)	-	2010.0542	-

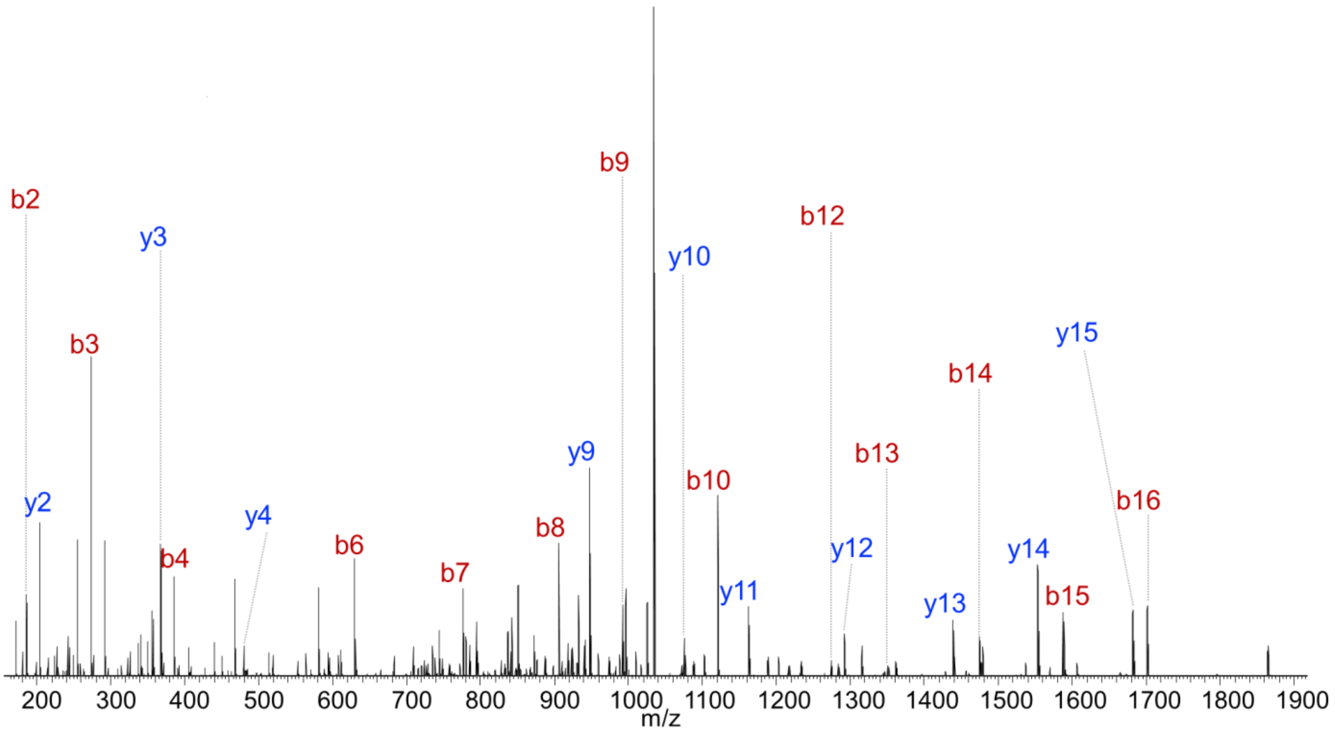

Figure S22. MS2 fragmentation of the linear ArbA2-LLLY$_{58\text{-}77}$ substrate. Core peptide is highlighted in orange.

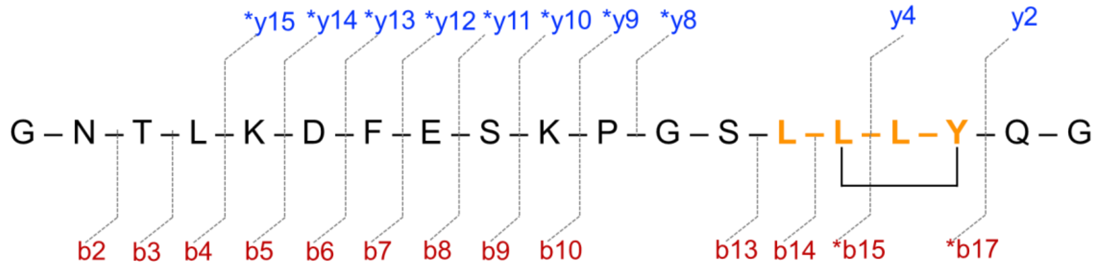

Fragment (z)	m (obs)	m (cal)	Δm (ppm)
b2(+1)	172.0715	172.0717	0.99
b3(+1)	273.1195	273.1193	0.55
b4(+1)	386.2038	386.2034	1.01
b5(+1)	514.3000	514.2984	3.17
b6(+1)	629.3253	629.3253	0.02
b7(+1)	776.3952	776.3937	1.91
b8(+1)	905.4360	905.4363	0.34
b9(+1)	992.4665	992.4683	1.85
b10(+1)	1120.5640	1120.5633	0.62
b11(+1)	-	1217.6161	-
b12(+1)	-	1274.6375	-
b13(+1)	1361.6766	1361.6696	5.18
b14(+1)	1474.7532	1474.7536	0.28
b15(+1)*	1585.8189	1585.8220	1.96
b16(+1)*	-	1698.9061	-
b17(+1)*	1861.9673	1861.9694	1.13
b18(+1)*	-	1990.0280	-

Fragment (z)	m (obs)	m (cal)	Δm (ppm)
y2(+1)	204.0979	204.0979	0
y3(+1)	-	367.1612	-
y4(+1)	480.2452	480.2453	0.23
y5(+1)*	-	591.3137	-
y6(+1)*	-	704.3978	-
y7(+1)*	-	791.4298	-
y8(+1)*	848.4499	848.4513	1.60
y9(+1)*	945.5045	945.5040	0.51
y10(+1)*	1073.5949	1073.5990	3.80
y11(+1)*	1160.6321	1160.6310	0.94
y12(+1)*	1289.6717	1289.6736	1.47
y13(+1)*	1436.7418	1436.7420	015
y14(+1)*	1551.7665	1551.7690	1.58
y15(+1)*	1679.8619	1679.8639	1.20
y16(+1)*	-	1792.9480	-
y17(+1)*	-	1893.9957	-
y18(+1)*	-	2008.0386	-

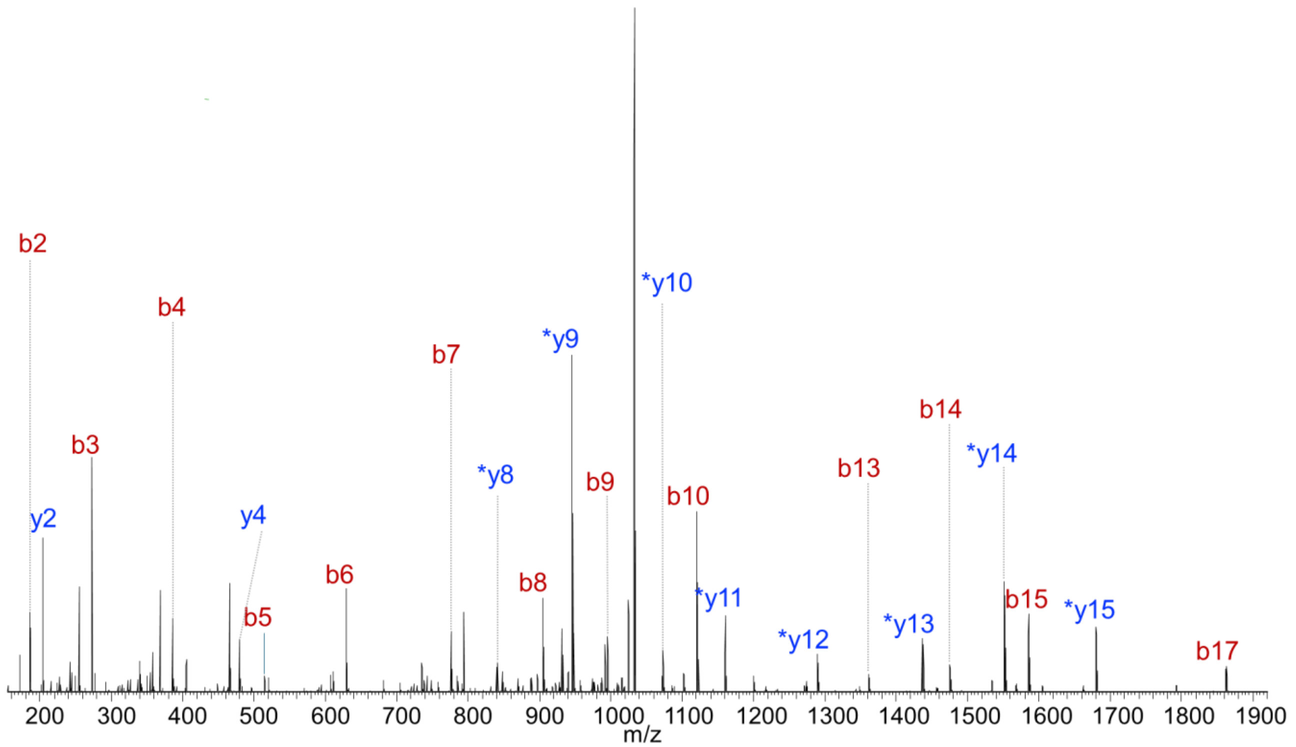

Figure S23. MS2 fragmentation of the cyclic ArbA2-LLLY$_{58\text{-}77}$ product. Stars (*) indicate the loss of two hydrogens in the MS2 fragment ions. Core peptide is highlighted in orange.

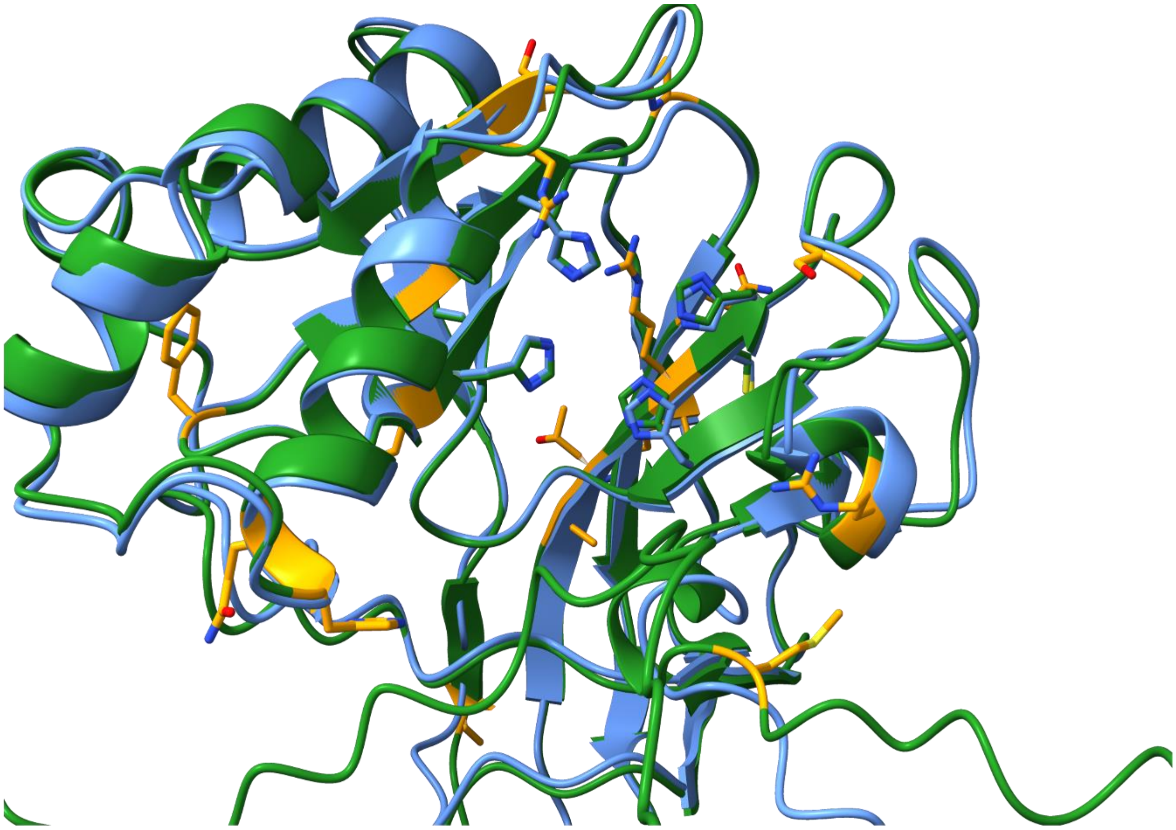

```
ArbB2_75-320   ------------------------------------------MHDPSLSSSHMMHQIDPR
ArbB3_23-288   MDIIPKAKDSGTNAIRLQGMDANNPHRNDKTHHVAHVHEKKSMHDPSLSSSHMMHQMDPR
                                                         **************:***

ArbB2_75-320   ATVFFVLDDLKLGKTLSILFPDGDPSPLSSPYLWPREQADAIPFSLAKLPQILQHFSFPQ
ArbB3_23-288   ATVFFVLDDLKLGKTLSILFPDGDPSPLSSPYLWPREQADAIPFSLAKLPQILQHFSFPQ
               ************************************************************

ArbB2_75-320   GSRKAQVMEHALRACETKPMKGEPKACATSYESLVDFARKILGLNTDIEVLSTHRLTKSN
ArbB3_23-288   GSHQAQVMEHTLRACETKPMKGESRACATSYESLVDFARKILGLNTDIEVLSTHRLAKSN
               **::******:************.:*******************************:***

ArbB2_75-320   AARLQNYTITEAPERISTLKMVGCHTMPYPYIVFYCHYQQGDNRLYRTVLSGENGDRVEG
ArbB3_23-288   AARLQNYTITEAPKRISTLKMVGCHNMPYPFIVFGCHYQPGDNNLYRTVLSGENGDRVEA
               *************:***********.****:*** **** ***.***************.

ArbB2_75-320   LAICHMDTSQWNHDHVSFQVLGIEPGTAPVCHFFPAEDFVLVPSTSSI
ArbB3_23-288   TARCHMDTSQWSHDHVSFRVLGIEPGTAPVCHFFPAEDFVLVPSTSSI
                * ********.******:*****************************
```

Figure S24. A) Superimposed models of ArbB2 (blue) and ArbB3 (green) with the differing amino acids shown in orange to show their location in relation to the active site. B) Sequence alignment of ArbB2 and ArbB3 with all the differences in the sequence highlighted in yellow

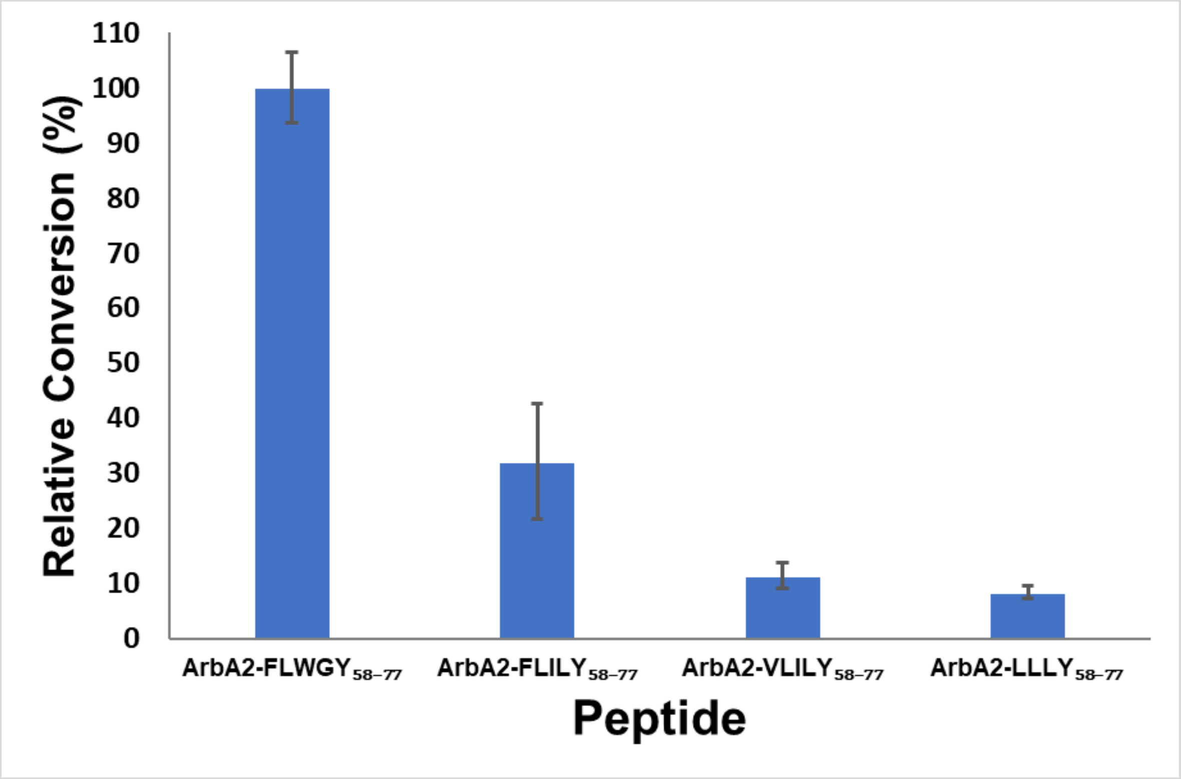

Figure S25. ArbB3 substrate scope assay. Assays were performed in citrate-phosphate buffer (pH 8.0) in a stoichiometric ratio of 1:150 enzyme/substrate and quenched after 60 minutes. Error bars indicated the standard deviation of three trials.

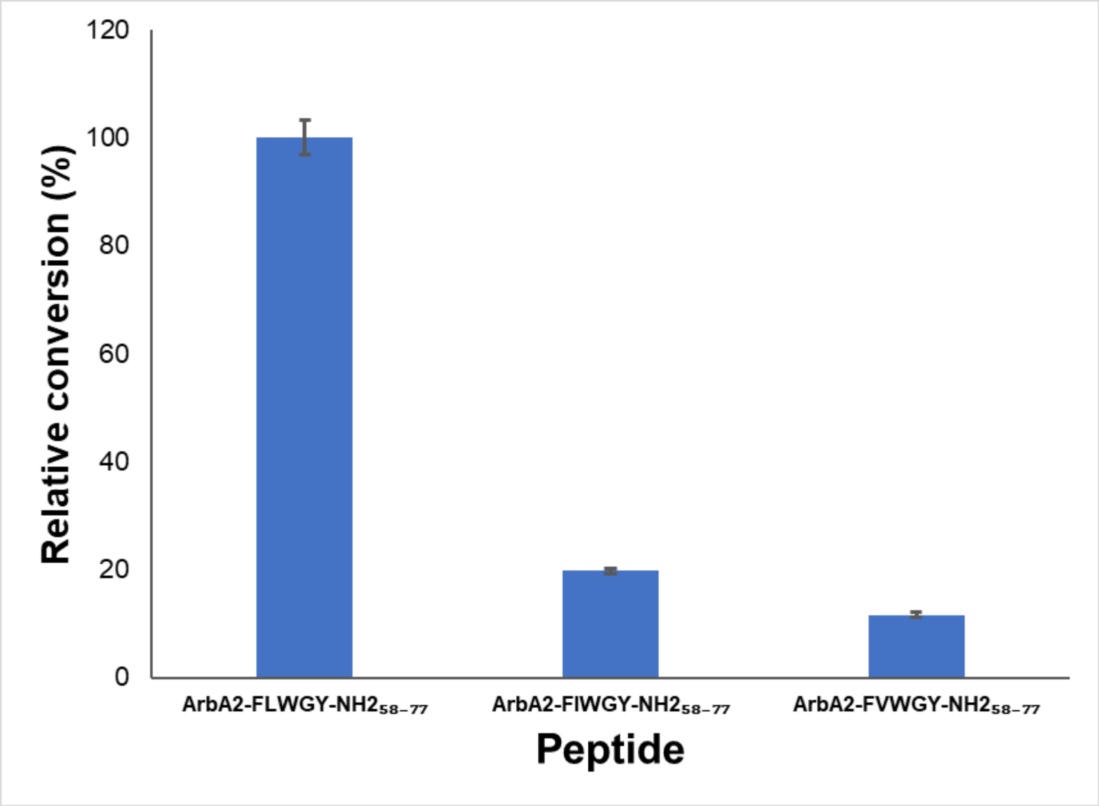

Figure S26. Modification of alternative core peptides. Assays were performed in citrate-phosphate buffer (pH 8.0), in a ratio of 1:150 enzyme/substrate, at room temperature, and quenched after 60 minutes. A) Relative conversion (%). Error bars indicated the standard deviation of three trials.

Note: These assays were completed with C-terminal amidated peptides instead of a carboxy terminus. This had no significant impact on conversion levels of the native F-L-W-G-Y core.

A

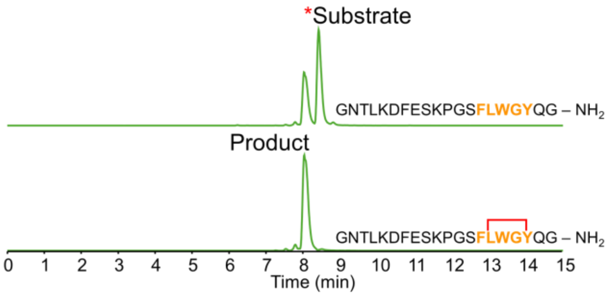

B

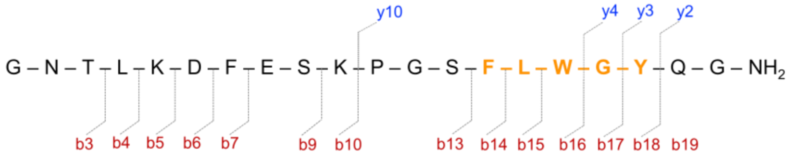

Fragment (z)	m (obs)	m (cal)	Δm (ppm)
b2(+1)	-	172.0717	-
b3(+1)	273.1193	273.1188	2.01
b4(+1)	386.2034	386.204	1.53
b5(+1)	514.2984	514.2967	3.25
b6(+1)	629.3253	629.3256	0.46
b7(+1)	776.3937	776.3958	2.68
b8(+1)	-	905.4363	-
b9(+1)	992.4683	992.47	1.67
b10(+1)	1120.5633	1120.5625	0.71
b11(+2)	-	609.3117	-
b12(+2)	-	637.8224	-
b13(+2)	681.3384	681.3392	1.15
b14(+2)	754.8726	754.8754	3.68
b15(+2)	811.4146	811.4136	1.29
b16(+2)	904.4543	904.4557	1.54
b17(+2)	932.9650	932.9646	0.47
b18(+2)	1014.4967	1014.4928	3.84
b19(+2)	1078.5260	1078.5289	2.70

Fragment (z)	m (obs)	m (cal)	Δm (ppm)
y2(+1)	203.1139	203.1135	1.82
y3(+1)	366.1772	366.1775	0.82
y4(+1)	423.1987	423.1986	0.14
y5(+1)	-	609.2780	-
y6(+1)	-	722.3620	-
y7(+1)	-	869.4304	-
y8(+1)	-	956.4625	-
y9(+1)	-	1013.4839	-
y10(+1)	1110.5367	1110.5359	0.71
y11(+2)	-	619.8195	-
y12(+2)	-	663.3355	-
y13(+2)	-	727.8568	-
y14(+2)	-	801.3910	-
y15(+2)	-	858.9045	-
y16(+2)	-	922.9519	-
y17(+2)	-	979.4940	-
y18(+2)	-	1030.0178	-
y19(+2)	-	1087.0393	-

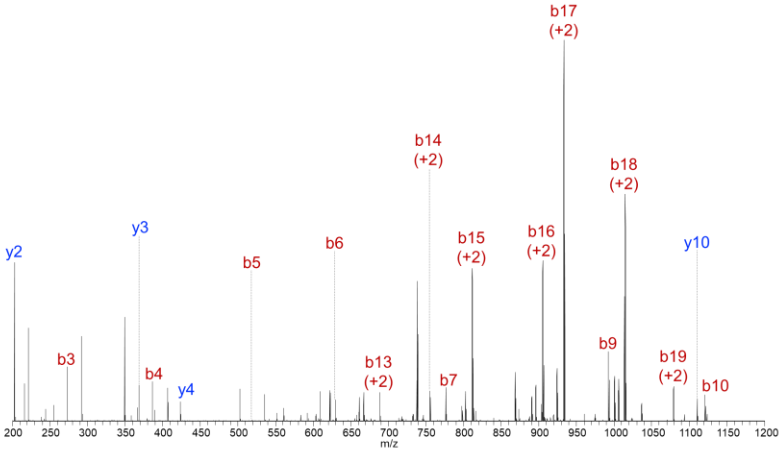

Figure S27. A) UHPLC-HRMS EIC traces showing the peaks of linear substrate and product. Red star on trace (*) indicates peak being analyzed. B) MS2 fragmentation of the linear ArbA2-FLWGY-NH2$_{58\text{-}77}$ substrate.

Note: These assays were completed with C-terminal amidated peptides instead of a carboxy terminus. This had no significant impact on conversion levels of the native FLWGY core. The lower ionization efficiency of the y-ions necessitated use of the top 300 MS2 fragments.

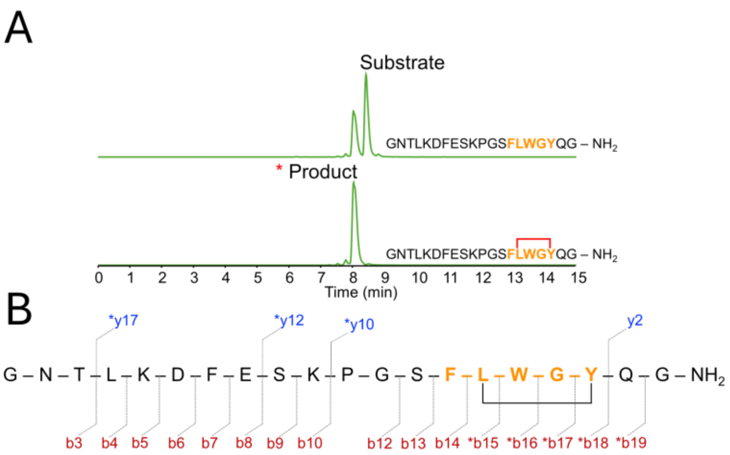

Fragment (z)	m (obs)	m (cal)	Δm (ppm)
b2(+1)	-	172.0717	-
b3(+1)	273.1193	273.1188	2.01
b4(+1)	386.2034	386.2035	0.23
b5(+1)	514.2984	514.2991	1.42
b6(+1)	629.3253	629.3271	2.84
b7(+1)	776.3937	776.394	0.36
b8(+1)	905.4363	905.4372	0.98
b9(+1)	992.4683	992.4704	2.07
b10(+1)	1120.5633	1120.5619	1.25
b11(+2)	-	609.3117	-
b12(+2)	637.8224	637.8244	3.13
b13(+2)	681.3384	681.3416	4.67
b14(+2)	754.8726	754.8737	1.43
b15(+2)*	810.4068	810.4079	1.33
b16(+2)*	903.4465	903.4469	0.47
b17(+2)*	931.9572	931.9592	2.14
b18(+2)*	1013.4889	1013.4901	1.21
b19(+2)*	1077.5182	1077.5185	0.31

Fragment (z)	m (obs)	m (cal)	Δm (ppm)
y2(+1)	203.1139	203.1135	1.82
y3(+1)	-	366.1772	-
y4(+1)	-	423.1987	-
y5(+1)	-	609.2780	-
*y6(+1)	-	720.3464	-
*y7(+1)	-	867.4148	-
*y8(+1)	-	954.4469	-
*y9(+1)	-	1011.4683	-
*y10(+1)	1108.5210	1108.5206	0.39
*y11(+2)	-	618.8117	-
*y12(+2)	662.3276	662.3281	0.68
*y13(+2)	-	726.849	-
*y14(+2)	-	800.3832	-
*y15(+2)	-	857.8967	-
*y16(+2)	-	921.9441	-
*y17(+3)	652.6598	652.6607	1.31
*y18(+2)	-	1029.0100	-
*y19(+2)	-	1086.0315	-

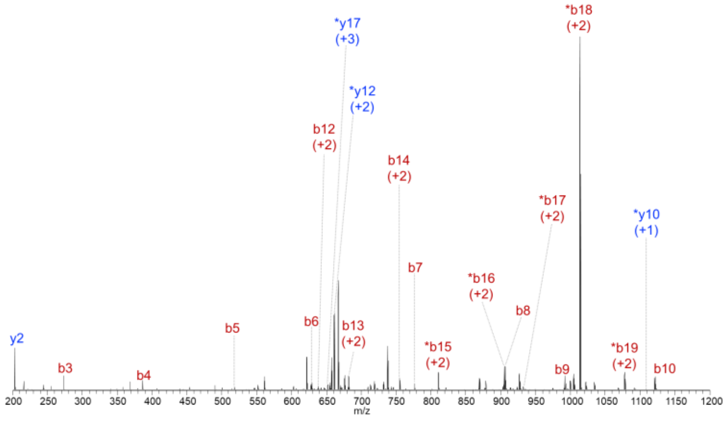

Figure S28. A) UHPLC-HRMS EIC traces showing the peaks of linear substrate and product. Red star on trace (*) indicates peak being analyzed. B) MS2 fragmentation of the linear ArbA2-FLWGY-NH2$_{58\text{-}77}$ product. Stars (*) indicate the loss of two hydrogens in the MS2 fragment ions. Core peptide is highlighted in orange.

Note: These assays were completed with C-terminal amidated peptides instead of a carboxy terminus. This had no significant impact on conversion levels of the native F-L-W-G-Y core. The lower ionization efficiency of the y-ions necessitated use of the top 300 MS2 fragments.

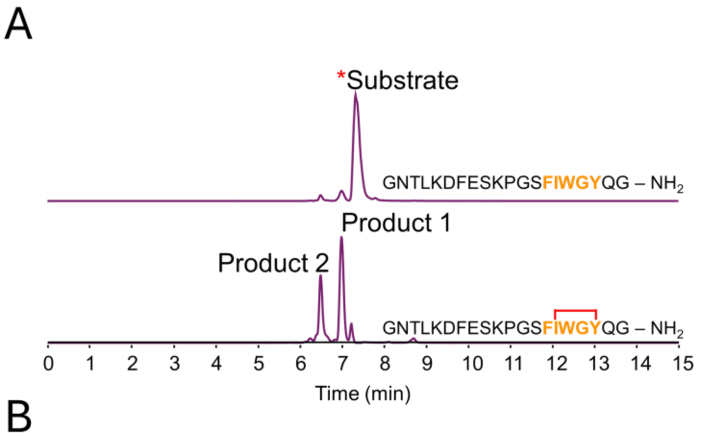

B

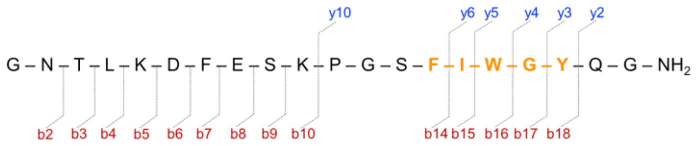

Fragment (z)	m (cal)	m (obs)	Δm (ppm)
b2(+1)	172.0717	172.0714	1.57
b3(+1)	273.1194	273.1189	1.65
b4(+1)	386.2034	386.2034	0.03
b5(+1)	514.2984	514.3	3.17
b6(+1)	629.3253	629.3274	3.32
b7(+1)	776.3937	776.3944	0.88
b8(+1)	905.4363	905.4359	0.45
b9(+1)	992.4683	992.47	1.67
b10(+1)	1120.563	1120.56	2.59
b11(+2)	-	609.312	-
b12(+2)	-	637.822	-
b13(+2)	-	681.338	-
b14(+2)	754.8726	754.8737	1.43
b15(+2)	811.4147	811.4159	1.54
b16(+2)	904.4543	904.4573	3.31
b17(+2)	932.965	932.9654	0.39
b18(+2)	1014.497	1014.499	2.07
b19(+2)	-	1078.526	-

Fragment (z)	m (cal)	m (obs)	Δm (ppm)
y2(+1)	203.1139	203.1136	1.33
y3(+1)	366.1772	366.178	2.18
y4(+1)	423.1987	423.1994	1.75
y5(+1)	609.278	609.279	1.69
y6(+1)	-	722.362	-
y7(+1)	-	869.43	-
y8(+1)	-	956.463	-
y9(+1)	-	1013.48	-
y10(+1)	1110.537	1110.538	0.91
y11(+2)	-	619.82	-
y12(+2)	-	663.336	-
y13(+2)	-	727.857	-
y14(+2)	-	801.391	-
y15(+2)	-	858.905	-
y16(+2)	-	922.952	-
y17(+2)	-	979.494	-
y18(+2)	-	1030.02	-
y19(+2)	-	1087.04	-

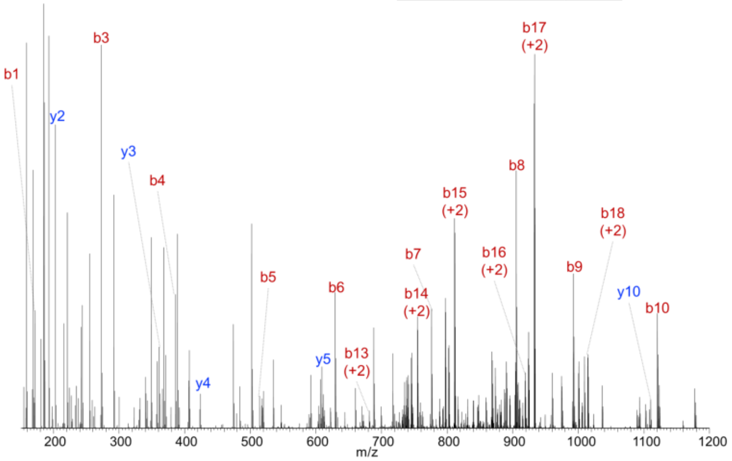

Figure S29. A) UHPLC-HRMS EIC traces showing the peaks of linear substrate and two product peaks. Red star on trace (*) indicates peak being analyzed. B) MS2 fragmentation of the linear ArbA2-FIWGY-NH2$_{58\text{-}77}$ product. Stars (*) indicate the loss of two hydrogens in the MS2 fragment ions. Core peptide is highlighted in orange.

Note: These assays were completed with C-terminal amidated peptides instead of a carboxy terminus. This had no significant impact on conversion levels of the native F-L-W-G-Y core. The lower ionization efficiency of the y-ions necessitated use of the top 300 MS2 fragments.

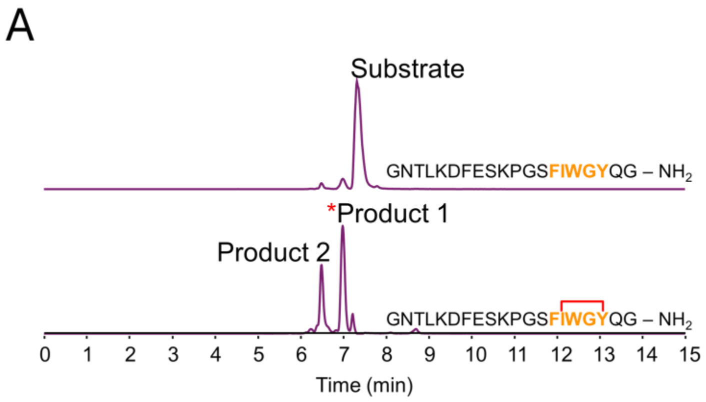

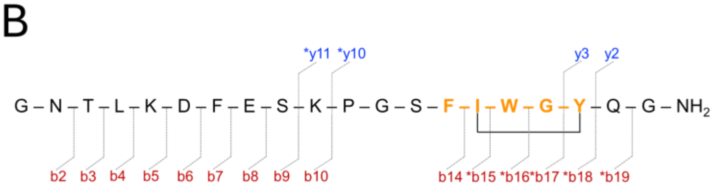

Fragment (z)	m (cal)	m (obs)	Δm (ppm)
b2(+1)	172.0717	172.0715	0.99
b3(+1)	273.1194	273.119	1.28
b4(+1)	386.2034	386.2031	0.80
b5(+1)	514.2984	514.3004	3.95
b6(+1)	629.3253	629.3276	3.64
b7(+1)	776.3937	776.3944	0.88
b8(+1)	905.4363	905.4369	0.65
b9(+1)	992.4683	992.4699	1.57
b10(+1)	1120.563	1120.56	3.12
b11(+2)	-	609.312	-
b12(+2)	-	637.822	-
b13(+2)	-	681.338	-
b14(+2)	754.8727	754.8726	0.11
*b15(+2)	810.4071	810.4068	0.35
*b16(+2)	903.4458	903.4465	0.75
*b17(+2)	931.9574	931.9572	0.21
*b18(+2)	1013.489	1013.489	0.52
*b19(+2)	-	1077.518	-

Fragment (z)	m (cal)	m (obs)	Δm (ppm)
y2(+1)	203.1139	203.1136	1.33
y3(+1)	366.1772	366.1775	0.82
y4(+1)	-	421.1831	-
y5(+1)	-	607.2624	-
*y6(+1)	-	720.3464	-
*y7(+1)	-	867.4148	-
*y8(+1)	-	954.4469	-
*y9(+1)	-	1011.4683	-
*y10(+1)	1108.521	1108.522	0.78
*y11(+1)	1236.616	1236.614	1.61
*y12(+2)	-	662.3277	-
*y13(+2)	-	726.8490	-
*y14(+2)	-	800.3832	-
*y15(+2)	-	857.8967	-
*y16(+2)	-	921.9441	-
*y17(+2)	-	978.4862	-
*y18(+2)	-	1029.01	-
*y19(+2)	-	1086.032	-

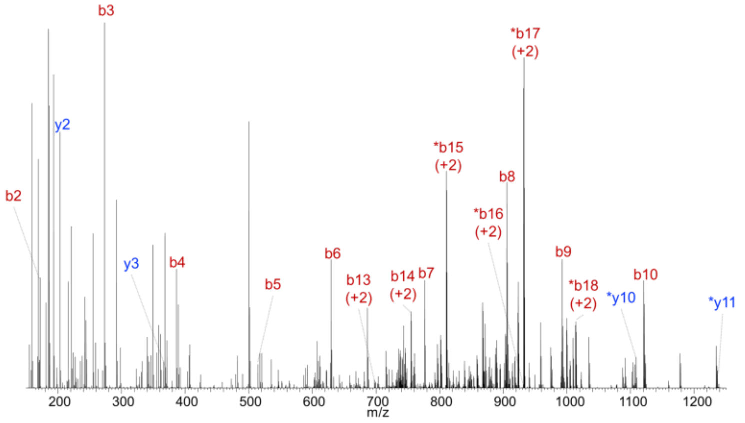

Figure S30. A) UHPLC-HRMS EIC traces showing the peaks of linear substrate and two product peaks. Red star on trace (*) indicates peak being analyzed. B) MS2 fragmentation of product 1 of ArbA2-FIWGY-NH2$_{58\text{-}77}$. Stars (*) indicate the loss of two hydrogens in the MS2 fragment ions. Core peptide is highlighted in orange.

Note: These assays were completed with C-terminal amidated peptides instead of a carboxy terminus. This had no significant impact on conversion levels of the native F-L-W-G-Y core. The lower ionization efficiency of the y-ions necessitated use of the top 300 MS2 fragments.

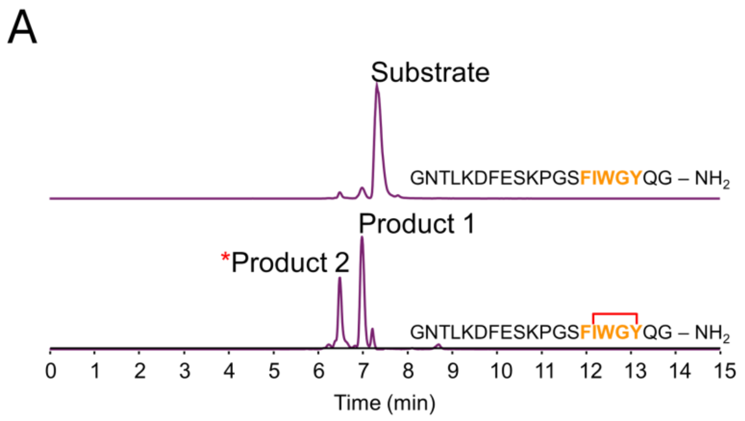

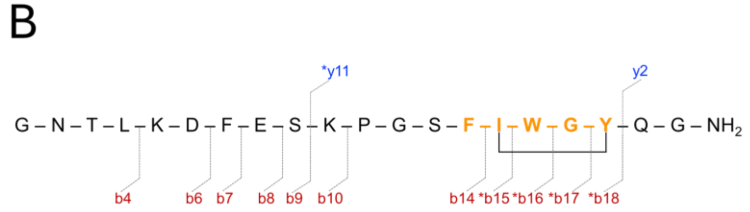

Fragment (z)	m (cal)	m (obs)	Δm (ppm)
b2(+1)	172.0717	172.0714	1.57
b3(+1)	273.1194	273.1189	1.65
b4(+1)	386.2034	386.2034	0.03
b5(+1)	514.2984	514.3	3.17
b6(+1)	629.3253	629.3274	3.32
b7(+1)	776.3937	776.3944	0.88
b8(+1)	905.4363	905.4359	0.45
b9(+1)	992.4683	992.47	1.67
b10(+1)	1120.563	1120.56	2.59
b11(+1)	-	609.312	-
b12(+2)	-	637.822	-
b13(+2)	681.3384	681.3415	4.53
b14(+2)	754.8726	754.8733	0.90
b15(+2)	810.4068	810.4064	0.52
b16(+2)	903.4465	903.4484	2.13
b17(+2)	931.9572	931.9553	2.04
b18(+2)	1013.489	1013.489	0.23
b19(+2)	-	1077.519	-

Fragment (z)	m (cal)	m (obs)	Δm (ppm)
y2(+1)	203.1139	203.1135	1.82
y3(+1)	-	366.1775	-
y4(+1)	-	421.1831	-
y5(+1)	-	607.2624	-
y6(+1)	-	720.3464	-
y7(+1)	-	867.4148	-
y8(+1)	-	954.4469	-
y9(+1)	-	1011.4683	-
y10(+1)	-	1108.521	-
y11(+2)	1236.616	1236.614	1.69
y12(+2)	-	662.3277	-
y13(+1)	-	726.8490	-
y14(+1)	-	800.3832	-
y15(+1)	-	857.8967	-
y16(+2)	-	921.9441	-
y17(+2)	-	978.4862	-
y18(+2)	-	1029.01	-
y19(+1)	-	1086.032	-

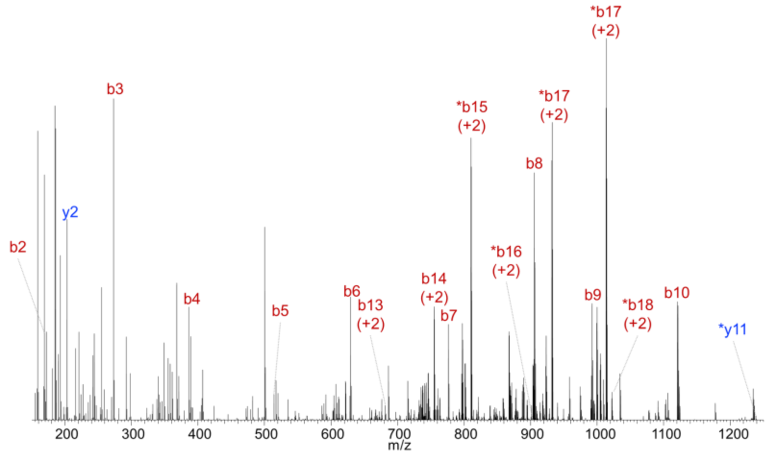

Figure S31. A) UHPLC-HRMS EIC traces showing the peaks of linear substrate and two product peaks. Star (*) indicates peak being analyzed. B) MS2 fragmentation of product 2 of ArbA2-FIWGY-NH2$_{58\text{-}77}$. Stars (*) indicate the loss of two hydrogens in the MS2 fragment ions. Core peptide is highlighted in orange.

Note: These assays were completed with C-terminal amidated peptides instead of a carboxy terminus. This had no significant impact on conversion levels of the native F-L-W-G-Y core. The lower ionization efficiency of the y-ions necessitated use of the top 300 MS2 fragments.

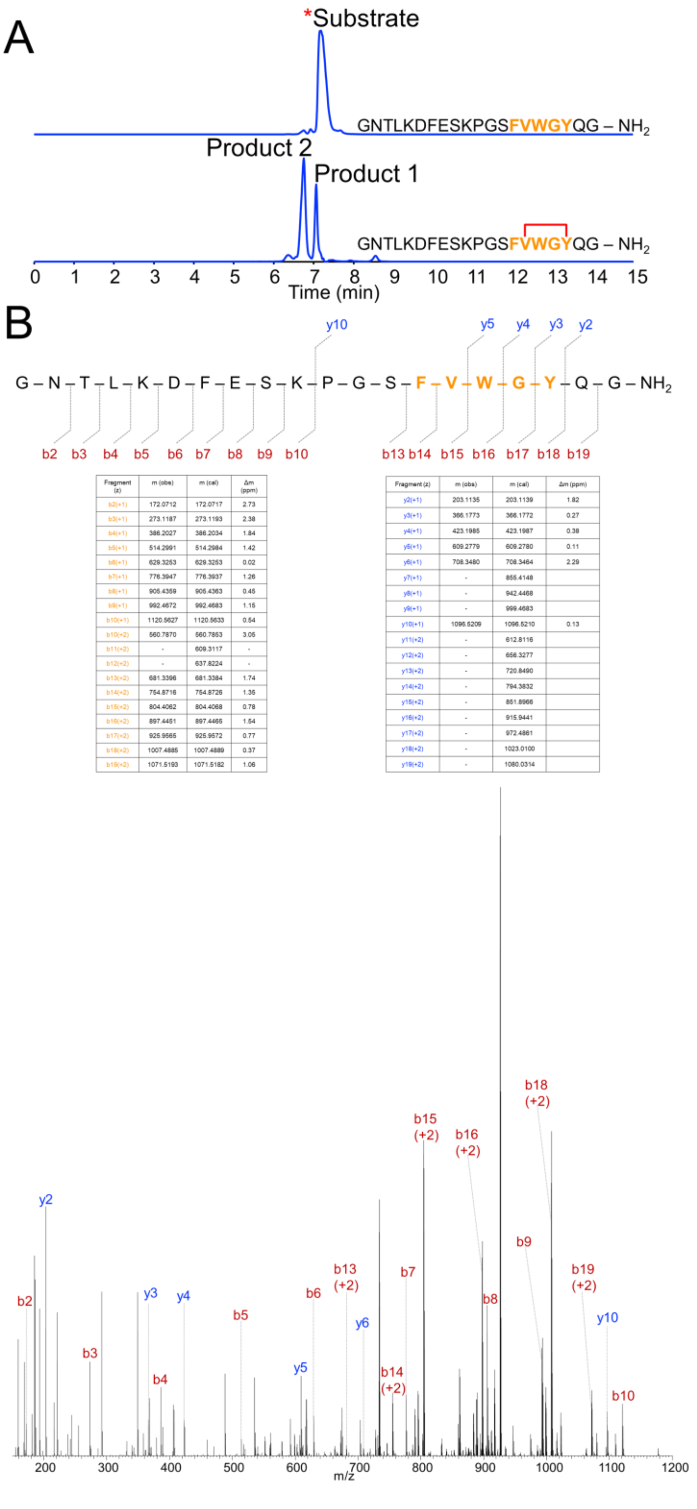

Fragment (z)	m (obs)	m (cal)	Δm (ppm)
b2(+1)	172.0712	172.0717	2.73
b3(+1)	273.1187	273.1193	2.38
b4(+1)	386.2027	386.2034	1.84
b5(+1)	514.2991	514.2984	1.42
b6(+1)	629.3253	629.3253	0.02
b7(+1)	776.3947	776.3937	1.26
b8(+1)	905.4359	905.4363	0.45
b9(+1)	992.4672	992.4683	1.15
b10(+1)	1120.5627	1120.5633	0.54
b10(+2)	560.7870	560.7853	3.05
b11(+2)	-	609.3117	-
b12(+2)	-	637.8224	-
b13(+2)	681.3396	681.3384	1.74
b14(+2)	754.8716	754.8726	1.36
b15(+2)	804.4062	804.4068	0.78
b16(+2)	897.4451	897.4465	1.54
b17(+2)	925.9565	925.9572	0.77
b18(+2)	1007.4885	1007.4889	0.37
b19(+2)	1071.5193	1071.5182	1.06

Fragment (z)	m (obs)	m (cal)	Δm (ppm)
y2(+1)	203.1135	203.1139	1.82
y3(+1)	366.1773	366.1772	0.27
y4(+1)	423.1985	423.1987	0.38
y5(+1)	609.2779	609.2780	0.11
y6(+1)	708.3480	708.3464	2.29
y7(+1)	-	855.4148	
y8(+1)	-	942.4468	
y9(+1)	-	999.4683	
y10(+1)	1096.5209	1096.5210	0.13
y11(+2)	-	612.8116	
y12(+2)	-	656.3277	
y13(+2)	-	720.8490	
y14(+2)	-	794.3832	
y15(+2)	-	851.8966	
y16(+2)	-	915.9441	
y17(+2)	-	972.4861	
y18(+2)	-	1023.0100	
y19(+2)	-	1080.0314	

Figure S32. A) UHPLC-HRMS EIC traces showing the peaks of linear substrate and two product peaks. Red star on trace (*) indicates peak being analyzed. B) MS2 fragmentation of linear ArbA2-FVWGY-NH2$_{58\text{-}77}$. Stars (*) indicate the loss of two hydrogens in the MS2 fragment ions. Core peptide is highlighted in orange

Note: These assays were completed with C-terminal amidated peptides instead of a carboxy terminus. This had no significant impact on conversion levels of the native F-L-W-G-Y core. The lower ionization efficiency of the y-ions necessitated use of the top 300 MS2 fragments.

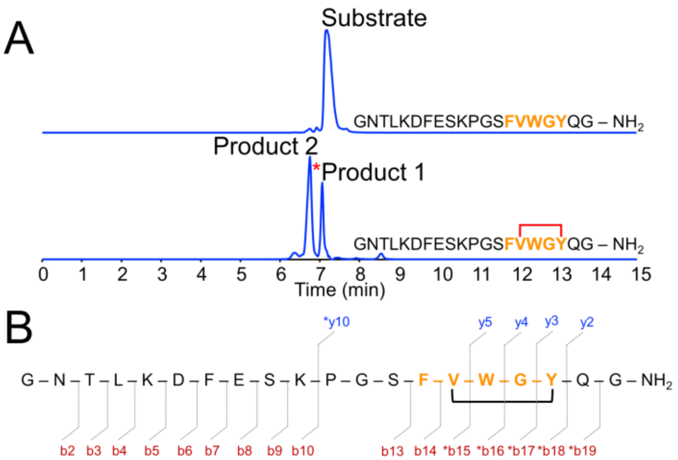

Fragment (z)	m (obs)	m (cal)	Δm (ppm)
b2(+1)	172.0712	172.0717	2.73
b3(+1)	273.1189	273.1193	1.65
b4(+1)	386.2030	386.2034	1.06
b5(+1)	514.2989	514.2984	1.03
b6(+1)	629.3253	629.3253	0.02
b7(+1)	776.3937	776.3937	0.03
b8(+1)	905.4359	905.4363	0.46
b9(+1)	992.4730	992.4683	4.70
b10(+1)	1120.5618	1120.5633	1.34
b11(+2)	-	609.3117	-
b12(+2)	-	637.8224	-
b13(+2)	681.3377	681.3384	1.05
b14(+2)	754.8716	754.8726	1.35
b15(+2)*	803.3993	803.3990	0.35
b16(+2)*	896.4388	896.4387	0.14
b17(+2)*	924.9490	924.9494	0.44
b18(+2)*	1006.4811	1006.4811	0.03
b19(+2)*	1070.5118	1070.5104	1.35

Fragment (z)	m (obs)	m (cal)	Δm (ppm)
y2(+1)	203.1135	203.1139	1.82
y3(+1)	366.1773	366.1772	0.27
y4(+1)	423.1993	423.1987	1.51
y5(+1)	609.2780	609.2780	0.71
*y6(+1)	706.3316	706.3308	1.60
*y7(+1)	-	853.3992	
*y8(+1)	-	940.4312	-
*y9(+1)	-	997.4527	-
*y10(+1)	1094.5024	1094.5054	2.77
*y11(+2)	-	611.8038	-
*y12(+2)	-	655.3199	-
*y13(+2)	-	719.8411	-
*y14(+2)	-	793.3754	-
*y15(+2)	-	850.8888	-
*y16(+2)	-	914.9363	-
*y17(+2)	-	971.4783	-
*y18(+2)	-	1022.0022	-
*y19(+2)	-	1079.0236	-

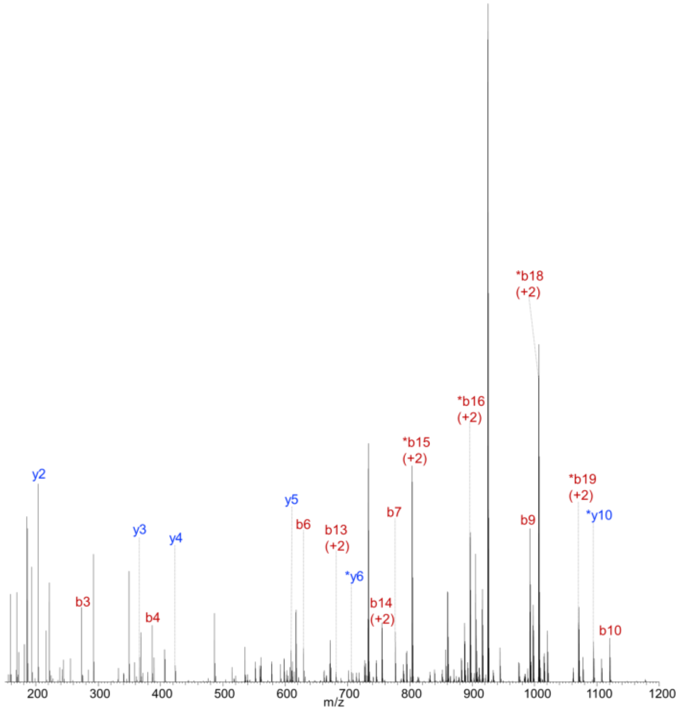

Figure S33. A) UHPLC-HRMS EIC traces showing the peaks of linear substrate and two product peaks. Red star on trace (*) indicates peak being analyzed. B) MS2 fragmentation of product 1 of ArbA2-FVWGY-NH2$_{58\text{-}77}$. Stars (*) indicate the loss of two hydrogens in the MS2 fragment ions. Core peptide is highlighted in orange.

Note: These assays were completed with C-terminal amidated peptides instead of a carboxy terminus. This had no significant impact on conversion levels of the native F-L-W-G-Y core. The lower ionization efficiency of the y-ions necessitated use of the top 300 MS2 fragments.

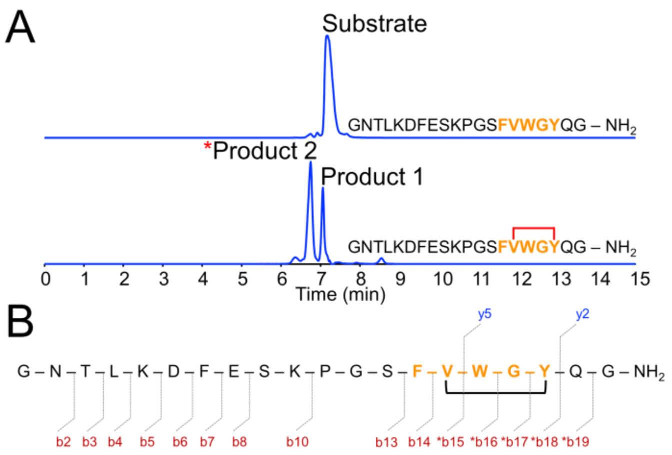

Fragment (z)	m (obs)	m (cal)	Δm (ppm)
b2(+1)	172.0712	172.0717	2.73
b3(+1)	273.1189	273.1193	1.65
b4(+1)	386.2029	386.2034	1.32
b5(+1)	514.2999	514.2984	2.97
b6(+1)	629.3255	629.3253	0.30
b7(+1)	776.3943	776.3937	0.75
b8(+1)	905.4350	905.4363	1.45
b9(+1)	-	992.4683	-
b10(+1)	1120.5633	1120.5633	0.00
b11(+2)	-	609.3117	-
b12(+2)	-	637.8224	-
b13(+2)	681.3379	681.3384	0.76
b14(+2)	754.8719	754.8726	0.95
b15(+2)*	803.3993	803.3990	0.35
b16(+2)*	896.4377	896.4387	1.09
b17(+2)*	924.9498	924.9494	0.43
b18(+2)*	1006.4823	1006.4811	1.22
b19(+2)*	1070.5113	1070.5104	0.88
b19(+3)*	714.0094	714.0093	0.09

Fragment (z)	m (obs)	m (cal)	Δm (ppm)
y2(+1)	203.1136	203.1139	1.33
y3(+1)	-	366.1772	-
y4(+1)	-	423.1987	-
y5(+1)	609.2809	609.2780	4.81
*y6(+1)	-	706.3308	-
*y7(+1)	-	853.3992	-
*y8(+1)	-	940.4312	-
*y9(+1)	-	997.4527	-
*y10(+1)	-	1094.5054	-
*y11(+2)	-	611.8038	-
*y12(+2)	-	655.3199	-
*y13(+2)	-	719.8411	-
*y14(+2)	-	793.3754	-
*y15(+2)	-	850.8888	-
*y16(+2)	-	914.9363	-
*y17(+2)	-	971.4783	-
*y18(+2)	-	1022.0022	-
*y19(+2)	-	1079.0236	-

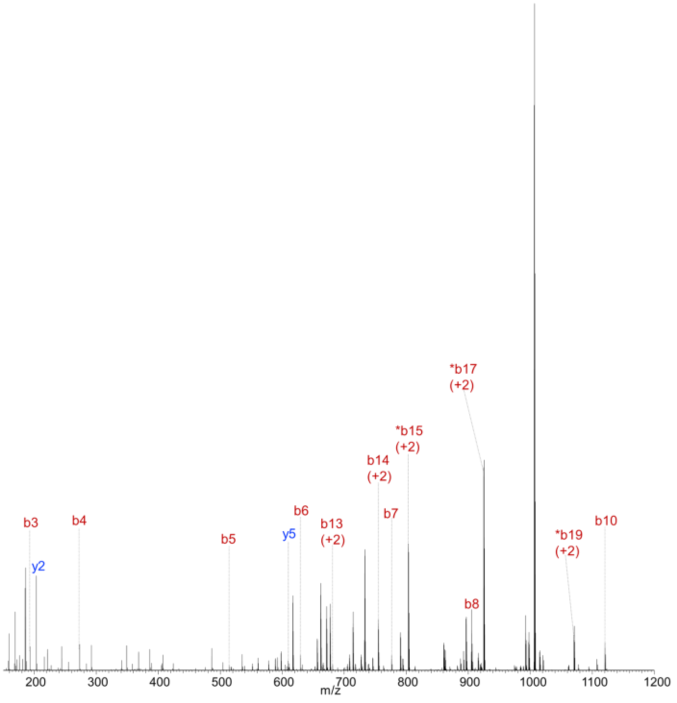

Figure S34. A) UHPLC-HRMS EIC traces showing the peaks of linear substrate and two product peaks. Red star on trace (*) indicates peak being analyzed. B) MS2 fragmentation of product 2 of ArbA2-FVWGY-$NH2_{58\text{-}77}$. Stars (*) indicate the loss of two hydrogens in the MS2 fragment ions. Core peptide is highlighted in orange.

Note: These assays were completed with C-terminal amidated peptides instead of a carboxy terminus. This had no significant impact on conversion levels of the native F-L-W-G-Y core. The lower ionization efficiency of the y-ions necessitated use of the top 300 MS2 fragments.

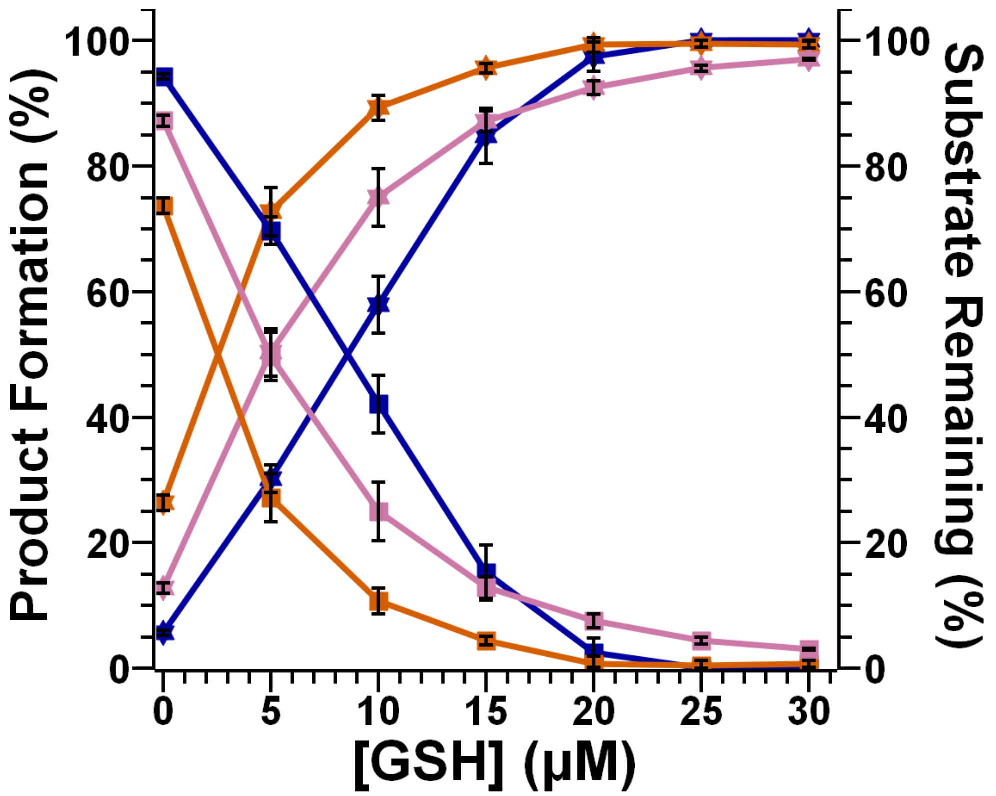

Predicted Fragments	Number of Missed Cleavages	*m/z* for $[M+H]^+$	0 µM GSH	5 µM GSH	10 µM GSH	15 µM GSH	20 µM GSH
PGSFLWGYQGNDAESK*	0	585.9373	1.31E+04	1.06E+04	5.40E+03	4.09E+03	ND
PGSFLWGYQGNDVESK	0	595.6154	7.85E+03	5.88E+03	1.67E+03	7.52E+02	ND
GFESKPGSFLWGYQGNDAESKSK	2	840.7337	ND	ND	ND	ND	ND
GFESKPGSFLWGYQGNDVESKSK	2	850.0775	ND	ND	ND	ND	ND
DFESKPGSFLWGYQGNDAESKSK	2	860.0689	ND	ND	ND	ND	ND
EEKPLMKGFESKPGSFLWGYQGNDAESK	3	1054.1756	ND	ND	ND	ND	ND
TSNGNTLKDFESKPGSFLWGYQGNDAESK	3	1060.1645	4.95E+03	4.08E+03	7.03E+02	3.44E+02	ND
EEKPLMKGFESKPGSFLWGYQGNDVESK	3	1063.5193	ND	ND	ND	ND	ND
Analyzed Fragments							
GFESKPGSFLWGYQGNDAESK	1	769.0248	6.43E+05	2.85E+05	1.37E+05	3.90E+04	2.14E+04
GFESKPGSFLWGYQGNDVESK	1	778.3685	5.33E+05	3.32E+05	1.33E+05	6.23E+04	4.48E+04
DFESKPGSFLWGYQGNDAESK	1	788.3599	6.36E+05	5.27E+05	3.79E+05	1.07E+05	2.80E+04

ND = not detected. Peptides were analyzed by LC-MS. 'Not detected' indicates signal below detection threshold.

Figure S35. (Top) Depletion of unmodified core peptides over the course of the ArbB2 reaction, demonstrating stepwise processing. Stars represent product formation and squares are remaining substrate. Cores are colored as followed: Core 2 (vermillion), Core 3 (salmon), and Core 1 (Blue). (Bottom) Quantification of unmodified trypsinized peptides with differing numbers of missed cleavages. For analysis, the possible trypsin fragments were extracted in the unmodified peptide to determine the ideal core fragments to monitor. Minimal amounts of proteolyzed cores were detected for two and three missed trypsin cleavages while the zero missed cleavage fragments have two cores with identical masses (indicated by star *). Therefore, analysis for product formation of each core was then conducted with singly missed cleavage substrate and products. Intensities are reported as normalization levels (NL). ND is not detected.

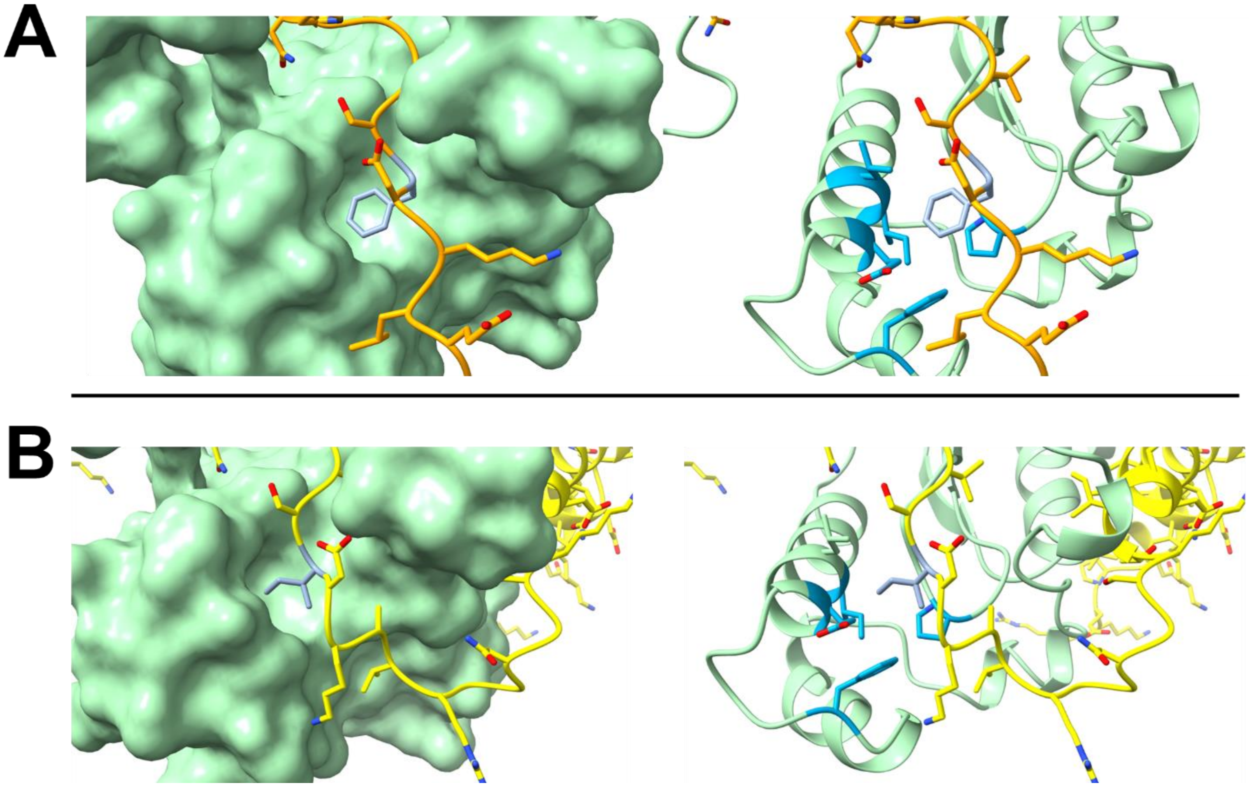

Figure S36. A) Surface model (Left) of CamB1(light mint green) with CamA1-ILLY$_{113-132}$ (orange) showing the conserved Phe (steel blue) in the binding pocket, Binding pocket residues within 5 Å of the conserved Phe are shown (Right). B) Surface model (Left) of CamB1(light mint green) with CamA2-ILWY$_{56-75}$ (yellow) showing Ile (steel blue) fitting within the same binding pocket. Binding pocket residues within 5 Å of the Ile variant are shown (Right).

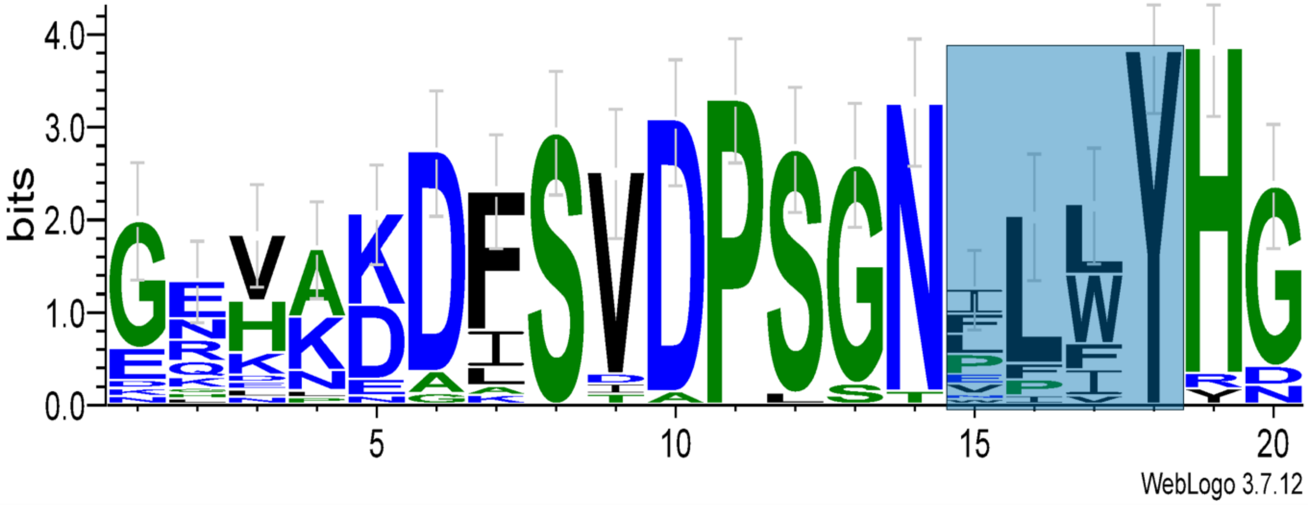

>CamA1 (See main text reference 24)
GNFFFYHNNKNAVAKSKADEGELKDFSVDPSGN**FFFY**HNNKN
AVAKSKADEGELKDFSVDPSGN**FFFY**HNNKNAVAKSKADEGEL
KDFSVDPSGN**FFFY**HNNKNAVAKSKADEGELKDFSVDPSGN**IL**
LYHGNKNAEVKSNTDGKHKEDFSVDPSGNPLLYHDNKNDDFS
VDPSGNPLLYHDNKNGEHKDGFSVDPSGNPLFYHGNKNDDF
SVDPSGNPLFYHGNKNGEHKDGFSVDPSGNPLLYHDNKNDD
FSVDPSGNPLFYHGNKNGEHKDGFSVDPSGNPLFYHGNKNG
EHKDGFSVDPSG

>CamA2 (See main text reference 24)
MKTFFALFAFSSLLLLSSTITARKEPEYLKTVIENQPILEVLQGVL
DLITKSGNGGRVAKDISVDPSGN**ILWY**HGNKNAEAKSKDDGEH
KDDFSVDPSGNELWYHDNKNAEAKSKADGEHKDDFSVDPSG
NELWYHDNKNAEAKSINPSGNVLWYHGNKNAEAKSINPSGNV
LWYHGNKNAEAKSKADGEHKDDFSVDPSGNVLWYHGKKIVEL
DSETIAEQVAKAFSDAPSGTFILYHGNKNGESNSKASGEGAAK
DSFVDPSVFDK*

>Ceanothus_americanus_27108 (See main text reference 24)
MKTFFVLLAFSSLLLSRTITARKEPAEYAKTIMENQPIPVLEVIQG
VLDLIAKSSRNGGRLAKDLSVDPLSNWLIYRGNQNGENVAKDLK
DLSVDPSSNFLIYRGNHNGENAAKDLSVDPLSNWLIYRGNQNG
ENVAKDLKDLSVDPSSNFLIYRGNHNEENVAKDLSVDPSSNFLI
YRGNHNEENVAKDLSVDPSSNFLIYRGNHNEENVAKDLSVDPS
SNFLIYHGNKNADPNSKANGEGVAKGISPDHHDQ*

>Ceanothus_americanus_27098 (See main text reference 24)
PKKHFEFFEVDIKMKTFFALFAFSSLLLLSSTITARKEPEYLKTVI
ENQPILEVLQGVLDLITKSGNGGRVAKDISVDPSGNILWYHGNK
NAEAKSKDDGEHKDDFSVDPSGNVLWYHGNKNAEAKSKADG
EHKDDFSVDPSGNVLWYHGKKIVELDSETIAEQVAKAFSDAPS
GTFILYHGNKNGESNSKASGEGAAKDSFVDPSVFDK*

>Ceanothus_americanus_18149 (See main text reference 37)
LLWYHGNKNSEAKSKADGEHKNDFSVDPSGNILWYHGKKIVEL
DSETIAEQVAKAFSDAPSGTFILYHGNKNGESNSKASGEGAAK
DSFVDPSVFD

>Ceanothus_americanus_1604 (See main text reference 37)
MKTFFALFAFSSLLLLSSTITARKEPEYVRTIIENQPILEVLQGVL
DLITKSGNGGRVAKDISVDPSGNILWYHGNKNAEAKSKDDGEH
KDDFSVDPSGNVLWYHGNKNAEAKSKADGEHKDDFSVDPSG
NELWYHGNKNAEAKSIDPSGNVLWYHGNKNAXXXXXXXXXXX
XXXXXXXXXXXXXXXXXXXXXXXADGEHKDDFSVDPSGNVLW
YHGNKNAEAKSKADGEHKDDFSVDPSG

Ceanothus_americanus_7929 (See main text reference 37)
MKTFFALFAFSSLLLLSSTITARKEPEYVRTIIENQPILEVLQGVL
DLITKSGNGGRVAKDISVDPSGNLPLYHGNKNGEHVPKDASVD
PSDGNQNGEQVAKDFSVDPSGNNLFYYNNKNAVAKSKADGG
ELKDFSVDPSGNILLYHGNKNAEVKSNTDGKHKEDFSVDPSG
NPLLYHDNKNDDFSVDPSGNPLLYHDNKNGEHKDGFSVDPSG
NPLFYHGNKNDDFSVDPSGNPLFYHGNKNGEHKD

Ceanothus_americanus_16032 (See main text reference 37)
MKTLFALFVFSSLLLLSSTITARKEPEYVKTVIENQPILEVLQGVL
DLITKSRNGGRVAKDISVDPSGNILVYHGNKNGEHVPKDASVD
PSGNILLYHGNQNGKQVAKDFSVDPSGNFFIYHNNKNAVAKSK
ADGGELKDFSVDPSGNILLYHGNKNAEVKSNTDGKHKEDFSV
DPSGNPLLYHDNKNDDFSVDPSGNPLLYHDNKME

Figure S37. Weblogo containing the sequences of all unique precursor peptides found within the transcriptome of *C. americanus*. The cores are color coded to make the amino acid at the (-8) position. Those highlighted and underlined in red correspond to peptides used in the reactions, cores in red contain the conserved Phe, those highlighted in blue contain an Ile, those highlighted in green contain Lys, and those highlighted in orange contain Leu. The sequences highlighted in Purple contain Ala.

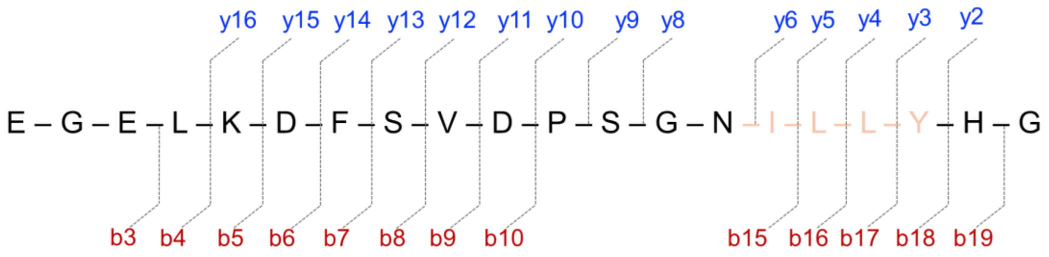

Fragment (z)	m (obs)	m (cal)	Δm (ppm)
b1(+1)	-	130.0499	-
b2(+1)	-	187.0713	-
b3(+1)	316.1141	316.1139	0.57
b4(+1)	429.1988	429.1980	1.9
b5(+1)	557.2942	557.2929	2.26
b6(+1)	672.3217	672.3199	2.71
b7(+1)	819.3899	819.3883	1.96
b8(+1)	906.4199	906.4203	0.46
b9(+1)	1005.4896	1005.4887	0.87
b10(+1)	1120.5152	1120.5157	0.42
b11(+1)	-	1217.5685	-
b12(+1)	-	1304.6005	-
b13(+1)	-	1361.622	-
b14(+1)	-	1475.6649	-
b15(+1)	1588.7492	1588.7489	0.18
b16(+1)	1701.8355	1701.8330	1.49
B17(+1)	1814.9155	1814.9170	0.84
b18(+1)	1977.9918	1977.9804	5.78
b19(+2)	1058.0239	1058.0233	0.567095
b20	-	---	-

Fragment (z)	m (obs)	m (cal)	Δm (ppm)
y1(+1)	-	76.0393	-
y2(+1)	213.0982	213.0982	0.23
y3(+1)	376.1621	376.1616	1.38
y4(+1)	489.2463	489.2456	1.35
y5(+1)	602.3314	602.3297	2.82
y6(+1)	715.4181	715.4138	6.07
y7(+1)	-	829.4567	-
y8(+1)	886.4786	886.4782	0.51
y9(+1)	973.5042	973.5102	6.14
y10(+1)	1070.5637	1070.5629	0.71
y11(+1)	1185.5885	1185.5899	1.16
y12(+1)	1284.6581	1284.6581	0.15
y13(+1)	1371.6908	1371.6903	0.35
y14(+1)	1518.7559	1518.7587	1.86
y15(+1)	1633.7857	1633.7857	0.02
y16(+1)	1761.8767	1761.8806	2.23
Y17(+2)	-	937.9860	-
Y18(+1)	-	2004.0070	-
Y19(+1)	-	2061.0290	-
y20	-	-	-

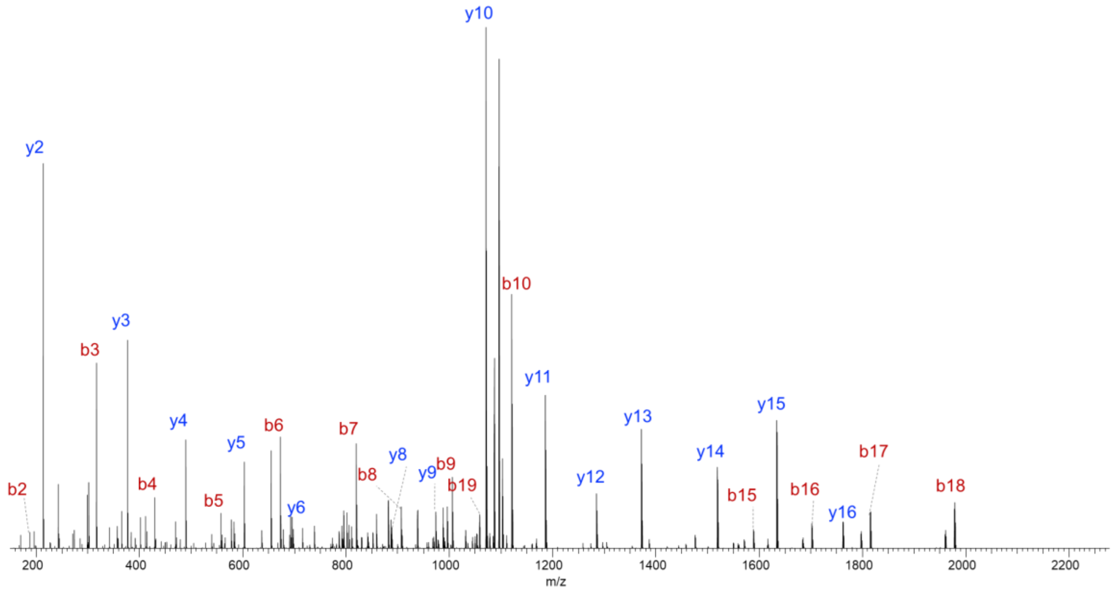

Figure S38. MS2 fragmentation of the linear CamA1-ILLY$_{113\text{-}132}$ substrate. Core peptide is highlighted in salmon.

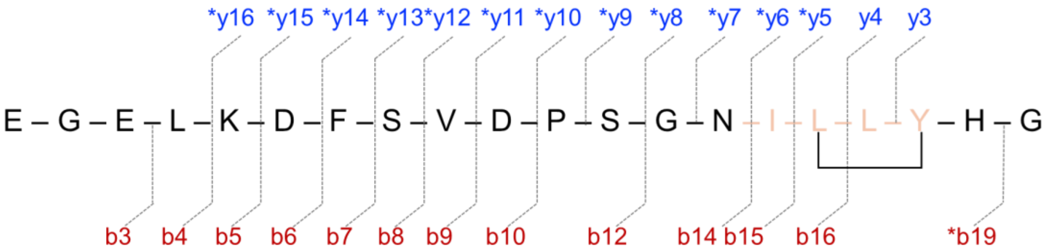

Fragment (z)	m (obs)	m (cal)	Δm (ppm)
b1(+1)	-	130.0499	-
b2(+1)	-	187.0713	-
b3(+1)	316.1139	316.1139	0.06
b4(+1)	429.1986	429.1980	1.44
b5(+1)	557.2938	557.2929	1.54
b6(+1)	672.3211	672.3199	1.96
b7(+1)	819.3899	819.3883	1.96
b8(+1)	906.4205	906.4203	0.20
b9(+1)	1005.4893	1005.4887	0.57
b10(+1)	1120.5147	1120.5157	0.87
b11(+1)	-	1217.5684	-
b12(+1)	-	1304.6005	-
b13(+1)	-	1361.6219	-
b14(+1)	1475.6656	1475.6649	0.51
b15(+1)	1588.7518	1588.7489	1.82
*b16(+1)	1699.8171	1699.8173	0.11
*b17(+1)	-	1814.9171	-
*b18(+1)	-	1975.9648	-
*b19(+2)	1057.0167	1057.0155	1.14
*b20	-	-	-

Fragment (z)	m (obs)	m (cal)	Δm (ppm)
y1(+1)	-	76.0393	-
y2(+1)	213.0980	213.0982	1.17
Y3(+I)	376.1619	376.1616	0.85
y4(+1)	489.2457	489.2456	0.12
*y5(+1)	600.3156	600.3140	2.63
*y6(+1)	713.3990	713.3981	1.29
*y7(+1)	827.4413	827.4410	0.35
*y8(+1)	884.4618	884.4625	0.76
*y9(+1)	971.4924	971.4945	2.16
*y10(+1)	1068.5457	1068.5473	1.46
*y11(+1)	1183.5722	1183.5742	1.69
*y12(+1)	1282.6406	1282.6426	1.57
*y13(+1)	1369.6731	1369.6746	1.12
*y14(+1)	1516.7404	1516.7431	1.75
*y15(+1)	1631.7729	1631.7700	1.78
*y16(+1)	1759.8621	1759.8650	1.62
*y17(+1)	-	1872.9491	-
*y18(+1)	-	2001.9917	-
*y19(+1)	-	2059.0131	-
*y20(+1)	-	-	-

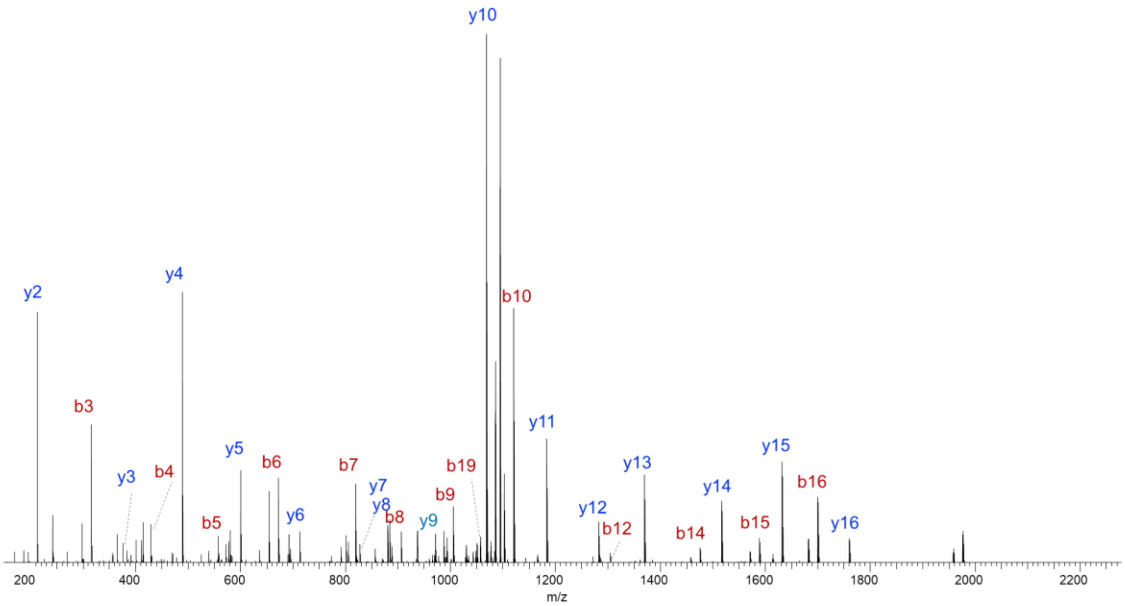

Figure S39. MS2 fragmentation of the cyclic CamA1-ILLY$_{113\text{-}132}$ product. Core peptide is highlighted in salmon. Stars (*) indicate the loss of two hydrogens in the MS2 fragment ions.

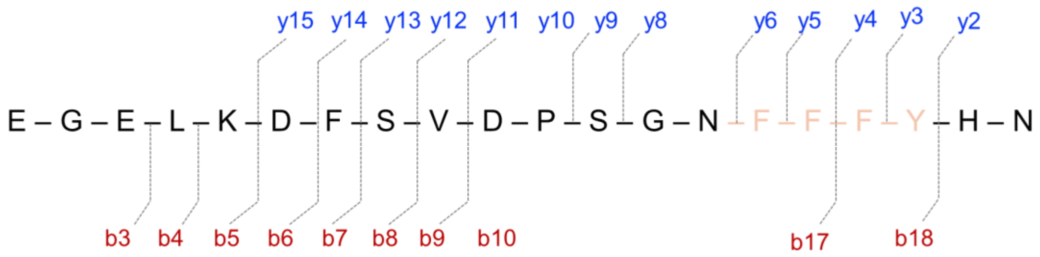

Fragment (z)	m (obs)	m (cal)	Δm (ppm)
b1(+1)	-	130.0499	-
b2(+1)	-	187.0713	-
b3(+1)	316.1142	316.1139	0.89
b4(+1)	429.1984	429.1980	0.98
b5(+1)	557.2945	557.2929	2.80
b6(+1)	672.3203	672.3199	0.62
b7(+1)	819.3895	819.3883	1.48
b8(+1)	906.4204	906.4203	0.09
b9(+1)	1005.4877	1005.4887	1.02
b10(+1)	1120.5158	1120.5157	0.12
b11(+1)	-	1217.5684	-
b12(+1)	-	1304.6005	-
b13(+1)	-	1361.6219	-
b14(+1)	-	1475.6649	-
b15(+1)	-	1622.7333	-
b16(+1)	-	1769.8017	-
b17(+1)	1916.8728	1916.8701	1.42
b18(+2)	1040.4765	1040.4703	5.91
b19(+2)	-	1108.9998	-
b20(+2)	-	1166.0213	-

Fragment (z)	m (obs)	m (cal)	Δm (ppm)
y1(+1)	-	133.0608	-
y2(+1)	270.1201	270.1197	1.14
Y3(+1)	433.1830	433.1830	0.12
y4(+1)	580.2527	580.2515	2.14
y5(+1)	727.3203	727.3199	0.59
y6(+1)	874.3878	874.3883	0.55
y7(+1)	-	988.4312	-
y8(+1)	1045.4520	1045.4527	0.64
y9(+1)	1132.4860	1132.4847	1.15
y10(+1)	1229.5373	1229.5375	0.13
y11(+1)	1344.5629	1344.5644	1.12
y12(+1)	1443.6335	1443.6328	0.48
y13(+1)	1530.6631	1530.6648	1.14
y14(+1)	1677.7348	1677.7333	0.92
y15(+1)	1792.7570	1792.7602	1.78
y16(+1)	-	1920.8552	-
y17(+2)	-	1017.4732	-
Y18(+2)	-	1081.9945	-
Y19(+2)	-	1110.5053	-
y20(+2)	-	1175.0266	-

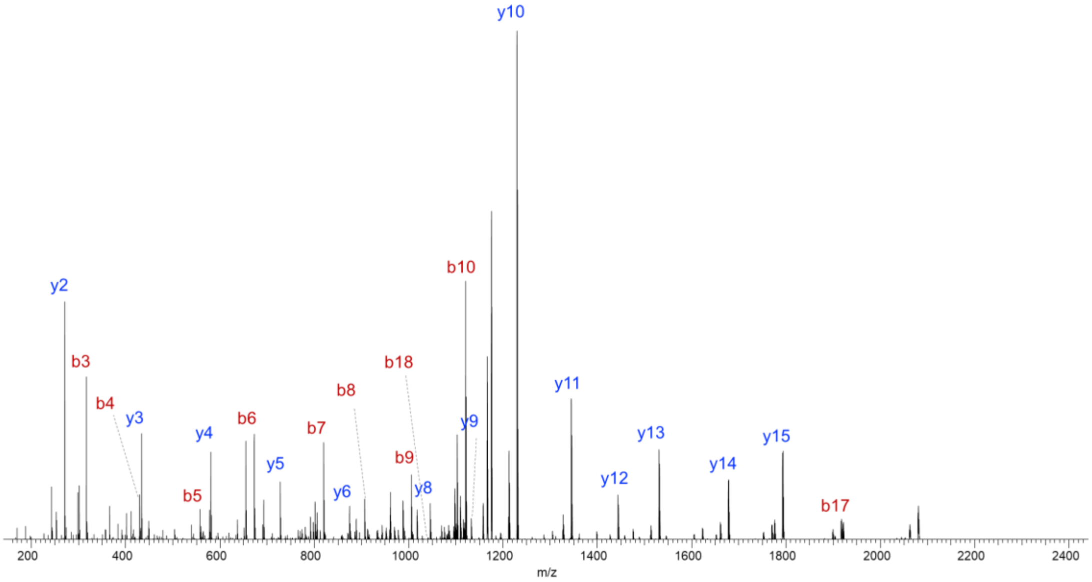

Figure S40. MS2 fragmentation of the linear CamA1-FFFY$_{20\text{-}40}$ substrate. Core peptide is highlighted in salmon.

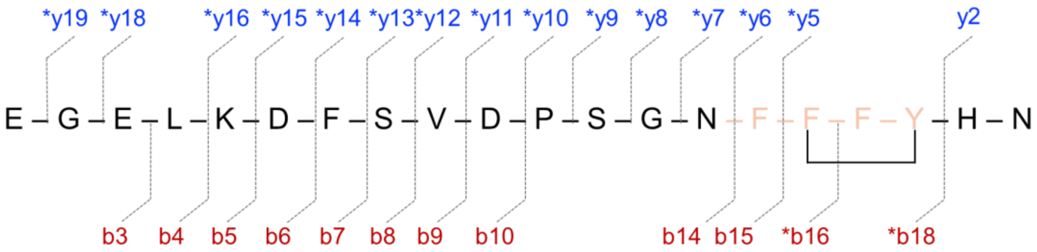

Fragment (z)	m (obs)	m (cal)	Δm (ppm)
b1	-	-	-
b2(+1)	-	187.0713	-
b3(+1)	316.1140	316.1139	0.38
b4(+1)	429.1983	429.1979	0.84
b5(+1)	557.2939	557.2929	1.79
b6(+1)	672.3210	672.3198	1.73
b7(+1)	819.3896	819.3882	1.65
b8(+1)	906.4214	906.4203	1.24
b9(+1)	1005.4897	1005.4887	1.00
b10(+1)	1120.5152	1120.5156	0.38
b11(+1)	-	1217.5685	-
b12(+1)	-	1304.6005	
b13(+1)	-	1361.6220	-
b14(+1)	1475.6633	1475.6648	1.02
b15(+1)	1622.7365	1622.7332	2.02
*b16(+1)	1767.7882	1767.7859	1.27
*b17(+2)	-	957.9309	-
*b18(+1)	2077.9274	2077.9177	4.67
*b19(+2)	-	1108.9998	-
*b20	-	-	-

Fragment (z)	m (obs)	m (cal)	Δm (ppm)
y1(+1)	-	2217.9877	-
y2(+1)	-	2160.9662	-
y3(+1)	-	2031.9236	-
y4(+1)	-	1918.8395	-
*y5(+1)	1790.7426	1790.7445	1.07
*y6(+1)	1675.7183	1675.7176	0.44
*y7(+1)	1528.6516	1528.6492	1.60
*y8(+1)	1441.6155	1441.6171	1.13
*y9(+1)	1342.5502	1342.5487	1.10
*y10(+1)	1227.5238	1227.5218	1.65
*y11(+1)	1130.4687	1130.4690	0.28
*y12(+1)	1043.4389	1043.4370	1.83
*y13(+1)	-	986.4155	-
*y14(+1)	872.3746	872.3726	2.29
*y15(+1)	725.3045	725.3042	0.43
*y16(+1)	580.2526	580.2515	1.96
*y17(+1)	-	433.1830	-
*y18(+1)	270.1196	270.1197	0.44
*y19(+1)	-	133.0608	-
*y20(+1)	-	-	-

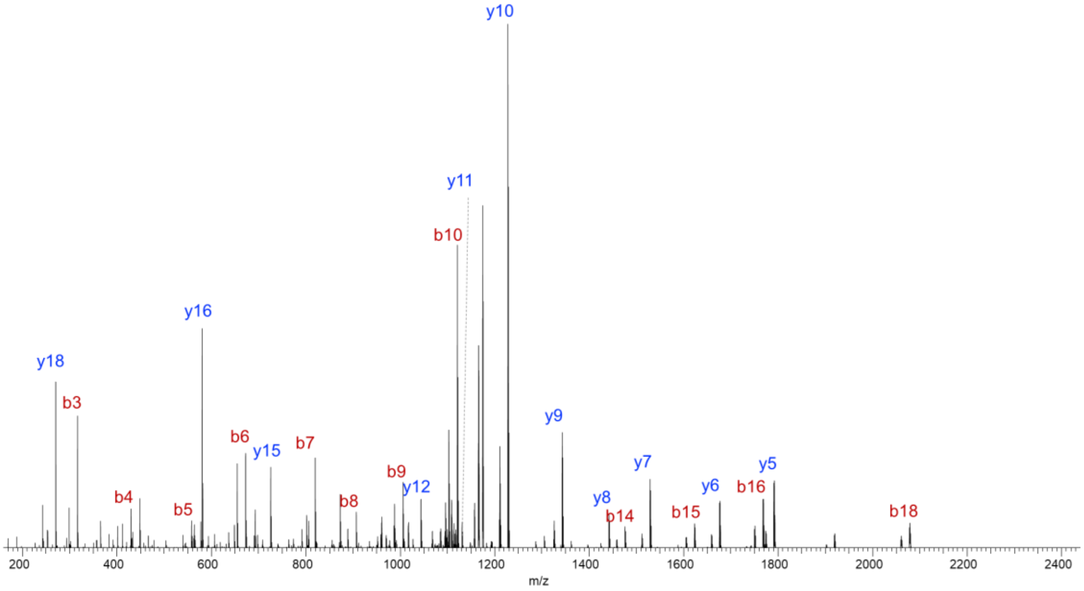

Figure S41. MS2 fragmentation of the cyclic CamA1-FFFY$_{20\text{-}40}$ product. Core peptide is highlighted in salmon. Stars (*) indicate the loss of two hydrogens in the MS2 fragment ions.

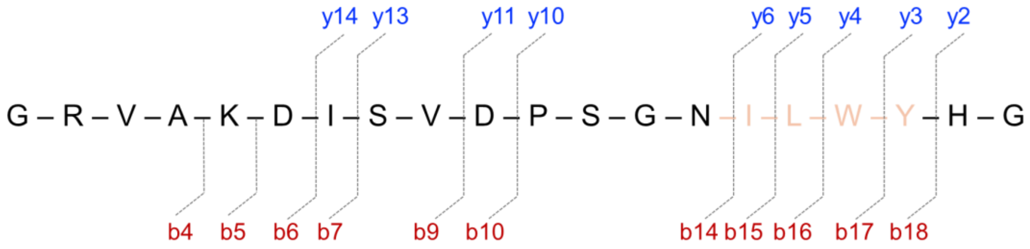

Fragment (z)	m (obs)	m (cal)	Δm (ppm)
b1(+1)	-	58.0287	-
b2(+1)	-	214.1298	-
b3(+1)	-	301.1282	-
b4(+1)	384.2342	384.2353	2.94
b5(+1)	512.3306	512.3303	0.61
b6(+1)	627.3589	627.3572	2.66
b7(+1)	740.4418	740.4413	0.69
b8(+1)	-	827.4733	-
b9(+1)	926.5411	926.5417	0.68
b10(+1)	1041.5682	1041.5687	0.45
b11(+1)	-	1138.6214	-
b12(+1)	-	1225.6535	-
b13(+1)	-	1282.6749	-
b14(+1)	1396.7182	1396.7178	0.25
b15(+1)	1509.8019	1509.8019	0.39
b16(+1)	1622.8901	1622.8860	2.54
b17(+1)	1808.9631	1808.9653	1.21
b18(+1)	1972.0378	1972.0286	4.66
b19(+1)	-	2109.0875	-
b20(+1)	-	2166.1090	-

Fragment (z)	m (obs)	m (cal)	Δm (ppm)
y1(+1)	-	76.0393	-
y2(+1)	213.0980	213.0982	1.17
Y3(+1)	376.1613	376.1616	0.74
y4(+1)	562.2421	562.2409	2.15
y5(+1)	675.3265	675.3249	2.30
y6(+1)	788.4112	788.4090	2.78
y7(+1)	-	902.4519	-
y8(+1)	-	959.4734	-
y9(+1)	-	1046.5054	-
y10(+1)	1143.5578	1143.5582	0.34
y11(+1)	1258.5874	1258.5851	1.41
y12(+1)	-	1357.6535	-
y13(+1)	1444.6876	1444.6856	1.41
y14(+1)	1557.7716	1557.7696	1.26
y15(+1)	-	1672.7966	-
y16(+1)	-	1800.8915	-
y17(+1)	-	1871.9286	-
y18(+1)	-	1970.9971	-
Y19(+1)	-	2127.0982	-
y20(+1)	-	2184.1196	-

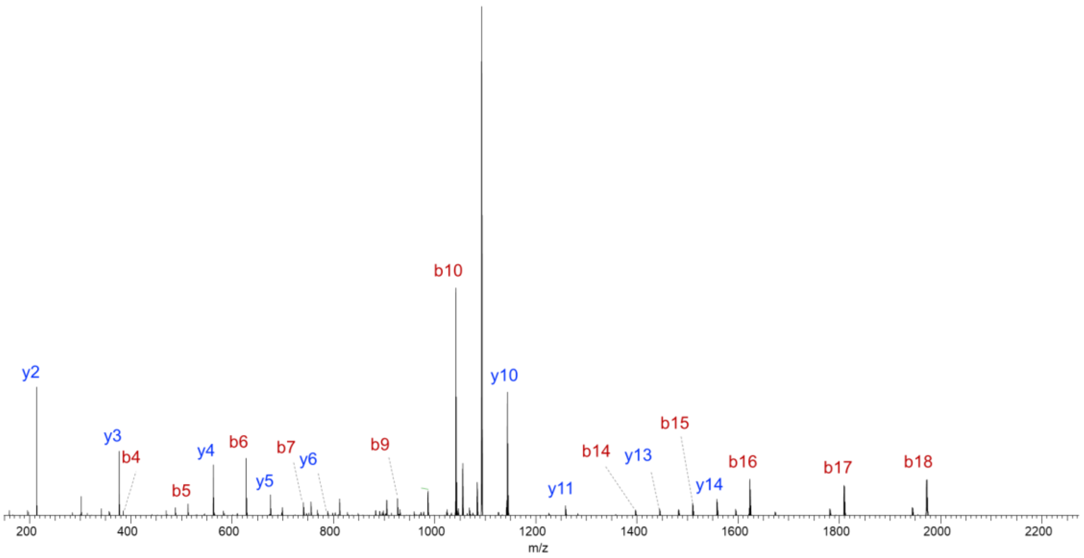

Figure S42. MS2 fragmentation of the linear CamA2-ILWY$_{56\text{-}75}$ substrate. Core peptide is highlighted in salmon.

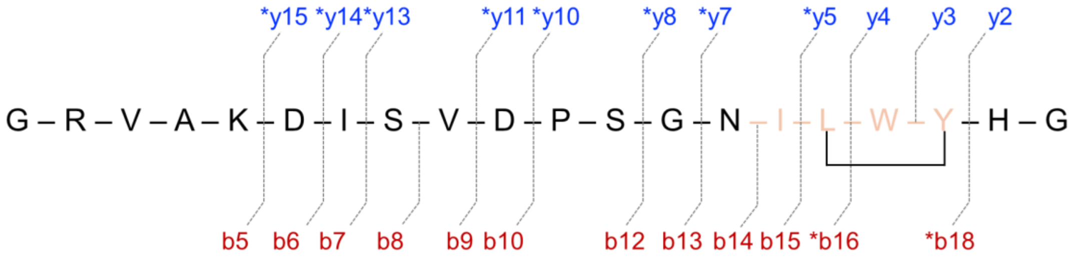

Fragment (z)	m (obs)	m (cal)	Δm (ppm)
b1(+1)	-	58.0287	-
b2(+1)	-	214.1298	-
b3(+1)	-	313.1982	-
b4(+1)	-	384.2353	-
b5(+1)	512.3308	512.3303	1.00
b6(+1)	627.3598	627.3572	4.10
b7(+1)	740.4432	740.4413	2.58
b8(+1)	827.4764	827.4733	3.72
b9(+1)	926.5411	926.5414	0.36
b10(+1)	1041.5697	1041.5687	0.99
b11(+1)	-	1138.6215	-
b12(+1)	1225.6537	1225.6535	0.20
b13(+1)	1282.6724	1282.6750	1.96
b14(+1)	1396.7182	1396.7174	0.32
b15(+1)	1509.8025	1509.8028	0.39
*b16(+1)	1620.8729	1620.8703	0.59
*b17(+1)	-	1806.9496	-
*b18(+1)	1970.0212	1970.0129	4.20
*b19(+1)	-	2107.0876	-
*b20(+1)	-	2164.0933	-

Fragment (z)	m (obs)	m (cal)	Δm (ppm)
y1(+1)	-	76.0393	-
y2(+1)	213.0981	213.0982	0.70
y3(+1)	376.1619	376.1616	0.85
y4(+1)	562.2418	562.2409	1.62
*y5(+1)	673.3113	673.3093	3.01
*y6(+1)	-	786.3933	-
*y7(+1)	900.4372	900.4363	1.04
*y8(+1)	957.4585	957.4577	0.81
*y9(+1)	-	1044.4897	-
*y10(+1)	1141.5423	1141.5425	0.18
*y11(+1)	1256.5702	1256.5694	0.60
*y12(+1)	-	1355.6379	-
*y13(+1)	1442.6694	1442.6699	0.34
*y14(+1)	1555.7532	1555.7540	0.48
*y15(+1)	1670.7747	1670.7809	3.70
*y16(+1)	-	1798.8758	-
*y17(+1)	-	1869.9130	-
*y18(+1)	-	1968.9814	-
*y19(+1)	-	2125.0825	-
*y20(+1)	-	-	-

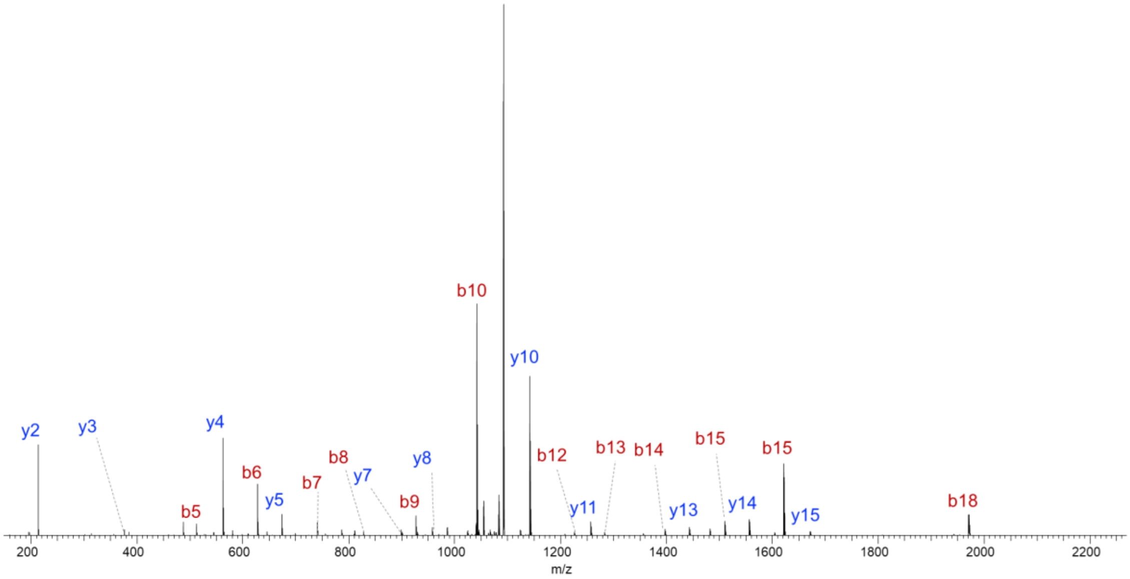

Figure S43. MS2 fragmentation of the cyclic CamA2-ILWY$_{56\text{-}75}$ product. Core peptide is highlighted in salmon. Stars (*) indicate the loss of two hydrogens in the MS2 fragment ions.

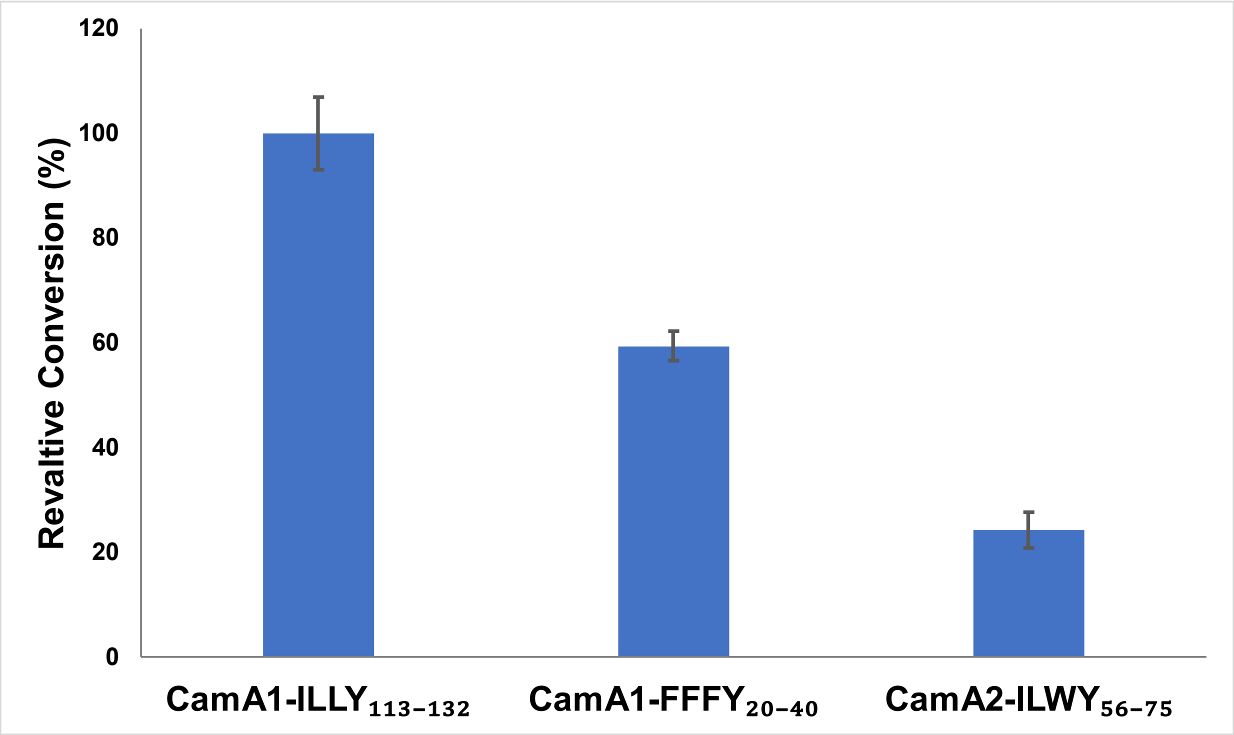

Figure S44. CamB1 substrate scope assay. Assays were performed in citrate-phosphate buffer (pH 8.0) in a stoichiometric ratio of 1:150 enzyme/substrate and quenched after 60 minutes. Error bars indicated the standard deviation of three trials.

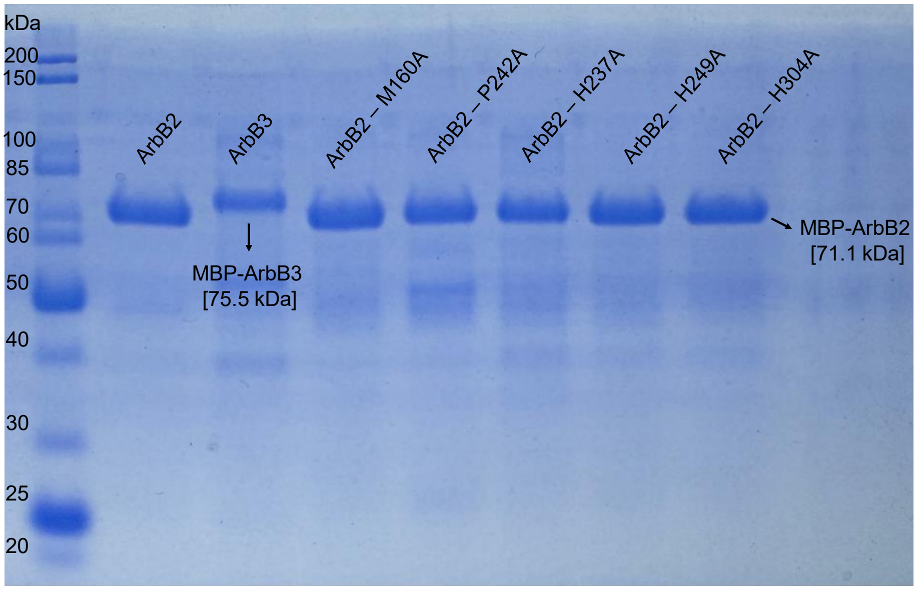

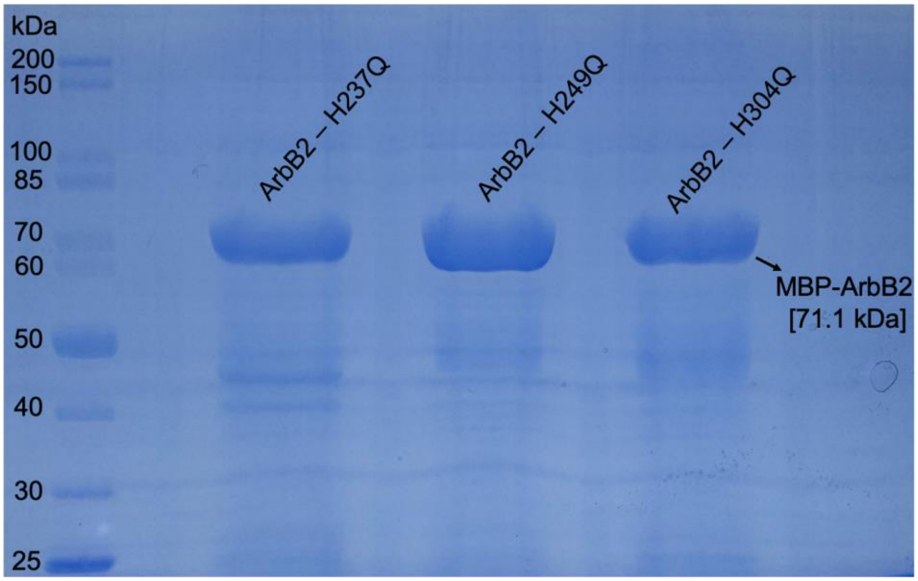

Figure S45. Heterologous expression of ArbB2. 12% SDS-PAGE gel analysis of MBP-ArbB2 and MBP-ArbB2 mutants after expression in *E. coli*, unfolding, purification through Ni affinity column, refolding through dialysis and purification by size exclusion chromatography (HiLoad 16/600 Superdex 200 and HiLoad 16/600 Superdex 75). (Top) First lane unstained Protein Standard, Broad Range (10-200 kDa) (New England Biolabs); second lane MBP-ArbB2$_{75\text{-}320}$; third lane MBP-ArbB3$_{23\text{-}288}$; fourth lane MBP-ArbB2$_{75\text{-}320}$ M160A; fifth lane MBP-ArbB2$_{75\text{-}320}$ P242A; sixth lane MBP-ArbB2$_{75\text{-}320}$ H237A; seventh lane MBP-ArbB2$_{75\text{-}320}$ H249A; eighth lane MBP-ArbB2$_{75\text{-}320}$ H304A. (Bottom) First lane unstained Protein Standard, Broad Range (10-200 kDa) (New England Biolabs); second lane MBP-ArbB2$_{75\text{-}320}$ P237Q; third lane MBP-ArbB2$_{75\text{-}320}$ H249Q; fourth lane MBP-ArbB2$_{75\text{-}320}$ H304Q. All MBP-ArbB2$_{75\text{-}320}$ samples were approximately 71.1 kDa as expected. MBP-ArbB3$_{23\text{-}288}$ was approximately 75.5 kDa as expected.

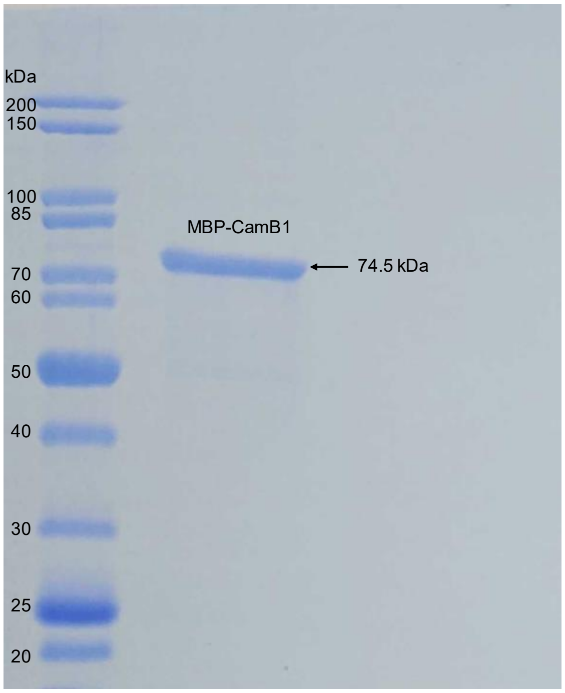

Figure S46. Heterologous expression of CamB1. 12% SDS-PAGE gel analysis of MBP-CamB1$_{23\text{-}278}$ after expression in *E. coli*, unfolding, purification through Ni affinity column, refolding through dialysis and purification by size exclusion chromatography (HiLoad 16/600 Superdex 200 and HiLoad 16/600 Superdex 75). First lane unstained Protein Standard, Broad Range (10-200 kDa) (New England Biolabs); second lane MBP-CamB1$_{23\text{-}278}$. Sample ran as expected at 74.5 kDa.

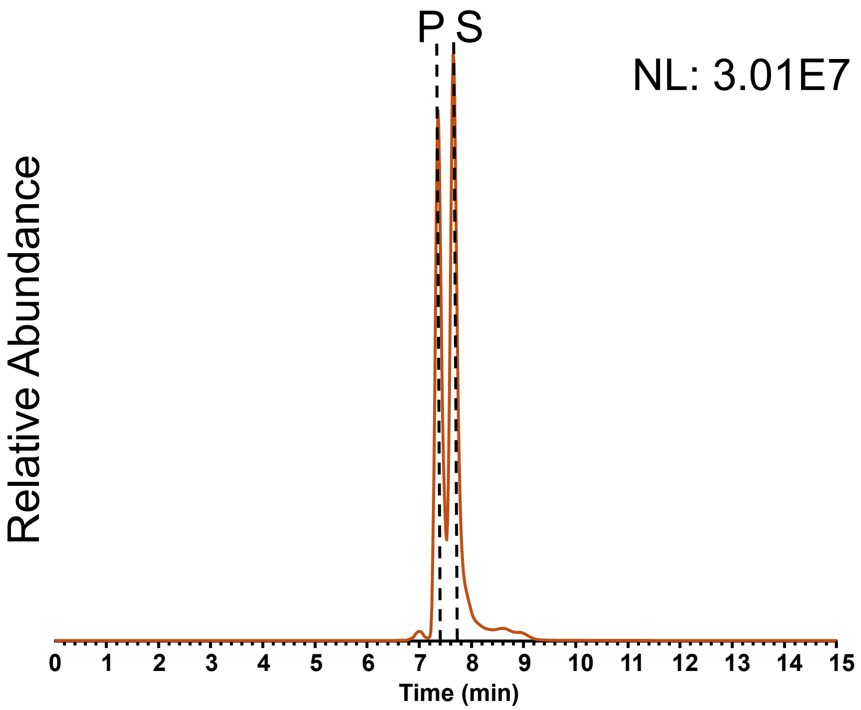

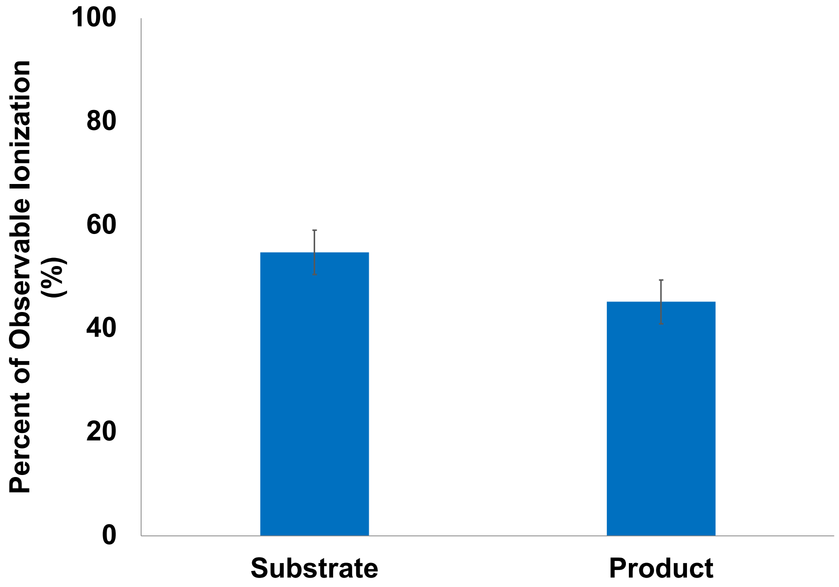

Figure S47. EIC traces showing relative abundance of the substrate ArbA2-FLWGY and its fully converted product in a 1:1 solution. Ionization efficiency comparing the percentage of observable ionization of the substrate ArbA2-FLWGY with the fully converted product. As expected, no significant difference in efficiency was observed. Error bars indicated the standard deviation of three trials. T-test provided a p-value of 0.91 which is >0.05 cut off showing differences in observed ionization to not be significant.

Supplementary Note

Using the catalytic ArbB2 system, we evaluated the importance of recognition sequence length using eight different precursor peptides. Peptides shorter than the 19mer exhibited diminished activity. These less active peptides correspond to truncations shorter than N(-12). According to modeling (**Figure S2**), 15 amino acid residues located upstream the core peptide closely interact with the enzyme surface, suggesting importance for binding. Extending the N-terminus of the precursor peptide beyond the G(-13) appeared to minimally improve the conversion. Conversely, the peptides that contained an extended C-terminus resulted either no or minimally improved. These results support the modeling and importance of the N-terminal recognition sequence.

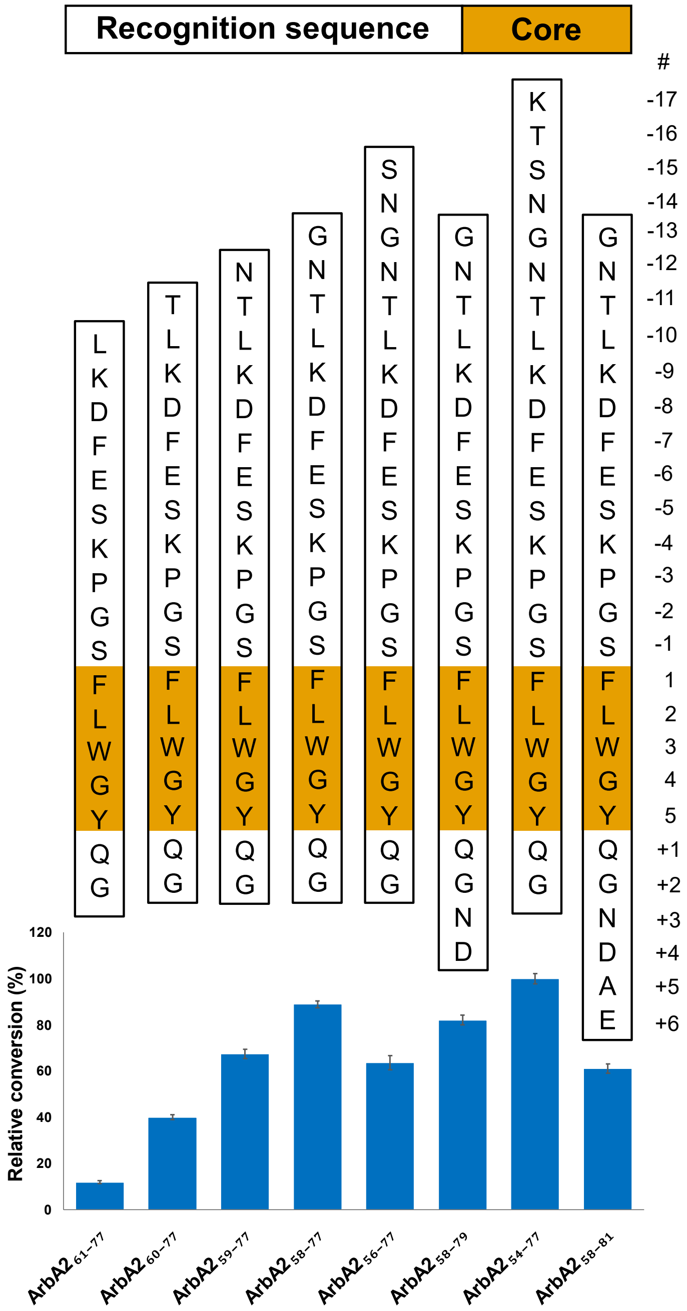

Figure S48. Alternative ArbA2-FLWGY peptide lengths. Assays were performed in citrate-phosphate buffer (pH 8.0), in a ratio of 1:150 enzyme/substrate, at room temperature, and quenched after 60 minutes. Lengths of 17, 18, 19, 20, 22, and 24 amino acids were examined. Additional 22 and 24 amino acid peptides extended at the C-terminal position were also tested. Error bars indicated the standard deviation of three trials.

References

(1) Abramson, J.; Adler, J.; Dunger, J.; Evans, R.; Green, T.; Pritzel, A.; Ronneberger, O.; Willmore, L.; Ballard, A. J.; Bambrick, J.; Bodenstein, S. W.; Evans, D. A.; Hung, C.-C.; O'Neill, M.; Reiman, D.; Tunyasuvunakool, K.; Wu, Z.; Žemgulytė, A.; Arvaniti, E.; Beattie, C.; Bertolli, O.; Bridgland, A.; Cherepanov, A.; Congreve, M.; Cowen-Rivers, A. I.; Cowie, A.; Figurnov, M.; Fuchs, F. B.; Gladman, H.; Jain, R.; Khan, Y. A.; Low, C. M. R.; Perlin, K.; Potapenko, A.; Savy, P.; Singh, S.; Stecula, A.; Thillaisundaram, A.; Tong, C.; Yakneen, S.; Zhong, E. D.; Zielinski, M.; Žídek, A.; Bapst, V.; Kohli, P.; Jaderberg, M.; Hassabis, D.; Jumper, J. M. Accurate Structure Prediction of Biomolecular Interactions with AlphaFold 3. *Nature* **2024**, *630* (8016), 493–500.

(2) Almagro Armenteros, J. J.; Tsirigos, K. D.; Sønderby, C. K.; Petersen, T. N.; Winther, O.; Brunak, S.; von Heijne, G.; Nielsen, H. SignalP 5.0 Improves Signal Peptide Predictions Using Deep Neural Networks. *Nat. Biotechnol.* **2019**, *37* (4), 420–423.

(3) Krogh, A.; Larsson, B.; von Heijne, G.; Sonnhammer, E. L. Predicting Transmembrane Protein Topology with a Hidden Markov Model: Application to Complete Genomes. *J. Mol. Biol.* **2001**, *305* (3), 567–580.

(4) Sonnhammer, E. L. L.; Von Heijne, G.; Krogh, A. A Hidden Markov Model for Predicting Transmembrane Helices in Protein Sequences. *Ismb* **1998**.(1) Abramson, J.; Adler, J.; Dunger, J.; Evans, R.; Green, T.; Pritzel, A.; Ronneberger, O.; Willmore, L.; Ballard, A. J.; Bambrick, J.; Bodenstein, S. W.; Evans, D. A.; Hung, C.-C.; O'Neill, M.; Reiman, D.; Tunyasuvunakool, K.; Wu, Z.; Žemgulytė, A.; Arvaniti, E.; Beattie, C.; Bertolli, O.; Bridgland, A.; Cherepanov, A.; Congreve, M.; Cowen-Rivers, A. I.; Cowie, A.; Figurnov, M.; Fuchs, F. B.; Gladman, H.; Jain, R.; Khan, Y. A.; Low, C. M. R.; Perlin, K.; Potapenko, A.; Savy, P.; Singh, S.; Stecula, A.; Thillaisundaram, A.; Tong, C.; Yakneen, S.; Zhong, E. D.; Zielinski, M.; Žídek, A.; Bapst, V.; Kohli, P.; Jaderberg, M.; Hassabis, D.; Jumper, J. M. Accurate Structure Prediction of Biomolecular Interactions with AlphaFold 3. *Nature* **2024**, *630* (8016), 493–500. https://doi.org/10.1038/s41586-024-07487-w.

(2) Krogh, A.; Larsson, B.; von Heijne, G.; Sonnhammer, E. L. Predicting Transmembrane Protein Topology with a Hidden Markov Model: Application to Complete Genomes. *J. Mol. Biol.* **2001**, *305* (3), 567–580. https://doi.org/10.1006/jmbi.2000.4315.

(3) Sonnhammer, E. L. L.; Von Heijne, G.; Krogh, A. A Hidden Markov Model for Predicting Transmembrane Helices in Protein Sequences. *Ismb* **1998**.

(4) Almagro Armenteros, J. J.; Tsirigos, K. D.; Sønderby, C. K.; Petersen, T. N.; Winther, O.; Brunak, S.; von Heijne, G.; Nielsen, H. SignalP 5.0 Improves Signal Peptide Predictions Using Deep Neural Networks. *Nat. Biotechnol.* **2019**, *37* (4), 420–423. https://doi.org/10.1038/s41587-019-0036-z.

(5) Lee, S. W.; Mitchell, D. A.; Markley, A. L.; Hensler, M. E.; Gonzalez, D.; Wohlrab, A.; Dorrestein, P. C.; Nizet, V.; Dixon, J. E. Discovery of a Widely Distributed Toxin Biosynthetic Gene Cluster. *Proceedings of the National Academy of Sciences* **2008**, *105* (15), 5879–5884. https://doi.org/10.1073/pnas.0801338105.
